# Synthesis of Isothiocyanates Using DMT/NMM/TsO^−^ as a New Desulfurization Reagent

**DOI:** 10.3390/molecules26092740

**Published:** 2021-05-06

**Authors:** Łukasz Janczewski, Dorota Kręgiel, Beata Kolesińska

**Affiliations:** 1 Faculty of Chemistry, Institute of Organic Chemistry, Lodz University of Technology, Zeromskiego 116, 90-924 Lodz, Poland; beata.kolesinska@p.lodz.pl; 2 Department of Environmental Biotechnology, Faculty of Biotechnology and Food Sciences, Lodz University of Technology, Wolczanska 171/173, 90-924 Lodz, Poland; dorota.kregiel@p.lodz.pl

**Keywords:** isothiocyanates, desulfurization agent, 4-(4,6-dimethoxy-1,3,5-triazin-2-yl)-4-methylmorpholinium toluene-4-sulfonate, DMT/NMM/TsO^-^, microwave-assisted synthesis of biologically active compounds, amino acids, circular dichroism, microwave synthesis, microwave technology, antibacterial activity

## Abstract

Thirty-three alkyl and aryl isothiocyanates, as well as isothiocyanate derivatives from esters of coded amino acids and from esters of unnatural amino acids (6-aminocaproic, 4-(aminomethyl)benzoic, and tranexamic acids), were synthesized with satisfactory or very good yields (25–97%). Synthesis was performed in a “one-pot”, two-step procedure, in the presence of organic base (Et_3_N, DBU or NMM), and carbon disulfide via dithiocarbamates, with 4-(4,6-dimethoxy-1,3,5-triazin-2-yl)-4-methylmorpholinium toluene-4-sulfonate (DMT/NMM/TsO^−^) as a desulfurization reagent. For the synthesis of aliphatic and aromatic isothiocyanates, reactions were carried out in a microwave reactor, and selected alkyl isothiocyanates were also synthesized in aqueous medium with high yields (72–96%). Isothiocyanate derivatives of L- and D-amino acid methyl esters were synthesized, under conditions without microwave radiation assistance, with low racemization (er 99 > 1), and their absolute configuration was confirmed by circular dichroism. Isothiocyanate derivatives of natural and unnatural amino acids were evaluated for antibacterial activity on *E. coli* and *S. aureus* bacterial strains, where the most active was ITC **9e.**

## 1. Introduction

Isothiocyanates (ITCs) with the general formula R-N=C=S can be considered as compounds derived from biologically inactive glucosinolates [1,2,3,4]. They are produced by cruciferous vegetables such as broccoli, radishes, wasabi, and cauliflower as part of their defense mechanisms [5,6]. In response to damage to the plant, glucosinolates are converted into intermediate aglucon derivatives, which are converted into the final ITCs by a biochemical process according to the Lossen rearrangement [7]. Myrosinase, which is present in separate organelles and even in the cells of undamaged cruciferous plant tissue, plays a role in this transformation [8]. Isothiocyanateshave many desirable characteristics, including primarily anti-proliferative properties [9,10,11,12,13,14,15]. Like naturally occurring isothiocyanates (e.g., sulforaphane [16,17,18,19,20] (SFN), phenethyl isothiocyanate [21] (PEITC), or benzyl isothiocyanate [22] (BITC)), their synthetically modified analogs with the structure of a phosphorus atom [23,24] or fluorine atom [25,26] also exhibit anti-proliferative activity. Natural as well as synthetic ITCs may additionally have anti-bacterial [27,28,29,30], anti-fungal [31], and anti-glioblastoma effects [32]. They are used in proteomics as probes [33,34], and in organic synthesis for the preparation of thioureas [35], thioamides [36], and heterocyclic compounds [37,38].

**Figure 1:**
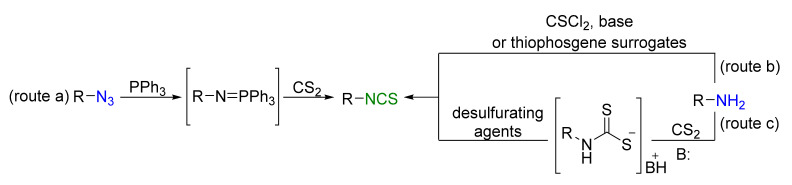
Methods of synthesizing isothiocyanates.

In recent years, several new methods have been developed for the synthesis of ITCs, using fluorine-containing reagents such as Langlois reagent (F_3_CSO_2_Na) [39], Ph_3_P^+^CF_3_CO_2_^−^ (PDFA)/S_8_ [40], (Me_4_N)SCF_3_ [41], CF_3_SiMe_3_/S_8_ or AgSCF_3_ [42], and BrCF_2_CO_2_Na/S_8_ [43]. However, three methods still predominate. The most common method is the Staudinger/aza-Wittig tandem reaction, in which the starting azides react with triphenylphosphine and are converted into iminophosphorane intermediates. Then, in the presence of carbon disulfide, the iminophosphate intermediates are transformed into the final ITCs (Figure 1, route a) [44,45,46,47]. The second method involves the use of a primary amine and thiophosgene as a reagent for the transfer of the thiocarbonyl moiety in the presence of a base (Figure 1, route b) [48,49,50,51]. Due to the toxicity of thiophosgene and the sensitivity of certain functional groups, this reagent is increasingly replaced by surrogates such as di-(2-pyridyl) thionocarbamate [52], 1,1′-thiocarbonyldiimidazole [53], or 1,1′-thiocarbonyldi-2-(1*H*)-pyridone [54]. The third method is a two-step one-pot procedure. Primary amines are transformed in the presence of a base and carbon disulfide into intermediate dithiocarbamates, which after treatment with the desulfurating agent are transformed into the target ITCs (Figure 1, route c).

Many desulfurating agents are currently available. Their choice depends on the reaction conditions and the presence of functional groups in the reagents. The most often used desulfurating agents include tosyl chloride [55], sodium persulfate [56], iodine [57], mesyl chloride [58], ethyl chloroformate [59], di-*tert*-butyl dicarbonate [60], cyanuric chloride [61], and others [62]. Microwave radiation has been found to support the conversion of dithiocarbamates into ITCs [63]. Coupling reagents used in peptide chemistry, such as HBTU and PyBOP [64], DCC [65], and T3P^®^ [66], can also be used as desulfurating reagents. However, despite the availability of numerous reagents, new compounds are continually sought to provide for the efficient synthesis of structurally diverse ITCs from dithiocarbamates, supported by microwave radiation.

**Figure 2:**
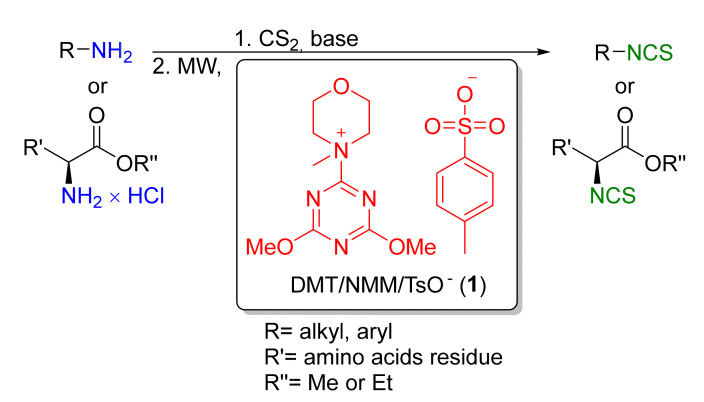
Synthesis of ITCs using DMT/NMM/TsO^−^ (**1**).

Here, we describe the microwave-assisted synthesis of structurally diverse aliphatic and aromatic isothiocyanates, as well as normal synthesis of isothiocyanate derivatives of natural and unnatural amino acids, using 4-(4,6-dimethoxy-1,3,5-triazin-2-yl)-4-methylmorpholinium toluene-4-sulfonate (DMT/NMM/TsO^−^, **1**) as a new, efficient desulfurating agent for the indirect formation of dithiocarbamates from primary amines or hydrochlorides (Figure 2).

DMT/NMM/TsO^−^ [67,68] (**1**) is an effective, and environmentally friendly coupling reagent used for the synthesis of amides, esters, and peptides in solution or solid phase, as well as under microwave-assisted conditions [69]. It enables the synthesis of peptides using Z-, Boc-, and Fmoc- protected substrates as well as unnatural amino acids. Using DMT/NMM/TsO^−^, synthesis of peptides occurs rapidly, with high yields, without racemization, and often with sufficiently high purity to avoid the necessity of additional chromatographic purification. Use of DMT/NMM/TsO^−^ in the synthesis of structurally diverse isothiocyanates characterized by a wide range of biological activity could allow for the efficient synthesis of peptide conjugates with isothiocyanates, requiring only a single coupling reagent. Reducing the numbers of compounds utilized in the synthesis of biologically active compounds should reduce the number and variety of by-products, which is particularly important for the impurity profile.

## 2. Results and Discussion

### 2.1. Optimizing the Synthesis of Aliphatic Isothiocyanates

In the first stage of the research, it was checked whether DMT/NMM/TsO^−^ (**1**) acts as a desulfurating agent, and experiments to optimize the reaction conditions were carried out. Phenethylamine (**2a**) was used as a model amine in the optimization tests. Use of carbon disulfide (CS_2_) (3 equiv.) in the presence of triethylamine (Et_3_N) (4 equiv.) in DCM as the solvent enabled formation of intermediate dithiocarbamate **3a** in just 5 min at room temperature (rt) [63]. The obtained dithiocarbamate **3a** was subjected to a microwave-assisted reaction with 1 equiv. of DMT/NMM/TsO^−^ (**1**) as a desulfurating agent, producing the target isothiocyanate **4a** with 90% yield in 3 min at 90 °C (initial 200 W power) (Table 1, entry 1). 

Extending the reaction time to 5 min and 10 min did not further increase the yield (Table 1, entry 1, footnote c). Next, we investigated the influence of the base on the yield. Replacing triethylamine with *N*-methylmorpholine (NMM) or 1,8-diazabicyclo[5.4.0]undec-7-ene (DBU) resulted in lowering of the yield to 78% and 69%, respectively (Table 1, entry 2 and 3), whereas the reaction without base resulted in final isothiocyanate **4a** with only 51% yield (Table 1, entry 4). Decreasing the amount of Et_3_N to 3 equiv. resulted in a slight increase the yield, to 92% (Table 1, entry 5). Increasing the amount of coupling reagent **1** to 1.3 equiv. (Table 1, entry 6) and to the 1.6 equiv. (Table 1, entry 6, footnote d) did not affect the yield, which remained at 92% in both cases. However, the reduction of DMT/NMM/TsO^−^ (**1**) to 0.7 equiv. reduced the yield to 86% (Table 1, entry 7). The reaction without desulfurating agent **1** produced **4a** with only 55% yield (Table 1, entry 8). In the next step, we investigated the possibility of performing the reaction in aqueous media, using optimized conditions: 3 min, 90 °C, CS_2_ (3 equiv.), Et_3_N (3 equiv.) as a base, with DMT/NMM/TsO^−^ (1 equiv.). In these conditions, ITC **4a** was obtained in a satisfactory yield 73% (Table 1, entry 9). It has been found that increasing the amount of agent **1** to 1.3 equiv. allowed us to obtain isothiocyanate **4a** with a very high yield of 89% (Table 1, Entry 10). Further increasing the amount of **1** to 1.6 equiv. did not affect the yield of the reaction (Table 1, entry 10, footnote e). We also show that this reaction occurs in normal conditions at rt, although in lower yield. Performing the reaction in 30 min at rt resulted in obtaining the product **4a** with 82% yield (Table 1, Entry 11). Prolonging the time to 60 min did not affect the results, but shortening the time to 10 min resulted in a decrease in the yield to 79% (Table 1, entry 11, footnote g). Additionally, performing the reaction in the sealed tube for 30 min at 90 °C (Table 1, entry 11, footnote h) did not affect the yield of reaction, which was still 82%. The results of experiments in normal conditions at room and at high temperature undoubtedly indicate that the use of microwave radiation assistance leads to higher yields of the ITC **4a**, and also that it is possible to significantly shorten the reaction time.

**Figure 3:**
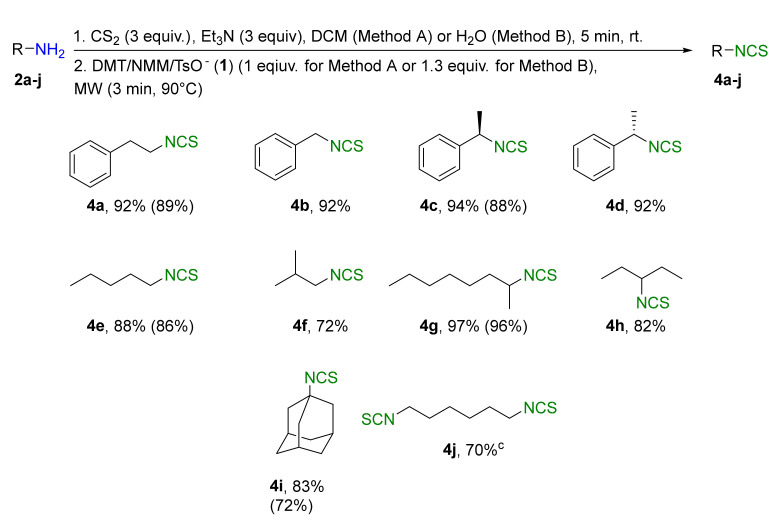
Scope of aliphatic isothiocyanates **4a**–j^a,b^. ^a^-**Method A**: conditions: amine **2a**–**j** (2 mmol), Et_3_N (3 equiv., 6 mmol), CS_2_ (3 equiv., 6 mmol), DCM (3 mL), first step 5 min, rt, then DMT/NMM/TsO^−^ (**1**) (2 mmol, 1 equiv.); MW irradiation in standard mode 3 min, 90 °C. Yield of products isolated after flash chromatography (hexane). **Method B**: conditions: amine **2a**, **2c**, **2e**, **2g**, and **2i** (2 mmol), Et_3_N (3 equiv., 6 mmol), CS_2_ (3equiv., 6 mmol), H_2_O (3 mL); first step 5 min, rt, then DMT/NMM/TsO^−^ (**1**) (2.6 mmol, 1.3 equiv.); MW irradiation in standard mode 3 min, 90 °C; ^b–^yield in parenthesis applies to the reaction performed in H_2_O; ^c^–conditions: amine **2j** (2 mmol), Et_3_N (6 equiv., 12 mmol), CS_2_ (6 equiv., 12 mmol), DCM (5 mL), DMT/NMM/TsO^−^ (**1**) (4 mmol, 2 equiv.).

**Figure 4:**
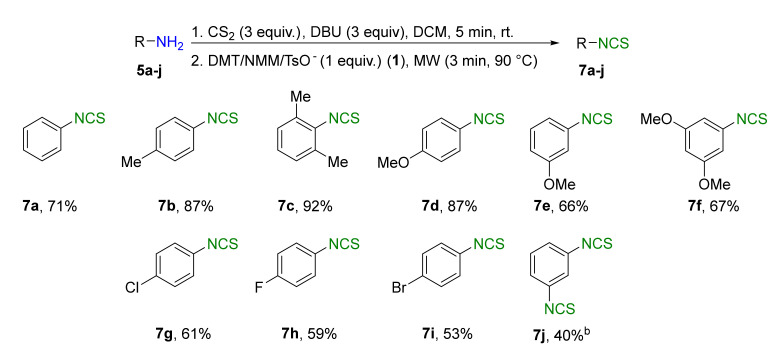
Scope of aromatic isothiocyanates**7a–j**^a^. ^a^–conditions: amine **5a**–**j** (2 mmol), DBU (3 equiv., 6 mmol), CS_2_ (3 equiv., 6 mmol), DCM (3 mL), first step 5 min, rt, then DMT/NMM/TsO^−^ (**1**) (2 mmol, 1 equiv.); MW irradiation in standard mode 3 min, 90 °C. Yield of products isolated after flash chromatography (hexane); ^b^–conditions: amine **5j** (2 mmol), DBU (6 equiv. 12 mmol), CS_2_ (6 equiv. 12 mmol), DCM (5 mL), DMT/NMM/TsO^−^ (**1**) (4 mmol, 2 equiv.).

**Figure 5:**
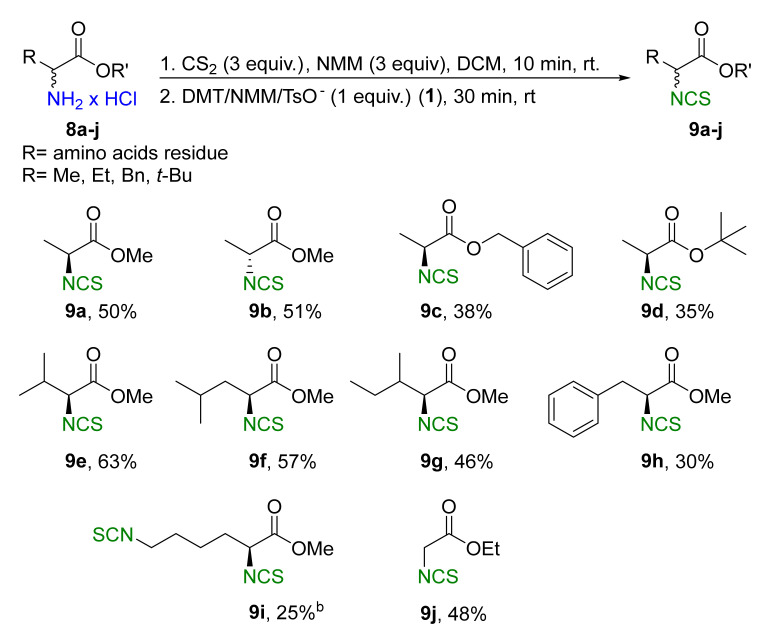
Scope of isothiocyanate derivatives of amino acids **9a**–**j**^a^. ^a^-conditions: hydrochloride **8a**–**j** (2 mmol), NMM (0.66 mL, 3 equiv., 6 mmol), CS_2_ (0.36 mL, 3 equiv., 6 mmol), DCM (5 mL); first step 10 min, rt, then DMT/NMM/TsO^−^ (**1**) (0.828 g, 1 equiv., 2 mmol,), normal conditions, rt., 30 min. Products isolated after flash chromatography (hexane:EtOAc 20:1); ^b^–conditions: hydrochloride **8i** (2 mmol), NMM (1.32 mL, 6 equiv., 12 mmol), CS_2_ (0.72 mL, 6 equiv., 12 mmol), DCM (5 mL); first step 10 min, rt, then DMT/NMM/TsO^−^ (**1**) (1.656 g, 2 equiv., 4 mmol,), normal conditions, rt., 30 min. Products isolated after flash chromatography (hexane:EtOAc 20:1).

**Figure 6:**
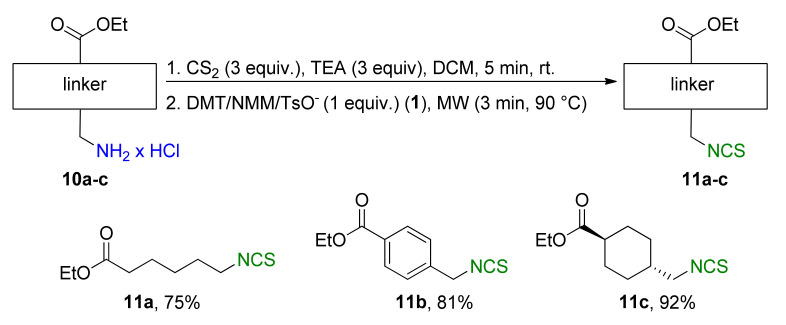
Synthetized isothiocyanates **11a**–**c** derived from unnatural amino acids ^a^. ^a^-conditions: hydrochloride **10a**–**c** (2 mmol), Et_3_N (0.83 mL, 3 equiv., 6 mmol), CS_2_ (0.36 mL, 3 equiv., 6 mmol), DCM (5 mL); first step 5 min, rt, then DMT/NMM/TsO^−^ (**1**) (0.828 g, 1 equiv., 2 mmol,), MW irradiation in standard mode 3 min, 90 °C. Yield of products after flash chromatography (hexane:EtOAc 10:1).

A library of isothiocyanates **4a–j**, **7a–j**, **9a–j**, and **11a–c** using structurally various aliphatic amines **2a**–**j** (Figure 3), aromatic amines **5a– j** (Figure 4), hydrochlorides of methyl and the ethyl esters of L- and D-amino acids **8a–j** (Figure 5), as well as hydrochlorides of the ethyl esters of unnatural amino acids **10a–c** (Figure 6), has been synthesized. All the compounds were isolated with good or very good yields (97–25%) after purification on a short pad of silica gel.

### 2.2. Synthesis of Aliphatic Isothiocyanates ***4a–j***

Aliphatic isothiocyanates were synthesized in a microwave reactor using two methods with different solvents. In Method A, DCM was used as solvent, whereas in Method B, the synthesis of selected ITCs was performed in H_2_O (Figure 3).

In Method A, isothiocyantes with an aromatic ring in the alkyl chain **4a**–**d** were obtained with up to 94% yield. Moreover, the synthesis of (*R*)-1-isothiocyanatoethylbenzene (**4c**) and (*S*)-1-isothiocyanatoethylbenzene (**4d**) from optically active amine **2c** and **2d** occurred without racemization (>99: 1 er). Yields of alkyl ITCs **4e** and volatile **4f** were slightly lower, at 88% and 72%, respectively. The yields of ITCs obtained from amines **2g**–**h** and 1-adamantylamine (**2i**), which are less volatile and more sterically hindered, rose to 97% for **4g**, 82% for **4h**, and 83% for **4i**. In addition to compounds with one –NCS group, an ITC with two –NCS groups (1,6-diisothiocyanatohexane, **4j**) was synthesized with high yield (70%). In order to investigate the scope and limitations of the possible synthesis of ITCs in water (Table 1, entry 14), the reaction was additionally performed for selected amines **2a**, **2c**, **2e**, **2g,** and **2i** in an aqueous media (Method B). The yields of ITCs **4a**, **4c**, **4e**, **4g,** and **4i** were a few percent lower than in DCM, but still acceptably high, and they were in the range of 96–72%.

Comparing the yields of aliphatic ITCs obtained in optimal conditions in Method A with previous results using only microwave radiation to synthesis of aliphatic ITCs [63], it can be concluded that application of DMT/NMM/TsO^−^ (**1**) as a desulfurating agent allows us to receive the final isothiocyanates **4a–i** with higher yield. The biggest yield difference was found for ITC **4f** (72% vs. 50% [63]), and the smallest for ITC **4i** (83% vs. 82% [63]). For other ITCs, except **4j**, which was not previous obtained, the yields were higher, from 5 to 14%, than in previous publication [66].

### 2.3. Synthesis of Aromatic Isothiocyanates ***7a–j***

In the next stage of the research, attempts were made to check the usefulness of DMT/NMM/TsO^−^ (**1**) as a desulfurating agent in reactions with aromatic amines. Performing the reaction under optimized conditions (Table 1, entry 5 and Figure 3 Method A) with aniline (**5a**) resulted in a low yield of final phenyl isothiocyanate (**7a**) at 30% (Table 2, entry 1). For this reason, the synthesis of aromatic ITCs required optimization (Table 2).

Replacing Et_3_N with DBU increased the yield to 71% (Table 2, entry 2). Increasing the amount of DBU to 4 equiv. or the desulfurating agent **1** to 1.3 equiv. did not improve to the yield (Table 2, entries 3 and 4, respectively). Prolonging the time to 10 min decreased the yield to 67% (Table 2, entry 5). A library of aromatic isothiocyanates **7a–j** substituted at *ortho*-, *meta*-, or *para*-positions was obtained under optimized conditions: 3 min, 90 °C, CS_2_ (3 equiv.), DBU (3 equiv.), DMT/NMM/TsO^−^ (1 equiv.). The yields of the final products were in the range of 40–92% (Figure 4).

Introducing the methyl group to the aromatic ring in the *para* position increased the yield of ITC **7b** to 87%, whereas introducing two methyl groups in positions 2 and 6 resulted in a slight increase of the yield of **7c** to 92%. Replacing the methyl with a methoxy group in the *para* position gave ITC **7d** with 87%. However, the methoxy group in the *metha* position as well as the presence of two dimethoxy groups in positions 3 and 5 in the aromatic ring diminished the yield of ITCs **7e** and **7f** to 66% and 67%, respectively. Application of this protocol to halogenoanilins **5g**–**i** enabled ITCs **7g**–**i** to be obtained with 61–53% yields. Aromatic isothiocyanate with two –NCS group – 1,3-diisothiocyanatobenzene (**7j**) were also obtained, although with a moderate yield of 40%. The synthesis of aromatic isothiocyanates in aqueous medium has failed. Model phenyl isothiocyanate (**7a**) was obtained with a low yield of 21% (Table 2, entry 6), and the synthesis of other aromatic isothiocyanates in H_2_O was not carried out. Comparing the yields of obtained aromatic isothiocyanates **7a**, **7c**–**e**, and **7g**–**j** in microwave-assisted synthesis with desulfurating agent **1** and without it [63], we can conclude that in almost all cases, the lack of desulfurating agent resulted in better yields of final isothiocyanates. The yields of ITCs **7a**, **7c**, **7e**, and **7g**–**j** in reaction without desulfurating agent were from 6 to 42% higher than yields with DMT/NMM/TsO^−^. The biggest difference was for ITC **7i** (53% vs. 95% [63]). Only ITC **7d** was obtained with higher yield in reaction with agent **1** than without it (87% vs. 75% [63]). It can conclude that in contrast to aliphatic isothiocyanates, aromatic iothiocyanates do not need addition of desulfurating agent, however its presence does not cause a significant drop of yield.

### 2.4. Synthesis of Isothiocyanate Derivatives of Natural ***9a–j*** and Unnatural ***11a–c*** Amino Acids

In next stage of the research, we attempted to synthesize ITCs using hydrochlorides of methyl and ethyl esters of L- and D-amino acids **8a**–**j** as substrates. As Et_3_N causes partial racemization of products [66], it was replaced with NMM. Performing the reaction on model L-alanine methyl ester hydrochloride (**8a**) under optimal conditions (3 min, 90 °C) in a microwave reactor produced **9a** with a yield of 59% and concomitant high racemization (er 65:35). To prevent the loss of enantiomeric homogeneity, the reaction was performed under normal conditions (30 min, rt). The product (*S*)-methyl 2-isothiocyanatopropanoate (**9a**) was obtained with 50% yield and low racemization (er 99 > 1) (Figure 5). Using these optimal conditions, a second enantiomer (*R*)-methyl 2-isothiocyanatopropanoate (**9b**) was produced with 51% yield and low racemization (er 99 > 1), using D-alanine methyl ester hydrochloride (**8b**) as a substrate. In addition to hydrochlorides of methyl esters of alanine, L-alanine benzyl ester hydrochloride (**8c**), and *tert*-butyl ester of L-alanine hydrochloride (**8d**), hydrochlorides of methyl esters of L-valine (**8e**), L-leucine (**8f**), L-isoleucine (**8g**), L-phenylalanine (**8h**), and L-lysine (**8i**), as well as the achiral hydrochloride of the ethyl ester of glycine (**8j**), were used as starting materials. Figure 5 presents the results of the synthesis of isothiocyanate derivatives of amino acids **9a**–**j**.

Isothiocyanates derived from the benzyl ester **9c** and *tert*-butyl ester **9d** of the L-alanine group were synthesized with lower yields of 38% and 35%, respectively. This may have been due to the presence of a larger ester group and thus greater steric hindrance. Isothiocyanate analogs of L-valine **9e**, L-leucine **9f**, L-isoleucine **9g**, and L-phenylalanine **9h** with isopropyl, isobutyl, *sec*-butyl, and the benzyl moiety, respectively, were synthesized with satisfactory yields (30–63%). During the synthesis of these compounds, it was observed that the yield depended on the effect of steric hindrance (steric hindrance **9h**>**9g**>**9f**>**9e**) by the substituent on the amino acid side chain (yield of **9e**>**9f**>**9g** >**9h**). In the case of the achiral ethyl ester of the isothiocyanates of glycine **9j**, the yield of the reaction was 48%. As in the case of the aliphatic and aromatic isothiocyanates with two –NCS groups also an isothiocyanate derivative from L-lysine **9i** with two –NCS groups was obtained, although with a low yield (25%). ITCs **9a**, **9b**, and **9h** were previous synthesized using as a desulfurating agent T3P^®^ also in normal conditions [66]. ITCs **9a** and **9b** were obtained with 10% better yield than with DMT/NMM/TsO^−^, however, ITC **9h** was synthesized with definitely higher yield – 72% [66]. Others ITCs were not synthesized using T3P^®^.

In addition to methyl and ethyl esters of natural amino acids, hydrochlorides of ethyl esters of unnatural amino acids with aliphatic, aromatic, and cyclohexyl linkers were used as substrates for the synthesis ITCs. Hydrochlorides of ethyl esters of 6-aminocaproic acid (**10a**), 4-(aminomethyl)benzoic acid (**10b**) and *trans*-4-(aminomethyl)cyclohexanecarboxylic acid (Tranexamic acid) (**10c**) were converted into target ITCs **11a**–**c** in microwave-assisted synthesis under optimal conditions (3 min, 90 °C), with DMT/NMM/TsO^−^ (**1**) as the desulfurating agent. All ITCs **11a**–**c** were isolated after flash chromatography with a very good yields and high purity (Figure 6). In the case of compound **11c** which is *trans*-isomer the synthesis occurred without racemization, due to the lack of signals of *cis*-isomer on ^1^H NMR spectrum.

Parental amino acids of substrates **10a**–**c** are characterized by hemostatic properties [70,71,72,73,74]. The biological activities of ITCs **11a**–**c** were also tested and described below.

### 2.5. Determination of the Absolute Configuration Using Circular Dichroism

**Figure 7:**
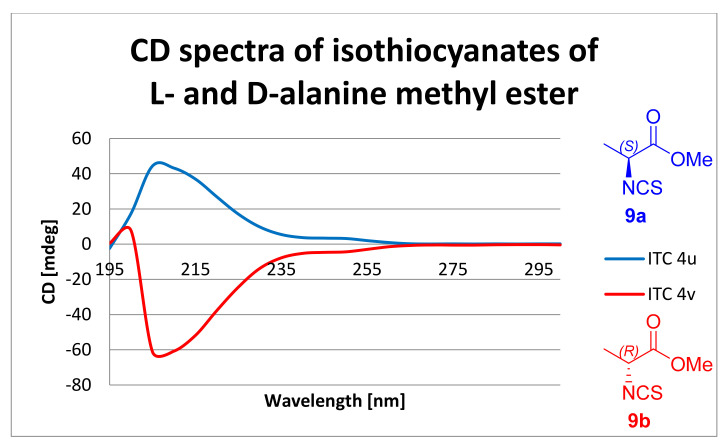
CD spectra of ITCs **9a** and **9b**.

Inspired by Michalski and Cież [75], we attempted to determine the absolute configuration of chiral ITCs **4c**–**d** and **9a**–**i** using circular dichroism (CD). All samples were dissolved in methanol. The concentrations of the samples were between 0.52 mg/mL and 0.30 mg/mL. For all *S* enantiomers of the ITCs analogs of L-amino acids **9a** and **9c**–**i**, the Cotton effect (CE) was positive. For the *R* enantiomer of the ITC analog of D-alanine **9b** the CE was negative. These results support the literature data [75,76]. The ITC **9a** with the *S* configuration at C-2 exhibited a strong positive CE at 205 nm, and the second enantiomer ITC **9b** with an *R* configuration at C-2 exhibited a strong negative CE, also at 205 nm. The CE for ITCs **9a** and **9b** were similar but reversed, confirming the presence of two different enantiomers (Figure 7).

For the other two ITC analogs of L-alanine with a benzyl **9c** or *tert*-butyl **9d** ester moiety, strong positive CE were observed at 205 nm and 210 nm, respectively (see Supporting Material Figures S77 and S78). A strong positive CE for ITCs **9e**–**g** was also observed at 210 nm (see Supporting Material Figures S79–S81). However, for ITC **9h**, a strong positive CE was observed at 225 nm (see Supporting Material Figure S82), which could be due to the presence of an aromatic ring. For ITC **9i** with two –NCS groups, a strong positive CE was observed at 210 nm (see Supporting Material Figure S83).

**Figure 8:**
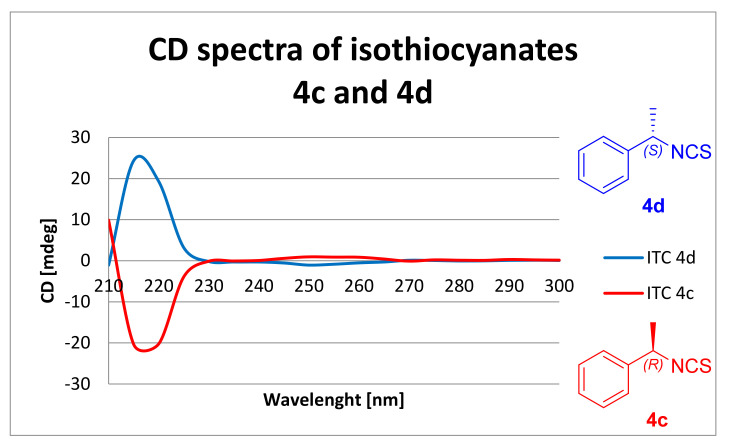
CD spectra of ITCs **4c** and **4d**.

As in the case of the chiral ITCs analogs of L- and D-amino acids **9a**–**i,** the absolute configuration of the chiral ITCs with a benzyl moiety **4c** and **4d** was also determined using CD (Figure 8).

For both enantiomers **4c** and **4d**, a strong CE was observed at 215 nm. For ITC **4d,** the CE was positive; consequently, the absolute configuration was *S*. In the case of ITC **4c**, the CE was negative; consequently, the absolute configuration was *R*. The presence of two different enantiomers was confirmed.

The absolute configurations of all chiral ITCs **4c**–**d** and **9a**–**i**, as well as their optical rotations, are presented in Table 3.

### 2.6. Proposed Mechanism of Synthesis of ITC Using DMT/NMM/TsO^−^

**Figure 9:**
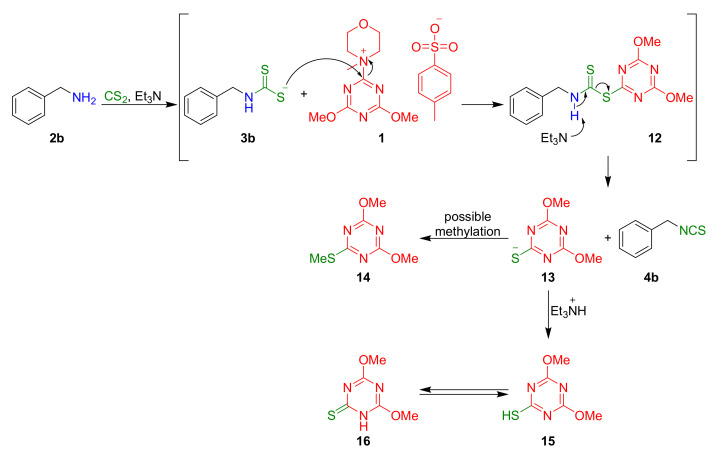
Possible mechanism for synthesis of isothiocyanates.

We postulate the following mechanism for the synthesis of ITCs using DMT/NMM/TsO^−^(Figure 9). Dithiocarbamate **3b** formed in a reaction amine **2b** with Et_3_N and CS_2_ reacts with DMT/NMM/TsO^−^(**1**), affording active ester **12**. Next, under the influence of Et_3_N, leaving group **13** is eliminated with the simultaneous formation of ITC **4b**. Forming the intermediate product which is a derivative of dithiocarbamate with desulfurating agent **12**, and then formation the final isothiocyanate in the presence a base is characteristic stage for synthesis of isothiocyanates using various desulfurating agents and it was described in many publication [57,60,64,66]. Next, the anion **13** can undergo two reactions. The first possible reaction is protonation of anion **13** by protonated triethylamine and formation if thiol **15** which is in equilibrium with its thiocarbonyl form **16**. However, due to the rapid tautomerism identification was impossible.

The second is possible methylation, leading to preparation of 2,4-dimethoxy-6-methylthio-1,3,5-triazine (**14**), which was isolated after flash chromatography with low yield and confirmed by ^1^H and ^13^C NMR. The yield of the isolated compound **14** was low, indicating a slight share this reaction.

### 2.7. Comparison DMT/NMM/TsO^−^ vs. Selected Desulfurating Agents

In order to show the superiority of DMT/NMM/TsO^−^ relative to other desulfurating agents the effectiveness of reagents was compared in the synthesis of model benzyl isothiocyanate (**4b**). All reactions were carried out in the same optimal microwave conditions (3 min, 90 °C), using different, known desulfurating agents: cyanuric chloride (TCT) [61], iodine [57], di-*tert*-butyl dicarbonate (Boc_2_O) with catalityc amount of DMAP [62], propane phosphonic acid anhydride (T3P^®^) [66], tosyl chloride (TsCl) [55], ethyl chloroformate [59], and 30% hydrogen peroxide (H_2_O_2_) [77], and using as substrate benzyl amine (**2b**). The final product was isolated after flash chromatography. All experiments are presented in Table 4.

The above results show that effectiveness of all desulfurating agents in microwave-conditions is very good (yield of **4b** is between 92 and 75%); however, DMT/NMM/TsO^−^ turned out to be the best desulfurating agent (yields of **4b** 92%) (Table 4, Entry 1). The TCT as well as I_2_ were also very efficient reagents; however, the yields of ITC **4b** were slightly lower, respectively, 87% and 86% (Table 4, Entries 2 and 3). Application of Boc_2_O with DMAP, T3P^®^, and TsCl also caused the yields of **4b** to be satisfactory (84–81%) (Table 4, Entries 4–6). The lower yields below 80% obtained using ethyl chloroformate and 30% H_2_O_2_ (Table 4, Entries 7 and 8) show that these compounds were definitely less effective than DMT/NMM/TsO^−^. Although, some of the reagents (TsCl and iodine) are cheaper than DMT/NMM/TsO^−^, the DMT/NMM/TsO^−^ allows us to obtain the product with higher yield, which is undoubtedly its greatest advantage.

### 2.8. Antibacterial Activity

Isothiocyanates derivatives of natural **9a–j** and unnatural amino acids **11a–c** were evaluated for antibacterial activity on Gram positive bacteria strain *S. aureus* and Gram negative bacteria strain *E. coli*, using chloramphenicol as positive control. The antibacterial activity of ITCs **9a**–**j** and **11a**–**c** based on the well diffusion method on trypticase soy agar (TSA) is presented in Table 5, and on the pictures of Petri dishes in the (Supporting Material Figure S87). ITCs **9a**–**j** and **11a**–**c** at micromolar concentration (16–27 μM) in DMSO or mixture DMSO and water (4 mg/mL) [78,79] were used in the test. The inhibition zones were reported in millimeter (mm).

Based on results in Table 5 more of the tested ITCs exhibited antibacterial activity. For *E. coli* the most active ITCs were **9d** and **9e** (ϕ > 36 mm). Additionally, good activity characterized ITCs **9i** and **9j** (ϕ about 30 mm). Others ITCs **9a–c**, **9f–h**, **11a**–**11c** also had antibacterial activity, although slightly lower (ϕ < 25 mm). Additionally ITCs **9e**, **9j** and **11a** exhibited very high antibacterial activity (ϕ > 32 mm) against *S. aureus.* ITCs **9d**, **9f**, **9h**, and **9i** had weaker, but also good activity (30 > ϕ > 25 mm). The other ITCs **9a–c**, **9g**, **11b–c** showed moderate antibacterial activity (ϕ < 25 mm).

For all tested compounds, the most active for both bacterial strains was ITC **9e** derived from methyl ester of L-valine. Probably the short and branched alkyl chain of isopropyl moiety was responsible for the highest activity. Despite the satisfactory antibacterial activity of tested ITCs **9a**–**j** and **11a**–**c** (16–27 μM), the activity of chloramphenicol used as a positive control was definitely higher (0.062 μM). The study on the mechanism of antimicrobial action and toxicity will be continued.

## 3. Materials and Methods

### 3.1. General Information

NMR spectra were measured on a Bruker Avance II Plus (Bruker Corporation, Billerica, MA, USA) spectrometer (700 MHz for ^1^H NMR and 176 MHz for ^13^C NMR) in CDCl_3_ solution. ^1^H and ^13^C NMR spectra were referenced according to the residual peak of the solvent, based on literature data. Chemical shifts (*δ*) were reported in ppm and coupling constants (*J*) in Hz. ^13^C NMR spectra were proton-decoupled. A monomode microwave reactor (CEM Discover) (CEM, Matthews, NC, USA) equipped with an Intelli Vent pressure control system was used. The standard method was applied and the maximum pressure was set to 250 psi. The temperatures of the reaction mixtures were measured with an external infrared sensor. Flash chromatography was performed using a glass column packed with Baker silica gel (30–60 μm). For TLC, silica gel with a 254 nm indicator on Al foils (Sigma−Aldrich, St. Louis, MO, USA) was used. All reagents and solvents were purchased from Sigma-Aldrich (Poland) and used as obtained. Optical rotations were measured at 25 °C on a PolaAAr 3001 Polarimeter (A. KRÜSS Optronic GmbH, Hamburg, Germany) at λ = 589 nm. Optical rotations were reported as follows: [α]^D^_25_ (c = g/100 mL solvent). The enantiomeric ratio (*er*) of **4c** and **4d** was determined by chiral stationary phase HPLC, using a Daicel Chiralpak ID column (hexane) and a Daicel Chiralpak IC column for compounds **9a**–**b** (hexane/*i*-PrOH, 98:2), with a column temperature of 30 °C and a flow rate of 1.0 mL/min. Melting points were obtained using a Büchi SMP-20 apparatus. CD studies were performed using a Jasco J–1500 spectrometer Far-UV (ABLE JASCO Polska, Krakow, Poland) and a rectangular quartz cuvette (1 mm path length, Hellma, Müllheim, Germany). The samples were prepared in methanol at a concentration of 0.52–0.30 mg/mL. All studies were carried out at rt. CD spectra were measured in the range of 190–300 nm. The experimental parameters were as follows: data pitch, 5 nm; scanning mode, continuous; scanning speed, 100nm/min; bandwidth, 4 nm; integration time, 1 s. Mass spectrometry analysis was performed on a Bruker microOTOF-QIII (Bruker Corporation, Billerica, MA, USA) equipped with electrospray ionization mode and a time of flight detector (TOF). IR spectra were measured on an FT-IR Alpha Bruker (ATR) instrument in cm^−1^.

### 3.2. General Procedure and Characterization of Compounds ***4a–ag***

#### 3.2.1. General Procedure for Compounds **4a–j**—Method A

Amine **2a**–**j** (2 mmol, 1 equiv.), Et_3_N (0.84 mL, 6 mmol, 3 equiv. or 1.68 mL, 12 mmol, 6 equiv. for **2j**), and CS_2_ (0.36 mL, 6 mmol, 3 equiv. or 0.72 mL, 12 mmol, 6 equiv. for **2j**) were dissolved in dry DCM (3 mL or 5 mL for **2j**) in a 10 mL pressure vial, equipped with a magnetic bar, and stirred 5 min at rt. Next, DMT/NMM/TsO^−^ (**1**) (0.828 g, 2 mmol, 1 equiv. or 1.656 g, 4 mmol, 2 equiv. for **2j**) was added. The reaction was carried under MW conditions (standard mode, 3 min, 90 °C). The reaction mixture was diluted with DCM (50 mL) and washed with H_2_O (5 mL), 1 N HCl (2 × 5 mL), H_2_O (5 mL), then dried under anhydrous MgSO_4_. The crude products were purified by flash chromatography on silica gel (7–8 g) using hexane as an eluent. Pure isothiocyanates **4a**–**j** were isolated after careful evaporation of the solvent and removal of volatile residues under reduced pressure. All the synthesized isothiocyanates have been described in the literature.

*2-Isothiocyanatoethylbenzene* (**4a**). Colorless oil. Yield 92% (0.3 g, 1.84 mmol) after flash chromatography (hexane). ^1^H NMR (700 MHz, CDCl_3_): δ = 7.36–7.34 (m, 2 H, C*H*_Ar_), 7.30–7.27 (m, 1 H, C*H*_Ar_), 7.23–7.22 (m, 2 H, C*H*_Ar_), 3.73 (t, *J*_HH_ = 7.0 Hz, 2 H, C*H*_2_NCS), 3.00 (t, *J*_HH_ = 7.0 Hz, 2 H, C*H*_2_). ^13^C NMR (176 MHz, CDCl_3_): δ = 137.1 (s, *C*_Ar_), 131.0 (s, N*C*S), 128.9 (s, 2 × *C*_Ar_H), 128.8 (s, 2 × *C*_Ar_H), 127.3 (s, *C*_Ar_H), 46.5 (s, *C*H_2_NCS), 36.6 (s, *C*H_2_). The analytical data are in agreement with those reported previously in the literature [63].

*(Isothiocyanatomethyl)benzene* (**4b**). Colorless oil. Yield 92% (0.273 g, 1.83 mmol) after flash chromatography (hexane). ^1^H NMR (700 MHz, CDCl_3_): δ = 7.42–7.39 (m, 2 H, C*H*_Ar_), 7.37–7.34 (m, 1 H, C*H*_Ar_), 7.33–7.32 (m, 2 H, C*H*_Ar_), 4.71 (s, 2 H, C*H*_2_NCS). ^13^C NMR (176 MHz, CDCl_3_): δ = 134.3 (s, *C*_Ar_), 132.4 (s, N*C*S), 129.1 (s, 2 × *C*_Ar_H), 128.5 (s, *C*_Ar_H), 126.9 (s, 2 × *C*_Ar_H), 48.8 (s, *C*H_2_NCS). The analytical data are in agreement with those reported previously in the literature [63].

*(R)-1-isothiocyanatoethylbenzene* (**4c**). Colorless oil. Yield 94% (0.306 g, 1.87 mmol) after flash chromatography (hexane). The *er* was determined by HPLC using a Chiralpak ID column, (hexane); t*_major_* = 9.36 min, t*_minor_* = 7.95 min (>99: 1 *er*). [α]^D^_25_ −18.1 (c 1.0, CHCl_3_) (lit. [α]^D^_25_ −18.3 (c 1.0, CHCl_3_)). ^1^H NMR (700 MHz, CDCl_3_): δ = 7.41–7.38 (m, 2 H, C*H*_Ar_), 7.34–7.32 (m, 3 H, C*H*_Ar_), 4.92 (q, *J*_HH_ = 6.8 Hz, 1 H, C*H*NCS), 1.68 (d, *J*_HH_ = 6.8 Hz, 3 H, C*H*_3_). ^13^C NMR (176 MHz, CDCl_3_): δ = 140.3 (s, *C*_Ar_), 132.5 (s, N*C*S), 129.0 (s, 2 × *C*_Ar_H), 128.3 (s, *C*_Ar_H), 125.5 (s, 2 × *C*_Ar_H), 57.2 (s, *C*HNCS), 25.1 (s, *C*H_3_). The concentration of the sample for CD analysis was 0.51 mg/mL. The analytical data are in agreement with those reported previously in the literature [63].

*(S)-1-isothiocyanatoethylbenzene* (**4d**). Colorless oil. Yield 92% (0.3 g, 1.84 mmol) after flash chromatography (hexane). The *er* was determined by HPLC using a Chiralpak ID column (hexane); t*_major_* = 7.95 min, t*_minor_* = 9.36 min (>99: 1 *er*). [α]^D^_25_ +17.5 (c 1.0, CHCl_3_) (lit. [α]^D^_25_ +17.6 (c 1.0, CHCl_3_)). ^1^H NMR (700 MHz, CDCl_3_): δ = 7.41–7.38 (m, 2 H, C*H*_Ar_), 7.34–7.32 (m, 3 H, C*H*_Ar_), 4.92 (q, *J*_HH_ = 6.8 Hz, 1 H, C*H*NCS), 1.68 (d, *J*_HH_ = 6.8 Hz, 3 H, C*H*_3_). ^13^C NMR (176 MHz, CDCl_3_): δ = 140.3 (s, *C*_Ar_), 132.5 (s, N*C*S), 129.0 (s, 2 × *C*_Ar_H), 128.3 (s, *C*_Ar_H), 125.5 (s, 2 × *C*_Ar_H), 57.1 (s, *C*HNCS), 25.1 (s, *C*H_3_). The concentration of the sample for CD analysis was 0.5 mg/mL. The analytical data are in agreement with those reported previously in the literature [63].

*1-Isothiocyanatohexane* (**4e**). Colorless oil. Yield 88% (0.251 g, 1.76 mmol) after flash chromatography (hexane). ^1^H NMR (700 MHz, CDCl_3_): δ = 3.50 (t, *J*_HH_ = 6.7 Hz, 2 H, C*H*_2_NCS), 1.71–1.67 (m, 2 H, C*H*_2_), 1.43–1.39 (m, 2 H, C*H*_2_), 1.35–1.27 (m, 4 H, 2 × C*H*_2_), 0.90 (t, *J*_HH_ = 7.1 Hz, 3 H, C*H*_3_). ^13^C NMR (176 MHz, CDCl_3_): δ = 129.7 (s, N*C*S), 45.2 (s, *C*H_2_NCS), 31.1 (s, *C*H_2_), 30.1 (s, *C*H_2_), 26.3 (s, *C*H_2_), 22.6 (s, *C*H_2_), 14.0 (s, *C*H_3_). The analytical data are in agreement with those reported previously in the literature [63].

*2-Isothiocyanatobutane* (**4f**). Colorless oil. Yield 72% (0.166 g, 1.44 mmol) after flash chromatography (hexane). ^1^H NMR (700 MHz, CDCl_3_): δ = 3.33 (d, *J*_HH_ = 6.2 Hz, 2 H, C*H*_2_NCS), 1.99 (sept, *J*_HH_ = 6.5 Hz, 1 H, C*H*), 1.00 (t, *J*_HH_ = 6.7 Hz, 6 H, 2 × C*H*_3_). ^13^C NMR (176 MHz, CDCl_3_): δ = 129.8 (s, N*C*S), 52.5 (s, *C*H_2_NCS), 29.7 (s, *C*H), 19.8 (s, 2 × *C*H_3_). The analytical data are in agreement with those reported previously in the literature [63].

*2-Isothiocyanatooctane* (**4g**). Colorless oil. Yield 97% (0.330 g, 1.94 mmol) after flash chromatography (hexane). ^1^H NMR (700 MHz, CDCl_3_): δ = 3.77–3.72 (m, 1 H, C*H*NCS), 1.65–1.60 (m, 1 H, *H* from C*H*_2_), 1.57–1.52 (m, 1 H, *H* from C*H*_2_), 1.47–1.42 (m, 1 H, *H* from C*H*_2_), 1.39–1.33 (m, 1 H, *H* from C*H*_2_), 1.34 (d, *J*_HH_ = 6.5 Hz, 3 H, C*H*_3_CH), 1.32–1.25 (m, 6 H, 3 × C*H*_2_), 0.88 (t, *J*_HH_ = 7.1 Hz, 3 H, C*H*_3_). ^13^C NMR (176 MHz, CDCl_3_): δ = 129.9 (s, N*C*S), 54.2 (s, *C*HNCS), 37.7 (s, *C*H_2_), 31.7 (s, *C*H_2_), 28.8 (s, *C*H_3_CH), 26.1 (s, *C*H_2_), 22.6 (s, *C*H_2_), 21.9 (s, *C*H_2_), 14.1 (s, *C*H_3_). The analytical data are in agreement with those reported previously in the literature [63].

*3-Isothiocyanatopentane* (**4h**). Colorless oil. Yield 82% (0.211 g, 1.64 mmol) after flash chromatography (hexane). ^1^H NMR (700 MHz, CDCl_3_): δ = 3.53–3.49 (m, 1 H, C*H*NCS), 1.66–1.60 (m, 4 H, 2 × C*H*_2_), 1.01 (t, *J*_HH_ = 7.4 Hz, 6 H, 2 × C*H*_3_). ^13^C NMR (176 MHz, CDCl_3_): δ = 130.0 (s, N*C*S), 61.9 (s, *C*HNCS), 28.6 (s, 2 × *C*H_2_), 10.6 (s, 2 × *C*H_3_). The analytical data are in agreement with those reported previously in the literature [63].

*1-Isothiocyanatoadamantane* (**4i**). White solid, mp 167–169 °C (lit. 167–169 °C). Yield 83% (0.321 g, 1.66 mmol) after flash chromatography (hexane). ^1^H NMR (700 MHz, CDCl_3_): δ = 2.11 (s, 3 H, 3 × C*H*), 1.98 (d, *J*_HH_ = 2.7 Hz, 6 H, 3 × C*H*_2_), 1.64 (q, *J*_HH_ = 12.5 Hz, 6 H, 3 × C*H*_2_). ^13^C NMR (176 MHz, CDCl_3_): δ = 129.7 (s, N*C*S), 58.6 (s, *C*NCS), 43.9 (s, 3 × *C*H_2_), 35.7 (s, 3 × *C*H_2_), 29.4 (s, 3 × *C*H). The analytical data are in agreement with those reported previously in the literature [63].

*1,6-Diisothiocyanatohexane* (**4j**). Colorless oil. Yield 70% (0.280 g, 1.4 mmol) after flash chromatography (hexane). ^1^H NMR (700 MHz, CDCl_3_): δ = 3.52 (t, *J*_HH_ = 6.5 Hz, 4 H, 2 × C*H*_2_NCS), 1.73–1.69 (m, 4 H, 2 × C*H*_2_), 1.46–1.44 (m, 4 H, 2 × C*H*_2_). ^13^C NMR (176 MHz, CDCl_3_): δ = 130.2 (s, N*C*S), 45.0 (s, 2 × *C*H_2_NCS), 29.8 (s, 2 × *C*H_2_), 26.0 (s, 2 × *C*H_2_). The analytical data are in agreement with those reported previously in the literature [66].

#### 3.2.2. General Procedure for Compounds **4a**, **4c**, **4e**, **4g** and **4i**—Method B

Amine **2a**, **2c**, **2e**, **2g**, or **2i** (2 mmol, 1 equiv.), Et_3_N (0.84 mL, 6 mmol, 3 equiv.), and CS_2_ (0.36 mL, 6 mmol, 3 equiv.) were dissolved in H_2_O (3 mL) in a 10 mL pressure vial equipped with a magnetic bar, and stirred for 5 min at rt. Next, DMT/NMM/TsO^−^ (**1**) (1.077 g, 2.6 mmol, 1.3 equiv.) was added. The reaction was carried under MW conditions (standard mode, 3 min, 90 °C). The reaction mixture was extracted with DCM (3 × 15 mL). The combined organic layers were washed with 1 N HCl (2 × 5 mL) and H_2_O (5 mL), then dried under anhydrous MgSO_4_. The crude product was purified by flash chromatography on silica gel (7–8 g) using hexane as eluent. Pure isothiocyanates **4a**, **4c**, **4e**, **4g**, and **4i** were isolated after careful evaporation of the solvent and volatile residues under reduced pressure.

*2-Isothiocyanatoethylbenzene* (**4a**). Colorless oil. Yield 89% (0.290 g, 1.78 mmol) after flash chromatography (hexane).

*(R)-1-Isothiocyanatoethylbenzene* (**4c**). Colorless oil. Yield 88% (0.286 g, 1.76 mmol) after flash chromatography (hexane).

*1-Isothiocyanatohexane* (**4e**). Colorless oil. Yield 86% (0.222 g, 1.72 mmol) after flash chromatography (hexane).

*2-Isothiocyanatooctane* (**4g**). Colorless oil. Yield 96% (0.329 g, 1.92 mmol) after flash chromatography (hexane).

*1-Isothiocyanatoadamantane* (**4i**). White solid. Yield 72% (0.190 g, 1.48 mmol) after flash chromatography (hexane).

#### 3.2.3. General Procedure for Compounds **7a–j**

Amine **5a**–**j** (2 mmol, 1 equiv.), DBU (0.9 mL, 6 mmol, 3 equiv. or 1.8 mL, 12 mmol, 6 equiv. for **5j**), and CS_2_ (0.36 mL, 6 mmol, 3 equiv. or 0.72 mL, 12 mmol, 6 equiv. for **5j**) were dissolved in dry DCM (3 mL or 5 mL for **5j**) in a 10 mL pressure vial equipped with a magnetic bar, and stirred 5 min at rt. Next, DMT/NMM/TsO^−^ (**1**) (0.828 g, 2 mmol, 1 equiv. or 1.656 g, 4 moml, 2 equiv. for **5j**) was added. The reaction was carried out under MW conditions (standard mode, 3 min, 90 °C). The reaction mixture was diluted with DCM (50 mL) and washed with H_2_O (5 mL), 1 N HCl (2 × 5 mL), and H_2_O (5 mL), then dried under anhydrous MgSO_4_. The crude products were purified by flash chromatography on silica gel (7–8 g) using hexane as an eluent. Pure isothiocyanates **7a**–**j** were isolated after careful evaporation of the solvent and removal of volatile residues under reduced pressure. All the synthesized isothiocyanates have been described in the literature.

*Isothiocyanatobenzene* (**7a**). Colorless oil. Yield 71% (0.192 g, 1.42 mmol) after flash chromatography (hexane). ^1^H NMR (700 MHz, CDCl_3_): *δ* = 7.37–7.34 (m, 2 H, C*H*_Ar_), 7.29–7.27 (m, 1 H, C*H*_Ar_), 7.23–7.21 (m, 2 H, C*H*_Ar_). ^13^C NMR (176 MHz, CDCl_3_): *δ* = 135.5 (s, N*C*S), 131.4 (s, *C*_Ar_NCS), 129.6 (s, 2 × *C*_Ar_H), 127.4 (s, *C*_Ar_H), 125.8 (s, 2 × *C*_Ar_H). The analytical data are in agreement with those reported previously in the literature [63].

*1-Isothiocyanato-4-methylbenzene* (**7b**). Colorless oil. Yield 87% (0.260 g, 1.74 mmol) after flash chromatography (hexane). ^1^H NMR (700 MHz, CDCl_3_): *δ* = 7.14 (d, *J*_HH_ = 8.7 Hz, 2 H, C*H*_Ar_), 7.11 (d, *J*_HH_ = 8.4 Hz, 2 H, C*H*_Ar_), 2.35 (s, 3 H, C*H*_3_). ^13^C NMR (176 MHz, CDCl_3_): *δ* = 137.6 (s, *C*_Ar_OMe), 134.7 (s, N*C*S), 130.2 (s, 2 × *C*_Ar_H), 128.5 (s, *C*_Ar_NCS), 125.6 (s, 2 × *C*_Ar_H), 21.3 (s, *C*H_3_). The analytical data are in agreement with those reported previously in the literature [80].

*2-Isothiocyanato-1,3-dimethylbenzene* (**7c**). Colorless oil. Yield 92% (0.3 g, 1.84 mmol) after flash chromatography (hexane). ^1^H NMR (700 MHz, CDCl_3_): *δ* = 7.09–7.04 (m, 3 H, C*H*_Ar_), 2.38 (s, 6 H, 2 × C*H*_3_). ^13^C NMR (176 MHz, CDCl_3_): *δ* = 135.7 (s, N*C*S), 135.2 (s, 2 × *C*_Ar_CH3), 129.7 (s, *C*_Ar_NCS), 128.1 (s, 2 × *C*_Ar_H), 127.0 (s, *C*_Ar_H), 18.7 (s, 2 × *C*H_3_). The analytical data are in agreement with those reported previously in the literature [63].

*1-Isothiocyanato-4-methoxybenzene* (**7d**). Colorless oil. Yield 87% (0.287 g, 1.74 mmol) after flash chromatography (hexane). ^1^H NMR (700 MHz, CDCl_3_): *δ* = 7.16 (d, *J*_HH_ = 9.1 Hz, 2 H, C*H*_Ar_), 6.85 (d, *J*_HH_ = 9.1 Hz, 2 H, C*H*_Ar_), 3.80 (s, 3 H, OC*H*_3_). ^13^C NMR (176 MHz, CDCl_3_): *δ* = 158.7 (s, *C*_Ar_OCH_3_), 134.3 (s, N*C*S), 127.0 (s, 2 × *C*_Ar_H), 123.7 (s, *C*_Ar_NCS), 114.9 (s, 2 × *C*_Ar_H), 55.6 (s, *C*H_3_O). The analytical date are in agreement with those reported previously in the literature [63].

*1-Isothiocyanato-3-methoxybenzene* (**7e**). Colorless oil. Yield 66% (0.218 g, 1.32 mmol) after flash chromatography (hexane). ^1^H NMR (700 MHz, CDCl_3_): *δ* = 7.23 (t, *J*_HH_ = 8.3 Hz, 1 H, C*H*_A_r), 6.83–6.81 (m, 2 H, C*H*_Ar_), 6.73 (t, *J*_HH_ = 2.2 Hz, 1 H, C*H*_Ar_), 3.79 (s, 3 H, C*H*_3_O). ^13^C NMR (176 MHz, CDCl_3_): *δ* = 160.4 (s, *C*_Ar_OMe), 135.5 (s, N*C*S), 132.2 (s, *C*_Ar_NCS), 130.3 (s, *C*_Ar_H), 118.2 (s, *C*_Ar_H), 113.7 (s, *C*_Ar_H), 111.2 (s, *C*_Ar_H), 55.6 (s, *C*H_3_O). The analytical data are in agreement with those reported previously in the literature [63].

*1-Isothiocyanato-3,5-dimethoxybenzene* (**7f**). White solid, mp 44–45 °C (lit. 48–49 °C). Yield 67% (0.262 g, 1.34 mmol) after flash chromatography (hexane). ^1^H NMR (700 MHz, CDCl_3_): *δ* = 6.38–6.36 (m, 3 H, C*H*_Ar_), 3.77 (s, 6 H, 2 × C*H*_3_O). ^13^C NMR (176 MHz, CDCl_3_): *δ* = 161.3 (s, 2 × *C*_Ar_OMe), 135.5 (s, N*C*S), 132.6 (s, *C*_Ar_NCS), 104.1 (s, 2 × *C*_Ar_H), 100.4 (s, *C*_Ar_H), 55.6 (s, 2 × *C*H_3_O). The analytical data are in agreement with those reported previously in the literature [81].

*1-Chloro-4-isothiocyanatobenzene* (**7g**). White solid, mp 42–43 °C (lit. 44–45 °C). Yield 61% (0.207 g, 1.22 mmol) after flash chromatography (hexane). ^1^H NMR (700 MHz, CDCl_3_): *δ* = 7.32 (d, *J*_HH_ = 8.9 Hz, 2 H, C*H*_Ar_), 7.15 (d, *J*_HH_ = 8.9 Hz, 2 H, C*H*_Ar_). ^13^C NMR (176 MHz, CDCl_3_): *δ* = 137.0 (s, N*C*S), 133.1 (s, *C*_Ar_), 130.2 (s, *C*_Ar_), 129.9 (s, 2 × *C*_Ar_H), 127.1 (s, 2 × *C*_Ar_H). The analytical data are in agreement with those reported previously in the literature [63].

*1-Fluoro-4-isothiocyanatobenzene* (**7h**). Colorless oil. Yield 59% (0.180 g, 1.18 mmol) after flash chromatography (hexane). ^1^H NMR (700 MHz, CDCl_3_): *δ* = 7.21–7.18 (m, 2 H, C*H*_Ar_), 7.05–7.02 (m, 2 H, C*H*_Ar_). ^13^C NMR (176 MHz, CDCl_3_): *δ* = 161.2 (d, *J*_CF_ = 248.8 Hz, *C*_Ar_F), 136.2 (s, N*C*S), 127.5 (s, *C*_Ar_NCS), 127.4 (d, *J*_CF_ = 8.3 Hz, *C*_Ar_H), 116.7 (d, *J*_CF_ = 23.9 Hz, *C*_Ar_H). The analytical data are in agreement with those reported previously in the literature [63].

*1-Bromo-4-isothiocyanatobenzene* (**7i**). White solid, mp 55–56 °C (lit. 59–60 °C). Yield 53% (0.227 g, 1.06 mmol) after flash chromatography (hexane). ^1^H NMR (700 MHz, CDCl_3_): *δ* = 7.47 (d, *J*_HH_ = 8.9 Hz, 2 H, C*H*_Ar_), 7.09 (d, *J*_HH_ = 8.8 Hz, 2 H, C*H*_Ar_). ^13^C NMR (176 MHz, CDCl_3_): *δ* = 137.2 (s, N*C*S), 132.9 (s, 2 × *C*_Ar_H), 130.7 (s, *C*_Ar_NCS), 127.3 (s, 2 × *C*_Ar_H), 120.9 (s, *C*_Ar_Br). The analytical data are in agreement with those reported previously in the literature [63].

*1,3-Diisothiocyanatobenzene* (**7j**). White solid, mp 49–50 °C (lit. 50–51 °C). Yield 40% (0.154 g, 0.8 mmol) after flash chromatography (hexane). ^1^H NMR (700 MHz, CDCl_3_): *δ* = 7.33 (t, *J*_HH_ = 8.1 Hz, 1 H, C*H*_Ar_), 7.12 (dd, *J*_HH_ = 8.1 Hz, *J*_HH_ = 2.0 Hz, 2 H, C*H*_Ar_), 7.06 (t, *J*_HH_ = 2.0 Hz, 1 H, C*H*_Ar_). ^13^C NMR (176 MHz, CDCl_3_): *δ* = 138.1 (s, 2 × N*C*S), 133.0 (s, 2 × *C*_Ar_NCS), 130.8 (s, *C*_Ar_H), 124.6 (s, 2 × *C*_Ar_H), 122.8 (s, *C*_Ar_H). The analytical data are in agreement with those reported previously in the literature [63].

#### 3.2.4. General Procedure for Compounds **9a–j**

Hydrochloride **8a**–**j** (2 mmol, 1 equiv.), NMM (0.66 mL, 6 mmol, 3 equiv. or 1.32 mL, 12 mmol, 6 equiv. for **8i**) and CS_2_ (0.36 mL, 6 mmol, 3 equiv. or 0.72 mL, 12 mmol, 6 equiv. for **8i**) were dissolved in dry DCM (5 mL) in 10 mL round bottom flask equipped with a magnetic bar, and stirred 10 min at rt. Next, DMT/NMM/TsO^-^ (**1**) (0.828 g, 2 mmol, 1 equiv. or 1.656 g, 4 mmol, 2 equiv. for **8i**) was added. The reaction was mixed 30 min at rt. After that the reaction mixture was diluted with DCM (50 mL) and washed by H_2_O (5 mL), 1 N HCl (2 × 5 mL), H_2_O (5 mL), and dried under anhydrous MgSO_4_. The crude products were purified by flash chromatography on silica gel (7–8 g) using mixture hexane: EtOAc 20: 1 as eluent. Pure isothiocyanates **9a**–**j** were isolated after careful evaporation of the solvent and removal of volatile residues under reduced pressure.

*(S)-Methyl2-Isothiocyanatopropanoate* (**9a**). Colorless oil. Yield 50% (0.145 g, 1.0 mmol) after flash chromatography (hexane/EtOAc 20:1). The *er* was determined by HPLC using a Chiralpak IC column (hexane/*i*-PrOH, 98:2); t*_major_* = 6.92 min, t*_minor_* = 6.81 min (>99: 1 *er*). ^1^H NMR (700 MHz, CDCl_3_): δ = 4.35 (q, *J*_HH_ = 7.1 Hz, 1 H, C*H*NCS), 3.81 (s, 3 H, C*H*_3_O), 1.60 (d, *J*_HH_ = 7.1 Hz, 3 H, C*H*_3_). ^13^C NMR (176 MHz, CDCl_3_): δ = 169.5 (s, *C*O), 137.5 (s, N*C*S), 54.9 (s, *C*H_3_O), 53.3 (s, *C*HNCS), 19.6 (s, *C*H_3_). IR (ATR): 2041 (NCS), 1744 (CO), 1450, 1435, 1289, 1208, 1149, 1053 cm^−1^. [α]^D^_25_ +24.1 (c 0.32, CHCl_3_) (lit. [α]^D^_25_ +25.8 (c 0.32, CHCl_3_)). HRMS: 145.0197, ([M]^+^, C_5_H_7_NO_2_S^+^; calc. 145.0204). The concentration of sample for CD analysis was 0.52 mg/mL (MeOH). The analytical data are in agreement with those reported previously in the literature [66].

*(R)-Methyl 2-Isothiocyanatopropanoate* (**9b**). Colorless oil. Yield 51% (0.147 g, 1.02 mmol) after flash chromatography (hexane/EtOAc 20:1). The *er* was determined by HPLC using a Chiralpak IC column (hexane/*i*-PrOH, 98:2); t*_major_* = 6.81 min, t*_minor_* = 6.92 min (>99: 1 *er*). ^1^H NMR (700 MHz, CDCl_3_): δ = 4.35 (q, *J*_HH_ = 7.1 Hz, 1 H, C*H*NCS), 3.81 (s, 3 H, C*H*_3_O), 1.60 (d, *J*_HH_ = 7.1 Hz, 3 H, C*H*_3_). ^13^C NMR (176 MHz, CDCl_3_): δ = 169.5 (s, *C*O), 137.5 (s, N*C*S), 54.9 (s, *C*H_3_O), 53.3 (s, *C*HNCS), 19.6 (s, *C*H_3_). IR (ATR): 2045 (NCS), 1746 (CO), 1450, 1435, 1289, 1209, 1150, 1054 cm^−1^. [α]^D^_25_−23.3 (c 0.32, CHCl_3_) (lit. [α]^D^_25_−22.8 (c 0.32, CHCl_3_)). HRMS: 145.0197, ([M]^+^, C_5_H_7_NO_2_S^+^; calc. 145.0196). The concentration of sample for CD analysis was 0.51 mg/mL (MeOH). The analytical data are in agreement with those reported previously in the literature [66].

*(S)-Benzyl 2-isothiocyanatopropanoate* (**9c**). Colorless oil. Yield 38% (0.168 g, 0.76 mmol) after flash chromatography (hexane/EtOAc 20:1). ^1^H NMR (700 MHz, CDCl_3_): δ = 7.40–7.34 (m, 5 H, C*H*_Ar_), 5.23 (d, *J*_HH_ = 1.5 Hz, 2 H, C*H*_2_), 4.37 (q, *J*_HH_ = 7.1 Hz, 1 H, C*H*NCS), 1.60 (t, *J*_HH_ = 7.1 Hz, 3 H, C*H*_3_). ^13^C NMR (176 MHz, CDCl_3_): δ = 168.9 (s, *C*O), 137.8 (s, N*C*S), 134.9 (s, *C*_Ar_), 128.8 (s, 3 C, 3 × *C*_Ar_H), 128.5 (s, 2 C, 2 × *C*_Ar_H), 68.1 (s, *C*H_2_), 55.0 (s, *C*HNCS), 19.6 (s, *C*H_3_). IR (ATR): 2049 (NCS), 1744 (CO), 1497, 1452, 1286, 1190, 1146, 1052, 741, 695 cm^−1^. [α]^D^_25_ +32.1 (0.31 CHCl_3_). HRMS: 221.0510, ([M]^+^, C_11_H_11_NO_2_S^+^; calc. 221.0501). The concentration of sample for CD analysis was 0.3 mg/mL (MeOH). The analytical data are in agreement with those reported previously in the literature [82].

*(S)-tert-butyl 2-isothiocyanatopropanoate* (**9d**). Colorless oil. Yield 35% (0.131 g, 0.7 mmol) after flash chromatography (hexane/EtOAc 20:1). ^1^H NMR (700 MHz, CDCl_3_): δ = 4.18 (q, *J*_HH_ = 7.1 Hz, 1 H C*H*NCS), 1.54 (d, *J*_HH_ = 7.1 Hz, 3 H, C*H*_3_), 1.50 (s, 9 H, (C*H*_3_)_3_). ^13^C NMR (176 MHz, CDCl_3_): δ = 168.0 (s, *C*O), 137.2 (s, N*C*S), 83.6 (s, *C*(CH_3_)_3_), 55.6 (s, *C*HNCS), 28.1 (s, 3 C, 3 × *C*H_3_), 19.5 (s, *C*H_3_). IR (ATR): 2053 (NCS), 1738 (CO), 1477, 1454, 1394, 1223, 1144, 1054, 866 cm^−1^. [α]^D^_25_ +21.4 (0.34 CHCl_3_). HRMS: 187.0667, ([M]^+^, C_8_H_13_NO_2_S^+^; calc. 187.0663). The concentration of sample for CD analysis was 0.37 mg/mL (MeOH). The analytical data are in agreement with those reported previously in the literature [83].

*(S)-Methyl 2-isothiocyanato-3-methylbutanoate* (**9e**). Colorless oil. Yield 63% (0.218 g, 1.26 mmol) after flash chromatography (hexane/EtOAc 20:1). ^1^H NMR (700 MHz, CDCl_3_): δ = 4.16 (d, *J*_HH_ = 4.2 Hz, 1 H, C*H*NCS), 3.79 (s, 3 H, C*H*_3_O), 2.35–2.28 (m, 1 H, C*H*), 1.06 (d, *J*_HH_ = 6.9 Hz, 3 H, C*H*_3_), 0.96 (d, *J*_HH_ = 6.8 Hz, 3 H, C*H*_3_). ^13^C NMR (176 MHz, CDCl_3_): δ = 168.6 (s, *C*O), 136.9 (s, N*C*S), 65.7 (s, *C*H_3_O), 53.0 (s, *C*HNCS), 32.7 (s, *C*H), 19.7 (s, *C*H_3_), 17.2 (s, *C*H_3_). IR (ATR): 2052 (NCS), 1746 (CO), 1436, 1392, 1258, 1205, 1181, 1146, 1062 cm^−1^.[α]^D^_25_ +16.3 (1.0 EtOH) (lit. [α]^D^_25_ +4.1 (1.0 EtOH). HRMS: 173.0510, ([M]^+^, C_7_H_11_NO_2_S^+^; calc. 173.0513). The concentration of sample for CD analysis was 0.46 mg/mL (MeOH). The analytical data are in agreement with those reported previously in the literature [56].

*(S)-Methyl 2-isothiocyanato-4-methylpentanoate* (**9f**). Colorless oil. Yield 57% (0.213 g, 1.14 mmol) after flash chromatography (hexane/EtOAc 20:1). ^1^H NMR (700 MHz, CDCl_3_): δ = 4.30 (2 × d, *J*_HH_ = 9.7 Hz, *J*_HH_ = 9.6 Hz, 1 H, C*H*NCS), 3.80 (s, 3 H, C*H*_3_O), 1.88–1.81 (m, 2 H, C*H*_2_), 1.73–1.69 (m, 1 H, C*H*(CH_3_)_2_), 0.97 (d, *J*_HH_ = 6.5 Hz, 3 H, C*H*_3_), 0.95 (d, *J*_HH_ = 6.5 Hz, 3 H, C*H*_3_). ^13^C NMR (176 MHz, CDCl_3_): δ = 169.4 (s, *C*O), 137.0 (s, N*C*S), 58.1 (s, *C*H_3_O), 53.2 (s, *C*HNCS), 42.5 (s, *C*H_2_), 25.2 (s, *C*H), 22.8 (s, *C*H_3_), 21.2 (s, *C*H_3_). IR (ATR): 2052 (NCS), 1748 (CO), 1437, 1270, 1229, 1203, 1149, 983 cm^−1^. [α]^D^_25_ −21.7 (0.32 CHCl_3_) (lit. [α]^D^_25_−18.0 (0.016 M CHCl_3_). HRMS: 187.0667, ([M]^+^, C_8_H_13_NO_2_S^+^; calc. 187.0661). The concentration of sample for CD analysis was 0.41 mg/mL (MeOH). The analytical data are in agreement with those reported previously in the literature [75].

*(2S)-Methyl 2-isothiocyanato-3-methylpentanoate* (**9g**). Colorless oil. Yield 46% (0.172 g, 0.92 mmol) after flash chromatography (hexane/EtOAc 20:1). ^1^H NMR (700 MHz, CDCl_3_): δ = 4.20 (d, *J*_HH_ = 4.5 Hz, 1 H, C*H*NCS), 3.81 (s, 3 H, C*H*_3_O), 2.11–2.06 (m, 1 H, C*H*CH_3_), 1.48–1.43 (m, 1 H, *H*_α_ from C*H*_2_), 1.34–1.27 (m, 1 H, *H*_β_ from C*H*_2_), 1.05 (d, *J*_HH_ = 6.8 Hz, 3 H, C*H*_3_CH), 0.92 (t, *J*_HH_ = 7.5 Hz, 3 H, C*H*_3_CH_2_). ^13^C NMR (176 MHz, CDCl_3_): δ = 168.6 (s, *C*O), 136.8 (s, N*C*S), 64.9 (s, *C*H_3_O), 53.0 (s, *C*HNCS), 39.1 (s, *C*H_2_), 24.6 (s, *C*H), 16.3 (s, *C*H_3_), 11.4 (s, *C*H_3_). IR (ATR): 2054 (NCS), 1746 (CO), 1457, 1436, 1248, 1204, 1144 cm^−1^. [α]^D^_25_ +23.5 (1.0 EtOH) (lit.^56^. [α]^D^_25_ +15.3 (1.0 EtOH). HRMS: 187.0667, ([M]^+^, C_8_H_13_NO_2_S^+^; calc. 187.0684). The concentration of sample for CD analysis was 0.52 mg/mL (MeOH). The analytical data are in agreement with those reported previously in the literature [56].

*(S)-Methyl 2-isothiocyanato-3-phenylpropanoate* (**9h**). Colorless oil. Yield 30% (0.132 g, 0.6 mmol) after flash chromatography (hexane/EtOAc 20:1). ^1^H NMR (700 MHz, CDCl3): δ = 7.36–7.34 (m, 2 H, C*H*_Ar_), 7.31–7.29 (m, 1 H, C*H*_Ar_), 7.23–7.22 (m, 2 H, C*H*_Ar_), 4.48 (dd, *J*_Hα__Hβ_ = 8.4 Hz, *J*_HαHγ_ = 4.8 Hz, 1 H, C*H*_α_NCS), 3.80 (s, 3 H, C*H*_3_O), 3.25 (dd, *J*_HγHβ_ = 13.8 Hz, *J*_HγHα_ = 4.7 Hz, 1 H, C*H*_γ_Ph), 3.13 (dd, *J*_HβHγ_ = 13.8 Hz, *J*_HβHα_ = 8.4 Hz, 1 H, C*H*_β_Ph). ^13^C NMR (176 MHz, CDCl_3_): δ = 168.4 (s, *C*O), 138.1 (s, N*C*S), 135.1 (s, *C*_Ar_), 129.4 (s, *C*_Ar_H), 128.9 (s, *C*_Ar_H), 127.7 (s, *C*_Ar_H), 60.9 (s, *C*H_3_O), 53.2 (s, *C*HNCS), 39.8 (s, *C*H_2_). IR (ATR): 2038 (NCS), 1745 (CO), 1436, 1271, 1208, 1175, 1117, 697 cm^−1^. [α]^D^_25_ –60.0 (c 1.0, toluene) (lit. [α]^D^_25_ –62.2 (c 1.0, toluene)). HRMS: 211.0510, ([M]^+^, C_11_H_11_NO_2_S^+^; calc. 221.0506). The concentration of sample for CD analysis was 0.41 mg/mL (MeOH). The analytical data are in agreement with those reported previously in the literature [66].

*(S)-Methyl 2,6-diisothiocyanatohexanoate* (**9i**). Colorless oil. Yield 25% (0.122 g, 0.5 mmol) after flash chromatography (hexane/EtOAc 20:1). ^1^H NMR (700 MHz, CDCl3): δ = 4.32 (2 × d, *J*_HH_ = 7.8 Hz, *J*_HH_ = 7.8 Hz, 1 H, C*H*NCS), 3.83 (s, 3 H, C*H*_3_O), 3.56 (t, *J*_HH_ = 6.5 Hz, 2 H, C*H*_2_NCS), 1.99–1.91 (m, 2 H, C*H*_2_), 1.78–1.73 (m, 2 H, C*H*_2_), 1.61–1.57 (m, 2 H, C*H*_2_). ^13^C NMR (176 MHz, CDCl_3_): δ = 168.7 (s, *C*O), 138.2 (s, N*C*SCH), 130.9 (s, N*C*SCH_2_), 59.2 (s, *C*H_3_O), 53.4 (s, *C*HNCS), 44.8 (s, *C*H_2_NCS), 32.7 (s, *C*H_2_), 29.3 (s, *C*H_2_), 22.8 (s, *C*H_2_). IR (ATR): 2167 (NCS), 2053 (NCS), 2036 (NCS), 1744 (CO), 1435, 1346, 1206, 1175 cm^−1^. [α]^D^_25_ −18.2 (0.5 toluene). HRMS: 244.0340, ([M]^+^, C_9_H_12_N_2_O_2_S_2_^+^; calc. 244.0343). The concentration of sample for CD analysis was 0.52 mg/mL (MeOH). New compound.

*Ethyl 2-isothiocyanatoacetate* (**9j**). Colorless oil. Yield 48% (0.139 g, 0.96 mmol) after flash chromatography (hexane/EtOAc 20:1). ^1^H NMR (700 MHz, CDCl3): δ = 4.27 (q, *J*_HH_ = 7.1 Hz, 2 H, C*H*_2_), 4.21 (s, 2 H, C*H*_2_NCS), 1.30 (t, *J*_HH_ = 7.1 Hz, 3 H, C*H*_3_). ^13^C NMR (176 MHz, CDCl_3_): δ = 166.2 (s, *C*O), 138.4 (s, N*C*S), 62.6 (s, *C*H_2_CH_3_), 46.5 (s, *C*HNCS), 14.1 (s, *C*H_3_). IR (ATR): 2052 (NCS), 1744 (CO), 1394, 1197, 1018 cm^−1^. HRMS: 145.0197, ([M]^+^, C_5_H_7_NO_2_S^+^; calc. 145.0189). The concentration of sample for CD analysis was 0.33 mg/mL (MeOH). The analytical data are in agreement with those reported previously in the literature [41].

#### 3.2.5. General Procedure for Compounds **11a–c**

Hydrochloride **10a**–**c** (2 mmol, 1 equiv.), Et_3_N (0.84 mL, 6 mmol, 3 equiv.) and CS_2_ (0.36 mL, 6 mmol, 3 equiv.) were dissolved in dry DCM (3 mL) in 10 mL pressure vial, equipped with a magnetic bar, and stirred 5 min at rt. Next, DMT/NMM/TsO^−^ (**1**) (0.828 g, 2 mmol, 1 equiv.) was added. The reaction was carried under MW conditions (standard mode, 3 min, 90 °C). After that, the reaction mixture was diluted with DCM (50 mL) and washed by H_2_O (5 mL), 1 N HCl (2 × 5 mL), H_2_O (5 mL), and dried under anhydrous MgSO_4_. The crude product were purified by flash chromatography on silica gel (7–8 g) using hexane as eluent. Pure isothiocyanates **11a**–**c** were isolated after evaporation of the solvent under reduced pressure.

*Ethyl 6-isothiocyanatohexanoate* (**11a**). Colorless oil. Yield 75% (0.301 g, 1.5 mmol) after flash chromatography (hexane/EtOAc 10:1). ^1^H NMR (700 MHz, CDCl3): δ = 4.12 (q, *J*_HH_ = 7.1 Hz, 2 H, C*H*_2_O), 3.51 (t, *J*_HH_ = 6.6 Hz, 2 H, C*H*_2_NCS), 2.31 (t, *J*_HH_ = 7.4 Hz, C*H*_2_CO), 1.73–1.79 (m, 2 H, C*H*_2_), 1.67–1.63 (m, 2 H, C*H*_2_), 1.47–1.42 ((m, 2 H, C*H*_2_), 1.25 (t, *J*_HH_ = 7.1 Hz, 3 H, C*H*_3_). ^13^C NMR (176 MHz, CDCl_3_): δ = 173.3 (s, *C*O), 130.1 (s, N*C*S), 60.4 (s, *C*H_2_O), 44.9 (s, *C*H_2_NCS), 34.1 (s, *C*H_2_), 29.7 (s, *C*H_2_), 26.1 (s, *C*H_2_), 24.2 (s, *C*H_2_), 14.3 (s, *C*H_3_). IR (ATR): 2175 (NCS), 2088 (NCS), 1728 (CO), 1452, 1180, 1155 cm^−1^. HRMS: 201.0823, ([M]^+^, C_9_H_15_NO_2_S^+^; calc. 201.0825). New compound.

*Ethyl 4-(isothiocyanatomethyl)benzoate* (**11b**). White solid, mp 45–46 °C. Yield 81% (0.358 g, 1.62 mmol) after flash chromatography (hexane/EtOAc 10:1). ^1^H NMR (700 MHz, CDCl3): δ = 8.06 (d, *J*_HH_ = 8.5 Hz, 2 H, C*H*_Ar_), 7.39 (d, *J*_HH_ = 8.6 Hz, 2 H, C*H*_Ar_), 4.78 (s, 2 H, C*H*_2_), 4.38 (q, *J*_HH_ = 7.1 Hz, 2 H, C*H*_2_O), 1.40 (t, *J*_HH_ = 7.2 Hz, 3 H, C*H*_3_). ^13^C NMR (176 MHz, CDCl_3_): δ = 166.0 (s, *C*O), 139.1 (s, *C*_Ar_), 133.6 (s, N*C*S), 130.7 (s, *C*_Ar_), 130.3 (s, 2 C, 2 × *C*_Ar_H), 126.7 (s, 2 C, 2 × *C*_Ar_H), 61.2 (s, *C*H_2_O), 48.5 (s, *C*H_2_NCS), 14.4 (s, *C*H_3_). IR (ATR): 2197 (NCS), 2118 (NCS), 1698 (CO), 1610, 1411, 1273, 1175, 1103, 1018, 746 cm^−1^. HRMS: 221.0510, ([M]^+^, C_11_H_11_NO_2_S^+^; calc. 221.0509). New compound.

*Trans-ethyl 4-(isothiocyanatomethyl)cyclohexanecarboxylate* (**11c**). Colorless oil. Yield 92% (0.418 g, 1.84 mmol) after flash chromatography (hexane/EtOAc 10:1). ^1^H NMR (700 MHz, CDCl3): δ = 4.11 (q, *J*_HH_ = 7.1 Hz, 2 H, C*H*_2_O), 3.38 (d, *J*_HH_ = 6.2 Hz, 2 H, C*H*_2_NCS), 2.23 (tt, *J*_HH_ = 12.3 Hz, *J*_HH_ = 3.6 Hz, 1 H, C*H*), 2.05 (dd, *J*_HH_ = 13.7 Hz, *J*_HH_ = 3.5 Hz, 2 H, C*H*_2_), 1.87 (dd, *J*_HH_ = 13.7 Hz, *J*_HH_ = 3.4 Hz, 2 H, C*H*_2_), 1.70–1.64 (m, 1 H, C*H*), 1.45 (qd, *J*_HH_ = 13.4 Hz, *J*_HH_ = 3.5 Hz, 2 H, C*H*_2_), 1.24 (t, *J*_HH_ = 7.1 Hz, 3 H, *C*H_3_), 1.08 (qd, *J*_HH_ = 13.2 Hz, *J*_HH_ = 3.5 Hz, 2 H, C*H*_2_). ^13^C NMR (176 MHz, CDCl_3_): δ = 175.5 (s, *C*O), 130.2 (s, N*C*S), 60.3 (s, *C*H_2_O), 50.9 (s, *C*H_2_NCS), 42.9 (s, *C*H), 37.9 (s, *C*H), 29.4 (s, 2 × *C*H_2_), 28.2 (s, 2 × *C*H_2_), 14.2 (s, *C*H_3_). IR (ATR): 2180 (NCS), 2090 (NCS), 1724 (CO), 1449, 1175, 1065, 1040, 681 cm^−1^. HRMS: 227.0980, ([M]^+^, C_11_H_17_NO_2_S^+^; calc. 227.0989). New compound.

#### 3.2.6. 2,4- dimethoxy-6-methylthio-1,3,5-triazine (**14**)

Yield 7% (0.027 g, 0.14 mmol) after flash chromatography (hexane/EtOAc 10:1). ^1^H NMR (700 MHz, CDCl_3_): δ = 4.00 (s, 6 H, 2 × OC*H*_3_), 2,53 (s, 3 H, SC*H_3_*). ^13^C NMR (176 MHz, CDCl_3_): δ = 185.5 (s, *C*S), 171.4 (s, 2 × *C*OCH_3_), 55.4 (s, 2 × O*C*H_3_), 13.5 (s, S*C*H_3_). The analytical data are in agreement with those reported previously in the literature [84].

### 3.3. Study of Antimicrobial Activity

Antibacterial activities of solutions of tested compounds were evaluated using well diffusion method on trypticase soy agar TSA (Merck). The inhibition zones were reported in millimeter (mm). The 24 h single colonies on agar plates were used to prepare the bacterial suspensions with the turbidity of 0.5 McFarland degree (~1.5 × 10^8^ CFU/mL). Turbidity of the bacterial suspensions were measured using a DEN−1 densitometer (Merck). *Staphylococcus aureus* (ATCC 6538) and *Escherichia coli* (ATCC 8739) were used as references for the antibacterial assay. TSA agar plates were inoculated with bacterial strain under aseptic conditions and wells (diameter = 12mm) were filled with 250 µL of the test samples and incubated at 37 °C for 24 h. After the incubation period, the diameter of the growth inhibition zones was measured.

The sterile distilled water was used as a negative control while chloramphenicol (Sigma-Aldrich) at the concentration (20 μg/mL) 0.062 μM in DMSO was used as a positive standard. All tests were performed in triplicate.

## 4. Conclusions

To conclude, 4-(4,6-dimethoxy-1,3,5-triazin-2-yl)-4-methylmorpholinium toluene-4-sulfonate (DMT/NMM/TsO^−^) can be recognized as a new and efficient desulfurating agent suitable for microwave-assisted, one-pot synthesis of aliphatic and aromatic isothiocyanates and classical synthesis (normal reaction). The synthetic procedures presented in this study enabled the rapid and simple acquisition of a library of 20 structurally diverse isothiocyanates with good or very good yields (40–97%), high purity, and in the case of optically active compounds, low racemization (er 99 > 1). The synthesis of aliphatic isothiocyanates also took place in aqueous medium in the presence of organic base, with only slightly lower yields compared to those obtained in DCM.

DMT/NMM/TsO^−^ was also used to synthesis of isothiocyanate derivatives from methyl, ethyl, benzyl, and *tert*-butyl esters of L- and D- natural amino acids (alanine, valine, leucine, isoleucine, phenylalanine, and lysine) as well as achiral glycine, with satisfactory yields (25–63%), high purity, and low racemization (er 99 > 1). Due to the observed racemization under microwave-assisted syntheses, these reactions were performed under normal conditions. The absolute configuration of chiral ITCs was determined by CD. The Cotton Effect was strongly positive for ITCs with the *S* configuration, and for ITCs with the *R* configuration it was strong negative. The optical rotation of all chiral ITCs was also measured. As well as isothiocyanate derivatives of natural amino acids, isothiocyanate derivatives of ethyl esters of 6-aminocaproic acid, 4-(aminomethyl)benzoic acid, and tranexamic acid were synthesized with DMT/NMM/TsO^−^ in a microwave reactor, rapidly and with very high yields (75–92%), and in the case of *trans* ITC **11c** without racemization.

Antibacterial activity against *E. coli* and *S. aureus* bacterial strains showed that the most of tested isothiocyanates derivatives of natural and unnatural amino acids are active; however, their activity was worse than activity of positive control–chloramphenicol. The most active for both strains was ITC **9e**. Further research is ongoing to determine the biological properties of ITCs derived from both natural and unnatural amino acids.

## Figures and Tables

**Table 1:** Optimization of conditions of synthesis of isothiocyanate **4a**, derived from model amine **2a** ^a^.

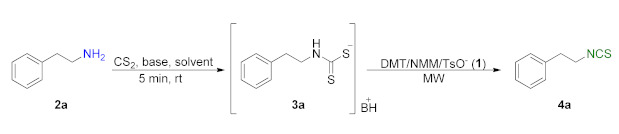						
Entry	MW Conditions		Base (Equiv.)	Solvent	DMT/NMM/TsO^−^ (Equiv.)	Yield (%) ^b^
	Time (min)	Temp (°C)				
1 ^c^	3	90	Et_3_N (4)	DCM	1.0	90
2	3	90	NMM (4)	DCM	1.0	78
3	3	90	DBU (4)	DCM	1.0	69
4	3	90	-	DCM	1.0	51
5	3	90	Et_3_N (3)	DCM	1.0	92
6 ^d^	3	90	Et_3_N (3)	DCM	1.3	92
7	3	90	Et_3_N (3)	DCM	0.7	86
8	3	90	Et_3_N (3)	DCM	0	55
9	3	90	Et_3_N (3)	H_2_O	1.0	73
10 ^e^	3	90	Et_3_N (3)	H_2_O	1.3	89
11 ^f,g,h^	30	rt	Et_3_N (3)	DCM	1.0	82

^a^—reagents and conditions: 10 mL pressure vial, **2a** (0.25 mL, 2 mmol, 1 equiv.), base, CS_2_ (0.36 mL, 6 mmol, 3 equiv.), solvent (3 mL). First step: 5 min, rt., then DMT/NMM/TsO^−^ (**1**). Time and temperature of microwave (MW) irradiation are presented in Table 1. Standard mode (initial power 200 W); ^b^—yield of **4a** after flash chromatography (hexane 100%); ^c^—elongation time to 5 and 10 min resulted the same yield (90%); ^d^—increasing the amount of **1** to 1.6 equiv. resulted in the same yield (92%); ^e^—increasing the amount of **1** to 1.6 equiv. resulted in the same yield (89%); ^f^—normal reaction at rt.; ^g^—prolonging the time to 60 min resulted in the same yield (82%), shortening the time to 10 min resulted in a lower yield (79%); ^h^—reaction in sealed tube (30 min, 90 °C) resulted in the same yield (82%).

**Table 2:** Optimization of reaction conditions of synthesis of isothiocyanate **7a**, derived from model amine **5a** ^a^.

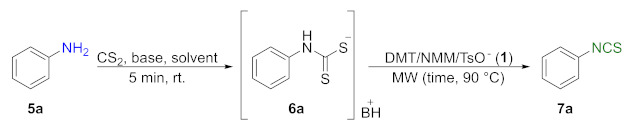					
Entry	MW Conditions	Base (Equiv.)	Solvent	DMT/NMM/TsO^−^ (Equiv.)	Yield (%) ^b^
	Time (min)				
1	3	Et_3_N (3)	DCM	1.0	30
2	3	DBU (3)	DCM	1.0	71
3	3	DBU (4)	DCM	1.0	67
4	3	DBU (4)	DCM	1.3	70
5	10	DBU (3)	DCM	1.0	67
6	3	DBU (3)	H_2_O	1.3	21

^a^–reagents and conditions: 10 mL pressure vial, **5a** (0.19 mL, 2 mmol, 1 equiv.), base, CS_2_ (0.36 mL, 6 mmol, 3 equiv.), solvent (3 mL). First step: 5 min, rt., then DMT/NMM/TsO^−^ (**1**), time of microwave irradiation see Table 2, temp. = 90 °C, standard mode (initial power 200 W); ^b^–yield of **7a** after flash chromatography (hexane 100%).

**Table 3:** Absolute configurations and optical rotations for ITCs **4c**–**d** and **9a**–**i**.

Entry	Compound	Absolute Configuration	[α]_D_ (Concentration)
1	**4c**	R	−18.1 (1.0 CHCl_3_)
2	**4d**	S	+17.5 (1.0 CHCl_3_)
3	**9a**	S	+24.1 (0.32 CHCl_3_)
4	**9b**	R	−23.3 (0.32 CHCl_3_)
5	**9c**	S	+32.1 (0.31 CHCl_3_)
6	**9d**	S	+21.4 (0.34 CHCl_3_)
7	**9e**	S	+16.3 (1.0 EtOH)
8	**9f**	S	−21.7 (0.32 CHCl_3_)
9	**9g**	S	+23.5 (1.0 EtOH)
10	**9h**	S	−60.0 (1.0 toluene)
11	**9i**	S	−18.2 (0.5 CHCl_3_)

**Table 4:** Comparison of the efficiency of DMT/NMM/TsO^−^ with selected desulfurating agents in synthesis benzyl isothiocyanate (**4b**) ^a^.

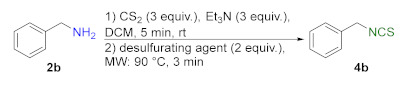		
Entry	DesulfuratingAgents	Yields (%)
1 ^b^	DMT/NMM/TsO^−^	92
2	TCT	87
3	I_2_	86
4	Boc_2_O, DMAP_cat._	84
5	T3P^®^	83
6	TsCl	81
7	Ethyl chloroformate	78
8	H_2_O_2_ 30%	75

^a^-conditions: hydrochloride **2b** (2 mmol), Et_3_N (0.83 mL, 3 equiv., 6 mmol), CS_2_ (0.36 mL, 3 equiv., 6 mmol), DCM (3 mL); first step 5 min, rt, then desulfurating agent (1 equiv., 2 mmol), MW irradiation in standard mode 3 min, 90 °C. Yield of products after flash chromatography (hexane 100%). ^b^-this work.

**Table 5:** Antibacterial activity of isothiocyanates **9a–j** and **11a–c**.

Entry	Compound (Concentration)	Structure	Growth Inhibition of *E. coli* (Mean ± DS)	Growth Inhibition of *S. aureus* (Mean ± DS)
1	**9a** (4 mg/mL, 27 μM)	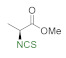	15.3 ± 0.3	16.1 ± 0.4
2	**9b** (4 mg/mL, 27 μM)	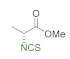	22.0 ± 0.4	22.7 ± 0.3
3	**9c** (4 mg/mL, 18 μM)	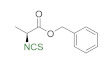	22.1 ± 0.1	18.4 ± 0.6
4	**9d** (4 mg/mL, 21 μM)	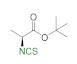	36.0 ± 0.7	25.1 ± 0.3
5	**9e** (4 mg/mL, 23 μM)	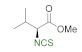	36.2 ± 0.2	32.3 ± 0.1
6	**9f** (4 mg/mL, 21 μM)	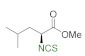	18.3 ± 0.3	25.2 ± 0.3
7	**9g** (4 mg/mL, 21 μM)	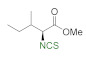	20.2 ± 0.2	17.5 ± 0.3
8	**9h** (4 mg/mL, 18 μM)	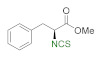	20.1 ± 0.3	24.5 ± 0.6
9	**9i** (4 mg/mL, 16 μM)	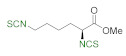	29.3 ± 0.1	28.3 ± 0.2
10	**9j** (4 mg/mL, 27 μM)	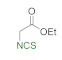	30.0 ± 0.4	32.1 ± 0.1
11	**11a** (4 mg/mL, 20 μM)	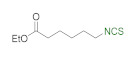	20.1 ± 0.3	32.3 ± 0.3
12	**11b** (4 mg/mL, 18 μM)	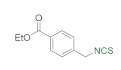	24.4 ± 0.6	16.3 ± 0.4
13	**11c** (4 mg/mL, 17 μM)	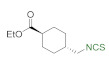	20.8 ± 0.2	15.5 ± 0.2
15	**Negative control**	sterile water	NO	NO
14	**Positive control** (20 μg/mL, 0.062 μM)	Chloramphenicol	36.2 ± 0.2	38.8 ± 0.3

## Data Availability

Data is contained within the article.

## Supplementary Materials

The following are available online. Figures S1–S68: ^1^H and ^13^C NMR spectra of compounds **4a**–**j**, **7a**–**j**, **9a**–**j, 11a**–**c**, and **14**; Figures S69–S74: HPLC chromatograms of compounds **4c**–**d** and **9a**–**b**; Figures S75–S86: CD spectra of compounds **4c**–**d** and **9a**–**j**; Figure S87: Pictures of Petri dishes, tests for antibacterial activity against *Staphylococcus aureus* (ATCC 6538) and *Escherichia coli* (ATCC 8739).

## Sample Availability

Samples of the compounds **4a**–**j**, **7a**–**j**, **9a**–**j, 11a**–**c**, and **14** are available from the authors.
