# Supplementary material for: Synthesis of Isothiocyanates Using DMT/NMM/TsO− as a New Desulfurization Reagent
Source: Molecules. 2021 May 6;26(9):2740. doi: 10.3390/molecules26092740 (PMC8125326; doi:10.3390/molecules26092740)
Supplement: Supplementary file 1 [file molecules-26-02740-s001.zip › molecules-1157630-supplementary.pdf]

Synthesis of isothiocyanates using DMT/NMM/TsO⁻ as a new desulfurization reagent

Łukasz Janczewski[1]*, Dorota Kręgiel[2] and Beata Kolesińska[1]

[1]Institute of Organic Chemistry, Faculty of Chemistry, Lodz University of Technology, Zeromskiego 116, 90-924 Lodz, Poland
[2]Department of Environmental Biotechnology, Faculty of Biotechnology and Food Sciences, Lodz University of Technology, Wolczanska 171/173, 90-924 Lodz, Poland
*Correspondence: lukasz.janczewski@p.lodz.pl

Table of contents

1. **Copies of ^{1}H and ^{13}C NMR spectra 5**
2. **Copies of HPLC chromatograms 40**
3. **Copies of CD spectra 43**
4. **Pictures of Petri dishes of antibacterial tests 46**

Table of contents

Fig S1. ^{1}H NMR of compound 4a. 5

Fig S2. ^{13}C NMR of compound 4a 5

Fig S3. ^{1}H NMR of compound 4b. 6

Fig S4. ^{13}C NMR of compound 4b. 6

Fig S5. ^{1}H NMR of compound 4c..... 7

Fig S6. ^{13}C NMR of compound 4c..... 7

Fig S7. ^{1}H NMR of compound 4d 8

Fig S8. ^{13}C NMR of compound 4d 8

Fig S9. ^{1}H NMR of compound 4e 9

Fig S10. ^{13}C NMR of compound 4e 9

Fig S11. ^{1}H NMR of compound 4f 10

Fig S12. ^{13}C NMR of compound 4f 10

Fig S13. ^{1}H NMR of compound 4g 11

Fig S14. ^{13}C NMR of compound 4g 11

Fig S15. ^{1}H NMR of compound 4h 12

Fig S16. ^{13}C NMR of compound 4h 12

Fig S17. ^{1}H NMR of compound 4i 13

Fig S18. ^{13}C NMR of compound 4i 13

Fig S19. ^{1}H NMR of compound 4j 14

Fig S20. ^{13}C NMR of compound 4j 14

Fig S21. ^{1}H NMR of compound 7a 15

Fig S22. ^{13}C NMR of compound 7a 15

Fig S23. ^{1}H NMR of compound 7b 16

Fig S24. ^{13}C NMR of compound 7b 16

Fig S25. ^{1}H NMR of compound 7c..... 17

Fig S26. ^{13}C NMR of compound 7c..... 17

Fig S27. ^{1}H NMR of compound 7d 18

Fig S28. ^{13}C NMR of compound 7d 18

Fig S29. ^{1}H NMR of compound 7e 19

Fig S30. ^{13}C NMR of compound 7e 19

Fig S31. ^{1}H NMR of compound 7f 20

Fig S32. ^{13}C NMR of compound 7f 20

Fig S33. ^{1}H NMR of compound 7g 21

Fig S34. ^{13}C NMR of compound 7g 21

Fig S35. ^{1}H NMR of compound 7h 22
Fig S36. ^{13}C NMR of compound 7h 22
Fig S37. ^{1}H NMR of compound 7i 23
Fig S38. ^{13}C NMR of compound 7i 23
Fig S39. ^{1}H NMR of compound 7j 24
Fig S40. ^{13}C NMR of compound 7j 24
Fig S41. ^{1}H NMR of compound 9a 25
Fig S42. ^{13}C NMR of compound 9a 25
Fig S43. ^{1}H NMR of compound 9b 26
Fig S44. ^{13}C NMR of compound 9b 26
Fig S45. ^{1}H NMR of compound 9c........ 27
Fig S46. ^{13}C NMR of compound 9c........ 27
Fig S47. ^{1}H NMR of compound 9d 28
Fig S48. ^{13}C NMR of compound 9d 28
Fig S49. ^{1}H NMR of compound 9e 29
Fig S50. ^{13}C NMR of compound 9e 29
Fig S51. ^{1}H NMR of compound 9f 30
Fig S52. ^{13}C NMR of compound 9f 30
Fig S53. ^{1}H NMR of compound 9g 31
Fig S54. ^{13}C NMR of compound 9g 31
Fig S55. ^{1}H NMR of compound 9h 32
Fig S56. ^{13}C NMR of compound 9h 32
Fig S57. ^{1}H NMR of compound 9i 33
Fig S58. ^{13}C NMR of compound 9i 33
Fig S59. ^{1}H NMR of compound 9j 34
Fig S60. ^{13}C NMR of compound 9j 34
Fig S61. ^{1}H NMR of compound 11a 35
Fig S62. ^{13}C NMR of compound 11a 35
Fig S63. ^{1}H NMR of compound 11b 36
Fig S64. ^{13}C NMR of compound 11b 36
Fig S65. ^{1}H NMR of compound 11c........ 37
Fig S66. ^{13}C NMR of compound 11c........ 37
Fig S67. ^{1}H NMR of compound 14 38
Fig S68. ^{13}C NMR of compound 14 38
Fig S69. HPLC chromatogram of compound 4c 39

Fig S70. HPLC chromatogram of compound 4d 39
Fig S71. Chiral HPLC chromatogram of mixture of compounds 4c and 4d 40
Fig S72. HPLC chromatogram of compound 9a 40
Fig S73. HPLC chromatogram of compound 9b 41
Fig S74. HPLC chiral chromatogram of mixture of compounds 9a and 9b 41
Fig S75. CD spectrum of ITC 9a. 42
Fig S76. CD spectrum of ITC 9b. 42
Fig S77. CD spectrum of ITC 9c. 42
Fig S78. CD spectrum of ITC 9d. 43
Fig S79. CD spectrum of ITC 9e. 43
Fig S80. CD spectrum of ITC 9f. 43
Fig S81. CD spectrum of ITC 9g. 44
Fig S82. CD spectrum of ITC 9h. 44
Fig S83. CD spectrum of ITC 9i. 44
Fig S84. CD spectrum of ITC 9j. 45
Fig S85. CD spectrum of ITC 4c 45
Fig S86. CD spectrum of ITC 4d. 45
Fig S87. Pictures of petri dishes, tests for antibacterial activity against *Staphylococcus aureus* (ATCC 6538) and *Escherichia coli* (ATCC 8739). 46

1. Copies of ^{1}H and ^{13}C NMR spectra

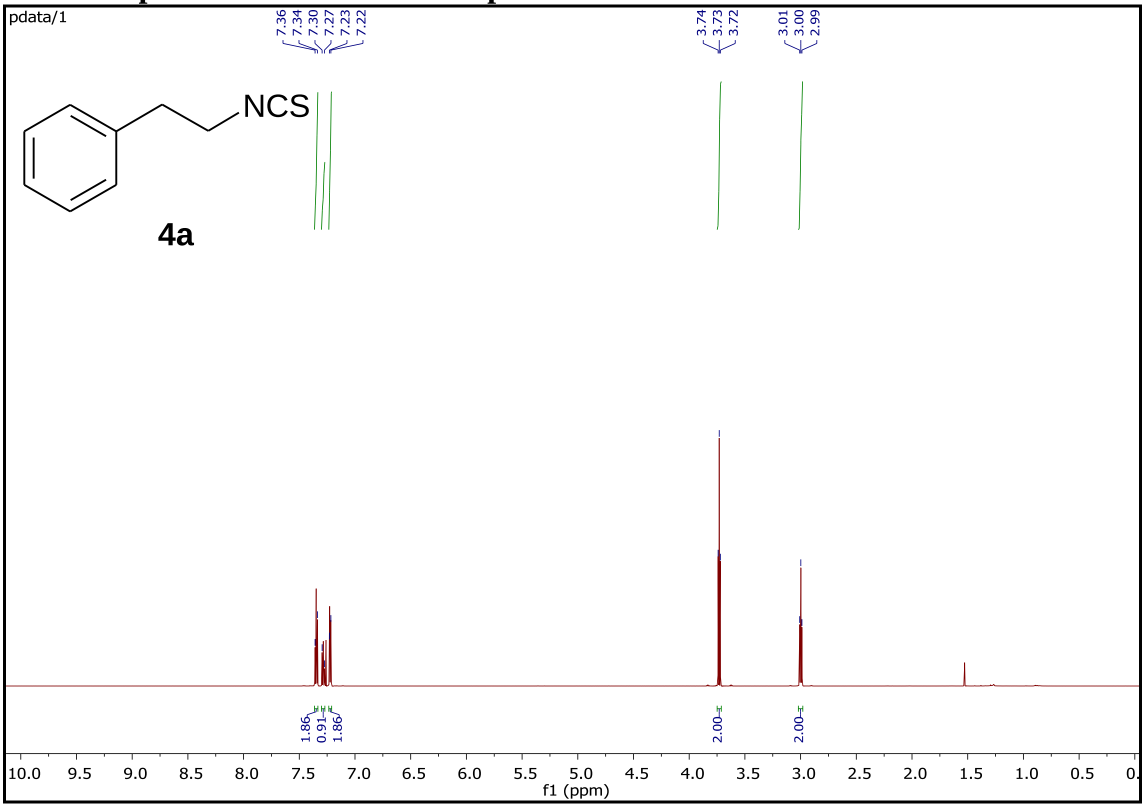

Fig S1. ^{1}H NMR of compound **4a.**

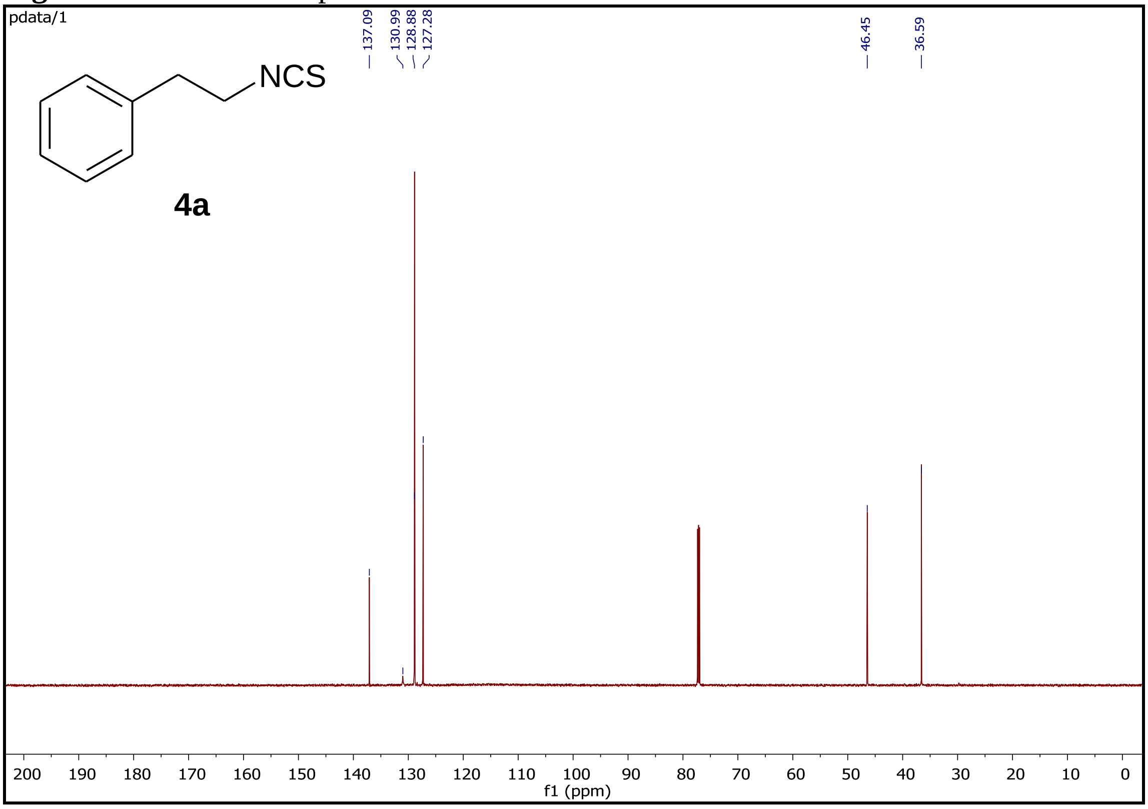

Fig S2. ^{13}C NMR of compound **4a**

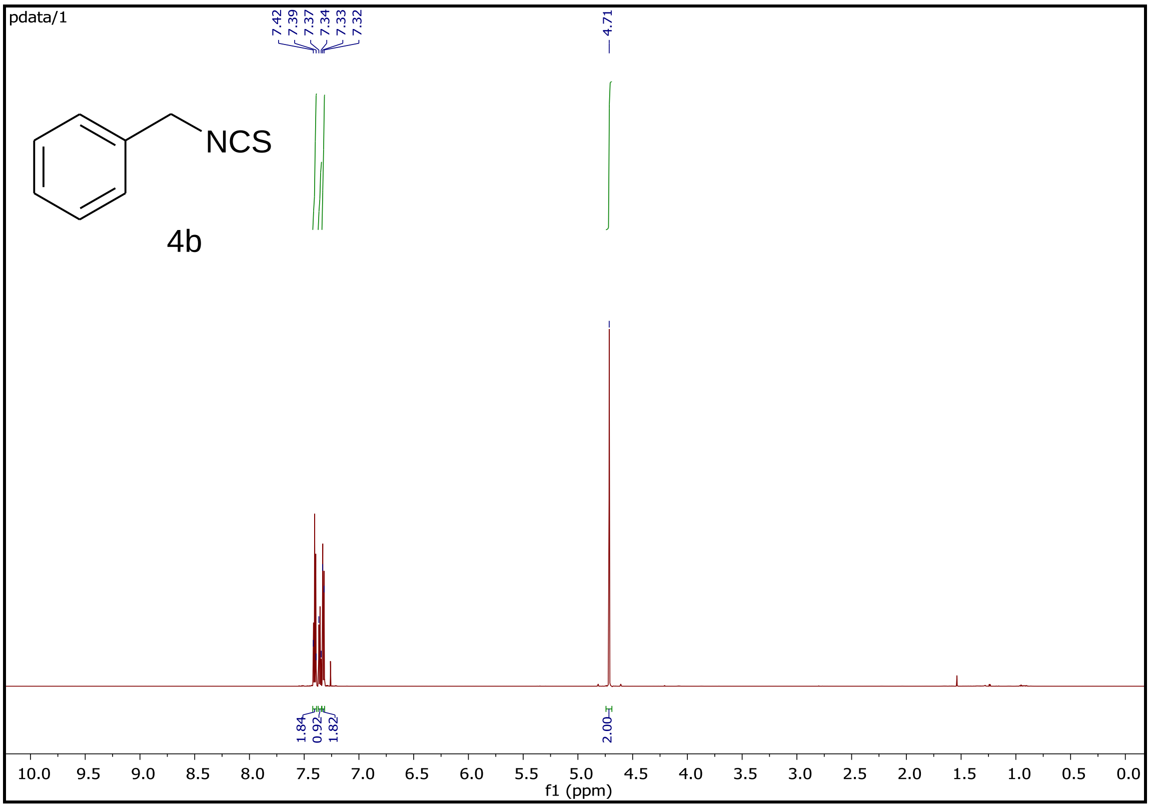

Fig S3. ^{1}H NMR of compound **4b**.

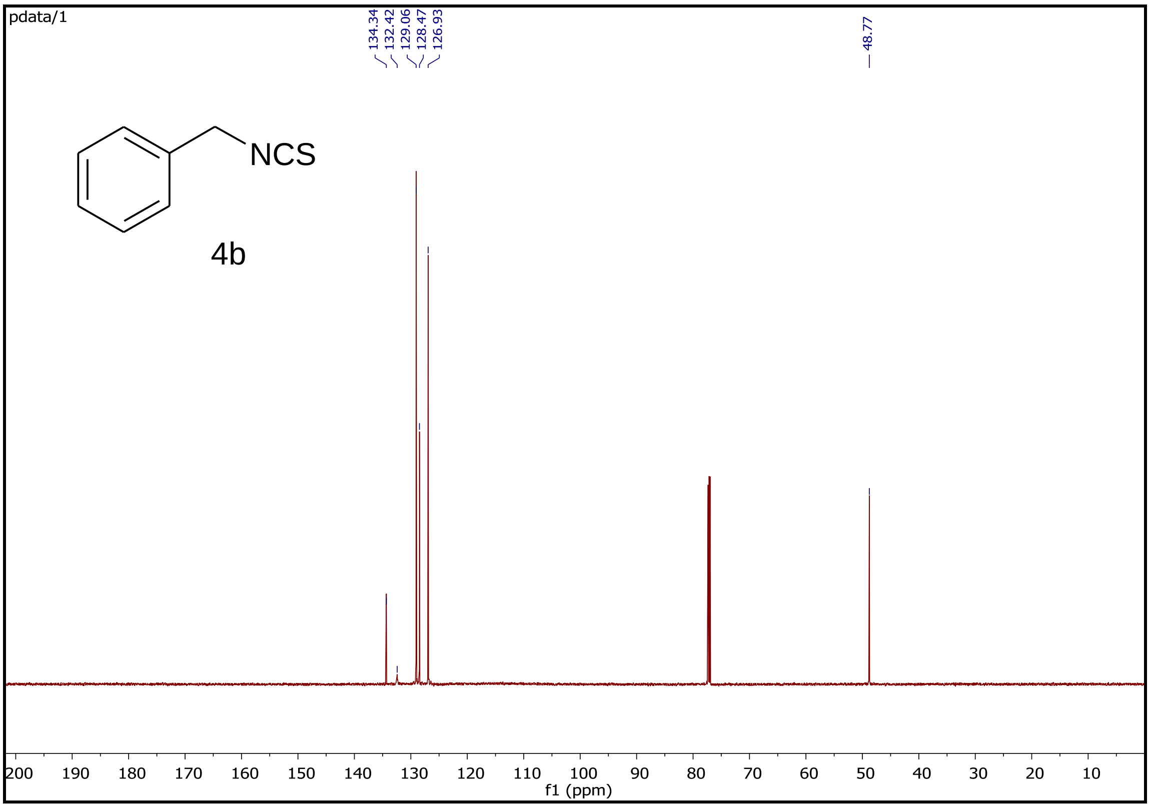

Fig S4. ^{13}C NMR of compound **4b**.

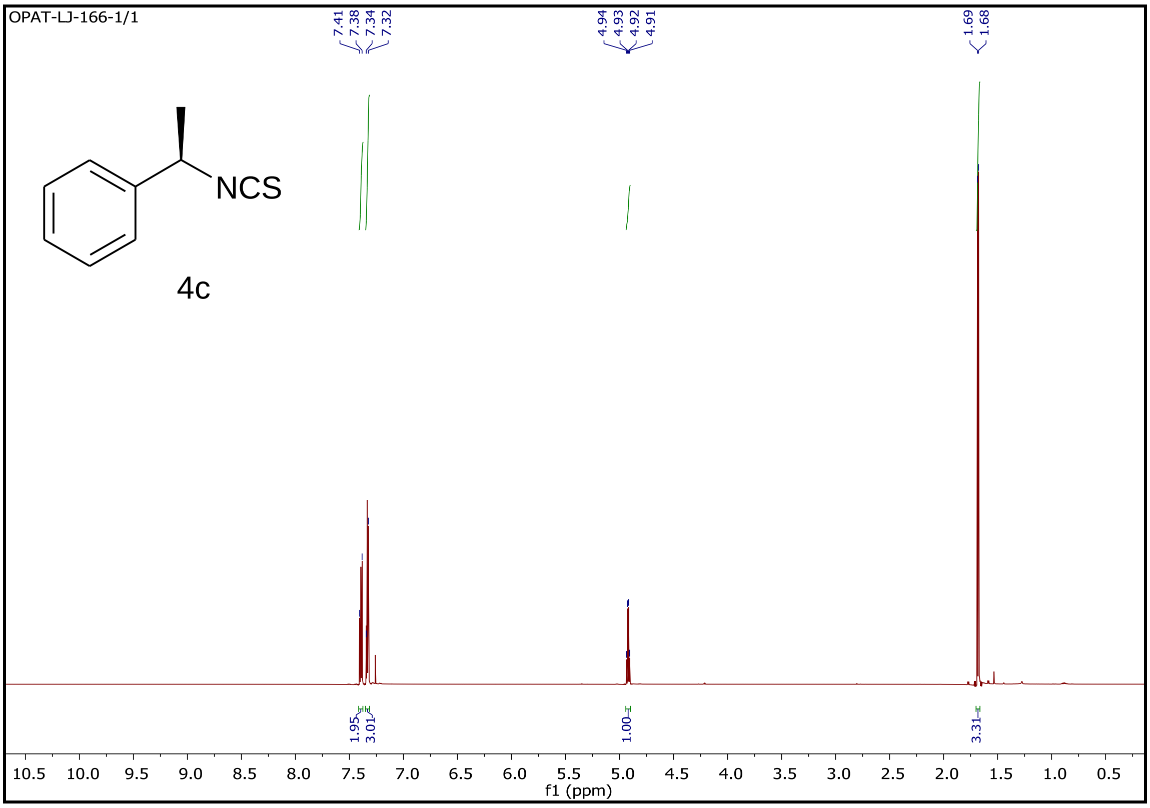

Fig S5. ^{1}H NMR of compound **4c**

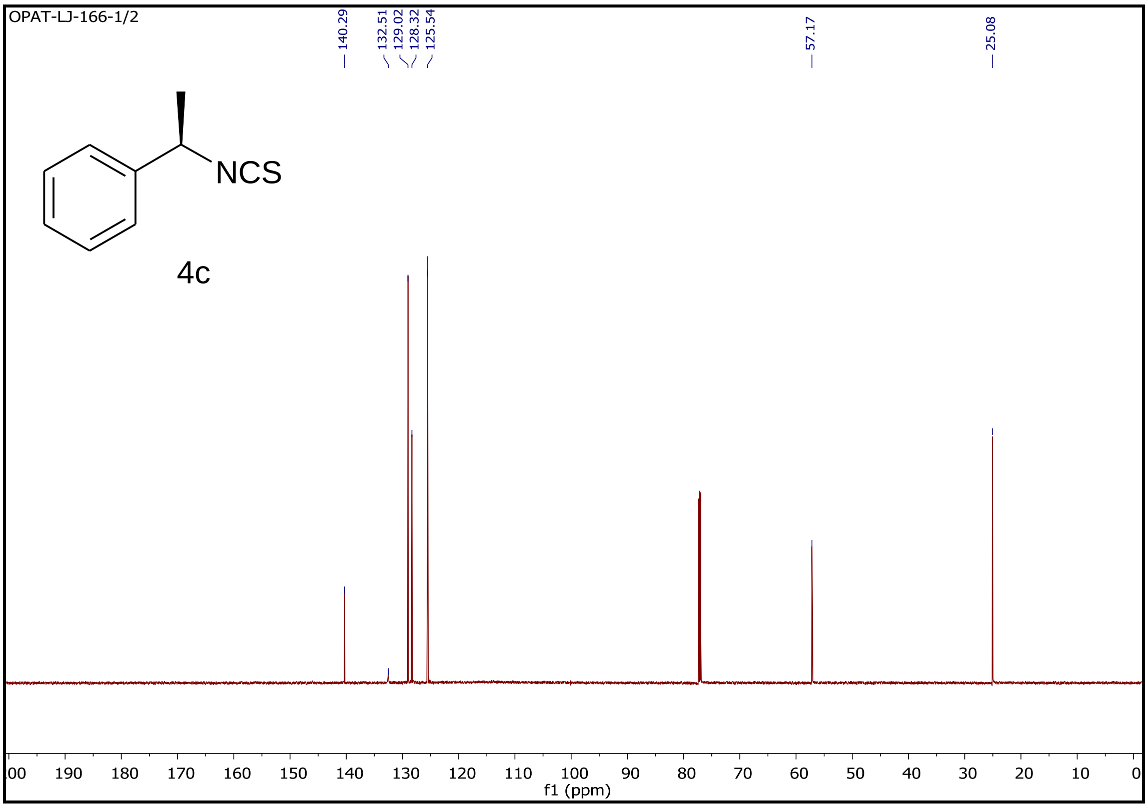

Fig S6. ^{13}C NMR of compound **4c**

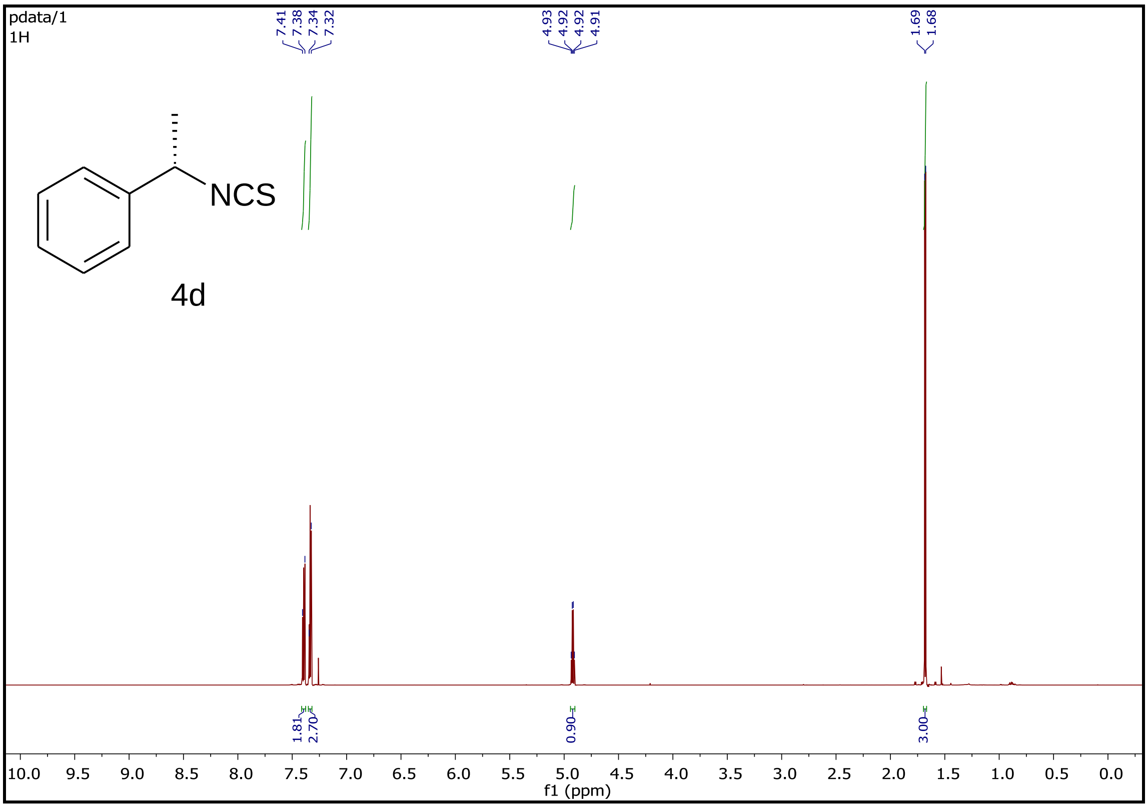

Fig S7. ^{1}H NMR of compound **4d**

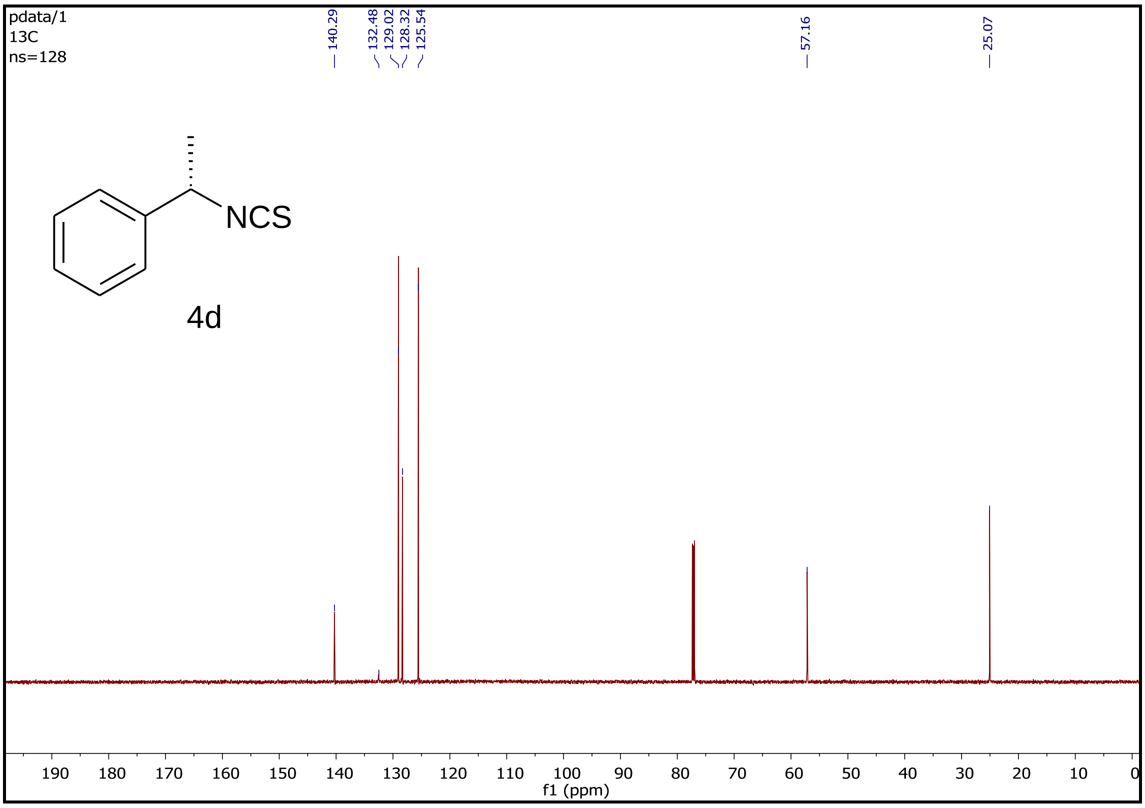

Fig S8. ^{13}C NMR of compound **4d**

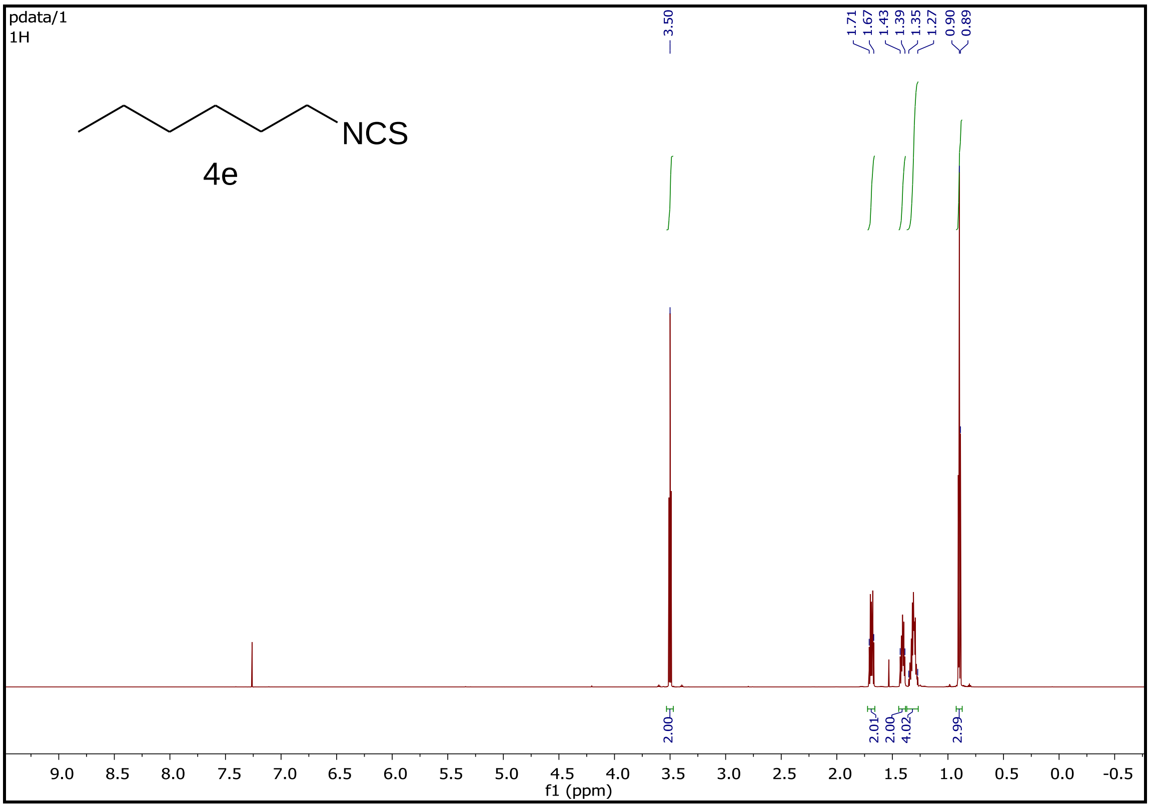

Fig S9. ^{1}H NMR of compound **4e**

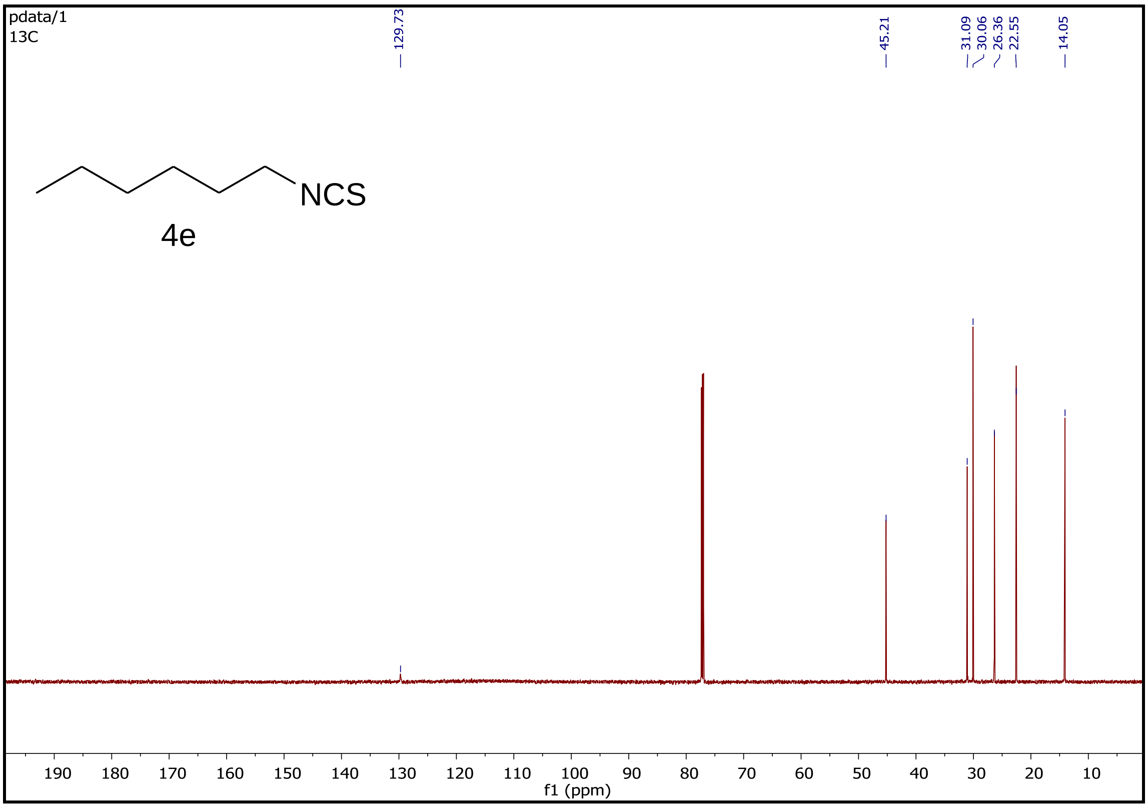

Fig S10. ^{13}C NMR of compound **4e**

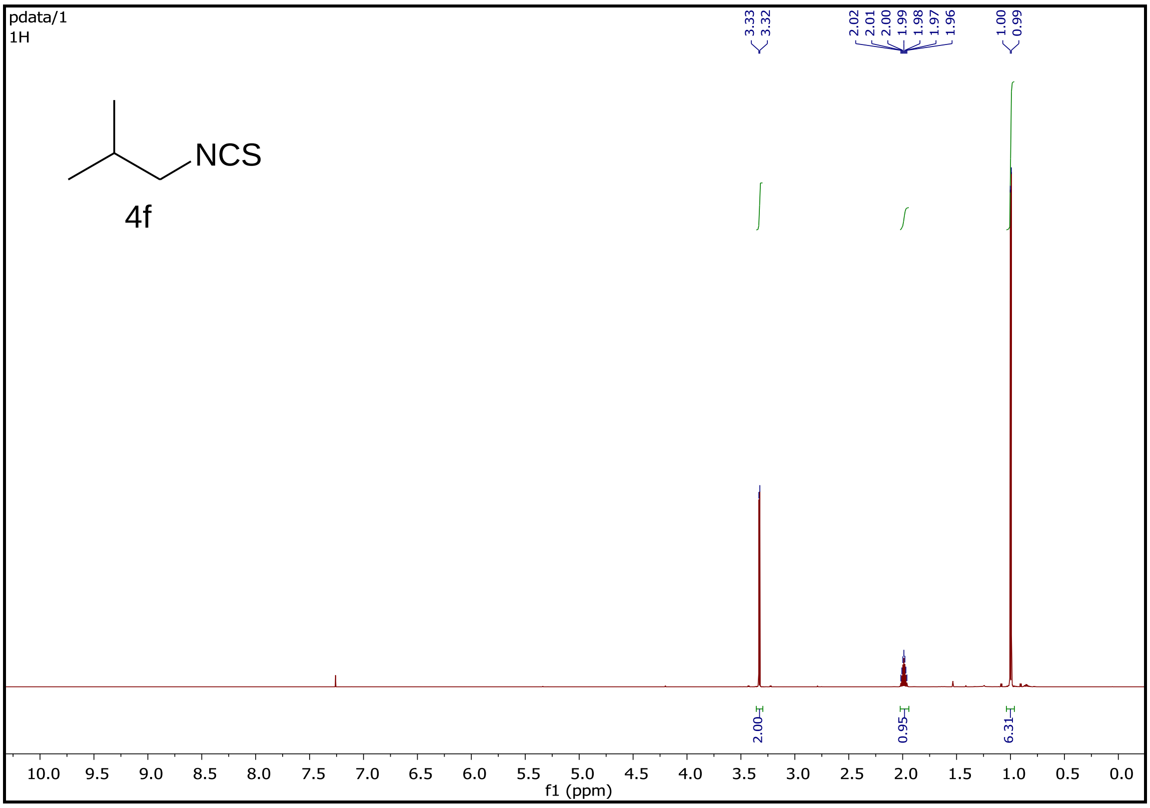

Fig S11. ^{1}H NMR of compound **4f**

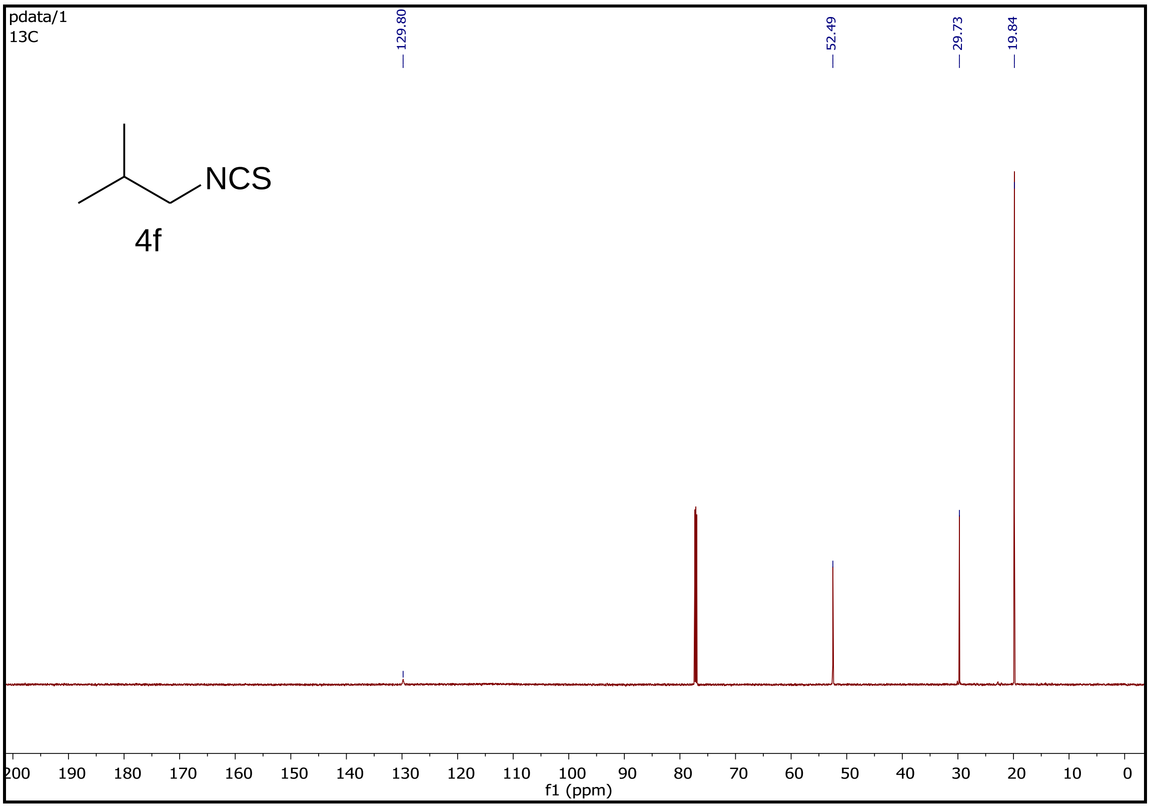

Fig S12. ^{13}C NMR of compound **4f**

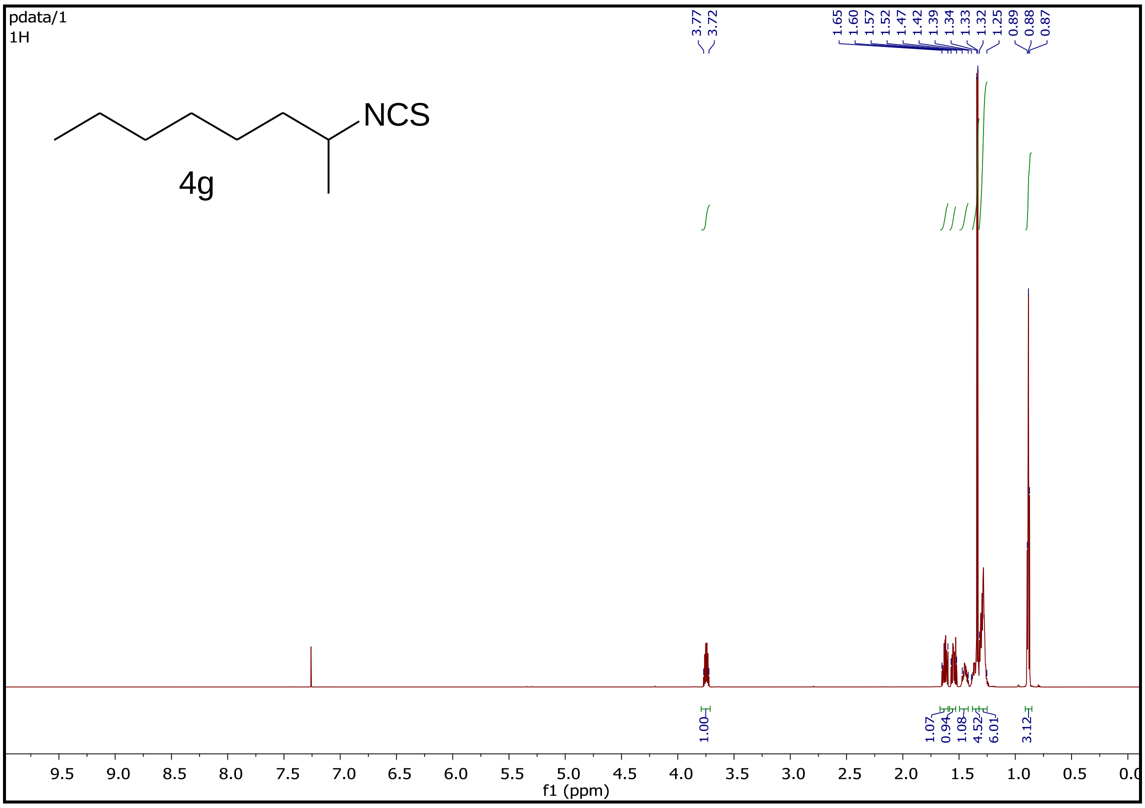

Fig S13. ^{1}H NMR of compound **4g**

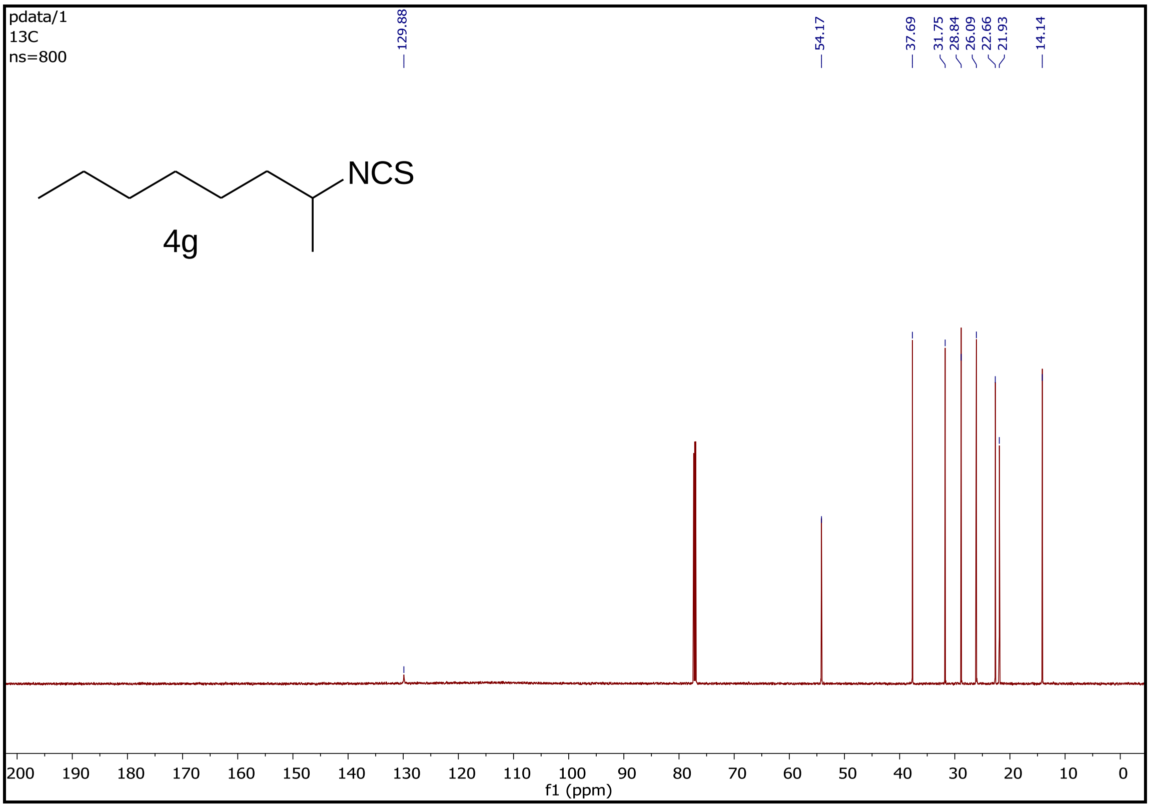

Fig S14. ^{13}C NMR of compound **4g**

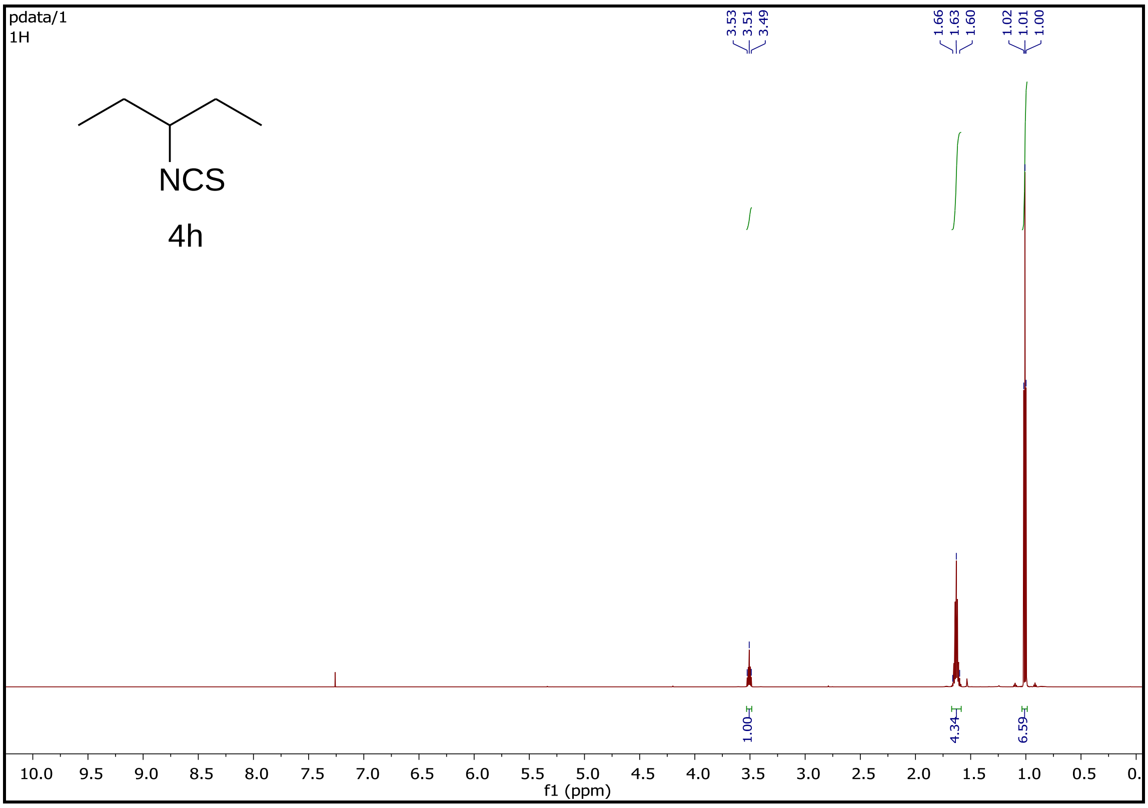

Fig S15. ^{1}H NMR of compound **4h**

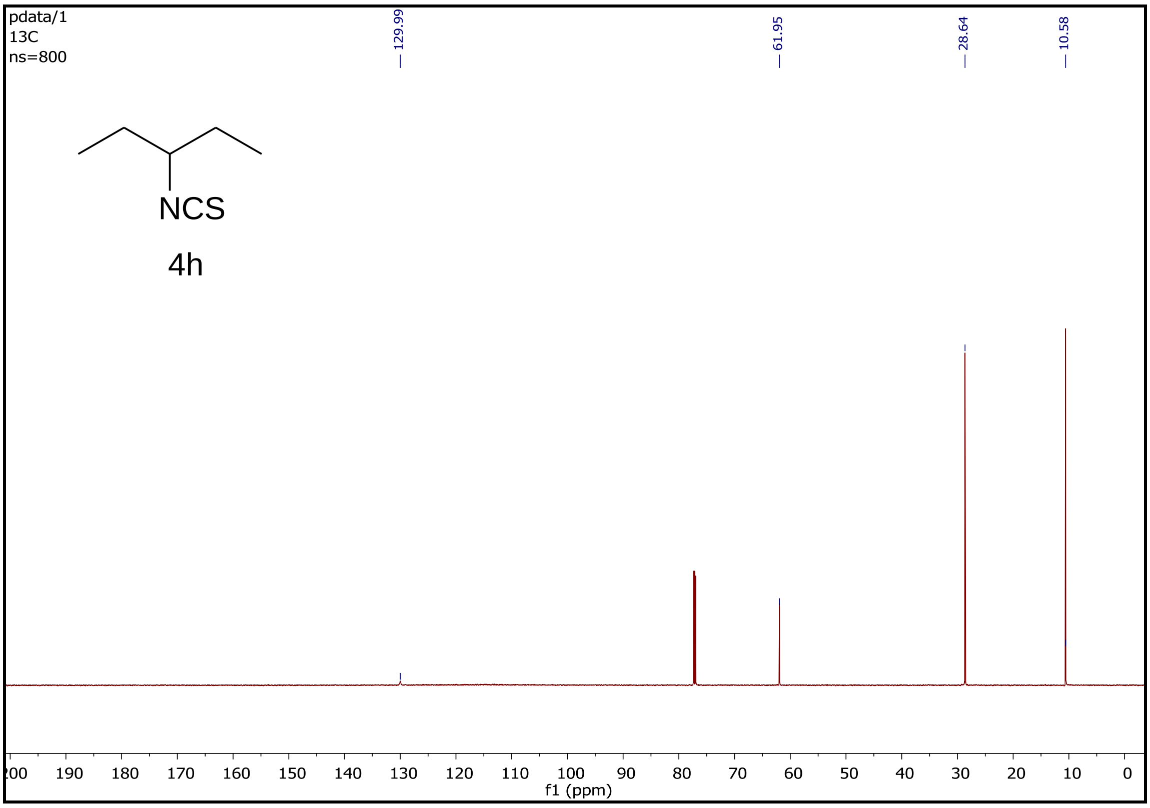

Fig S16. ^{13}C NMR of compound **4h**

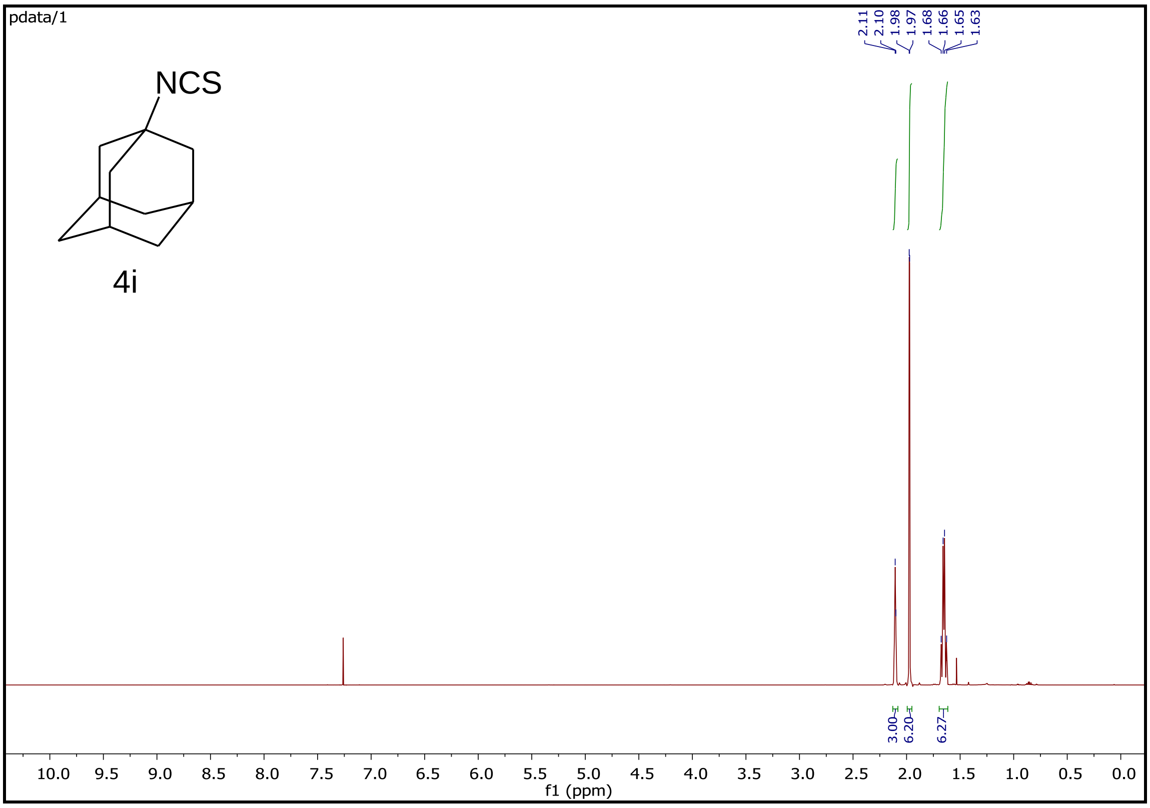

Fig S17. ^{1}H NMR of compound **4i**

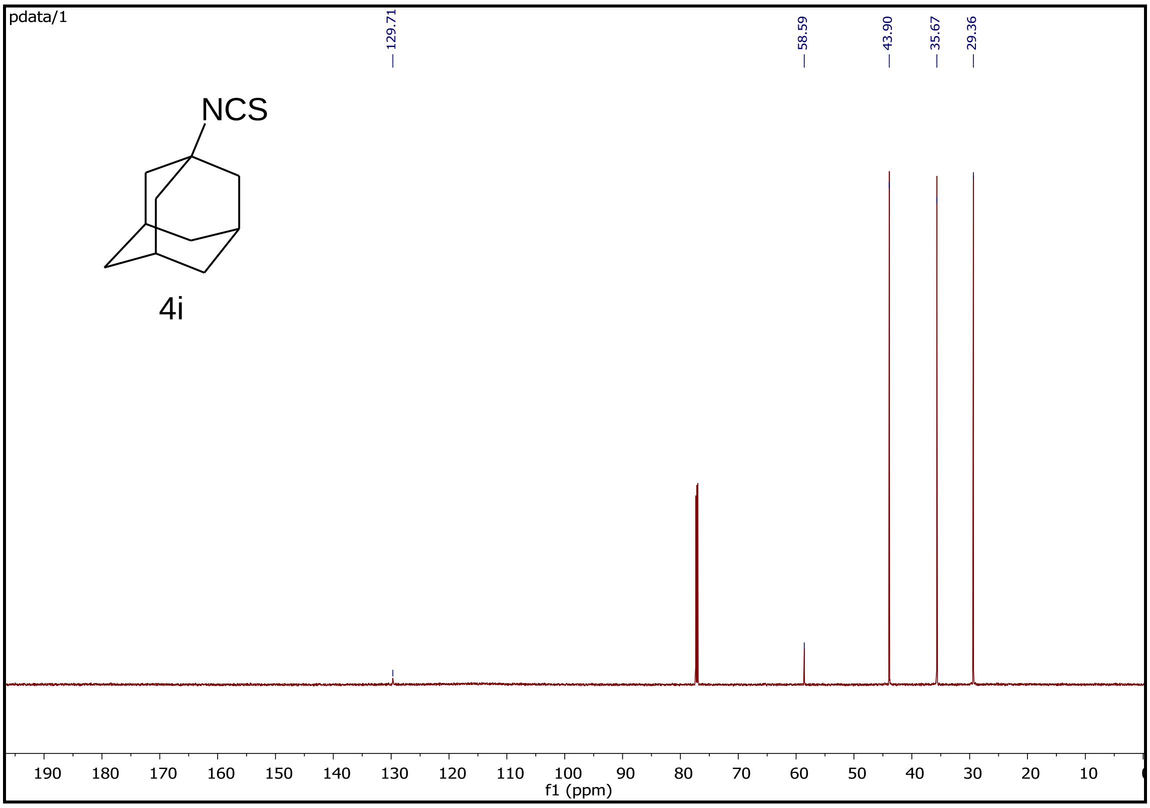

Fig S18. ^{13}C NMR of compound **4i**

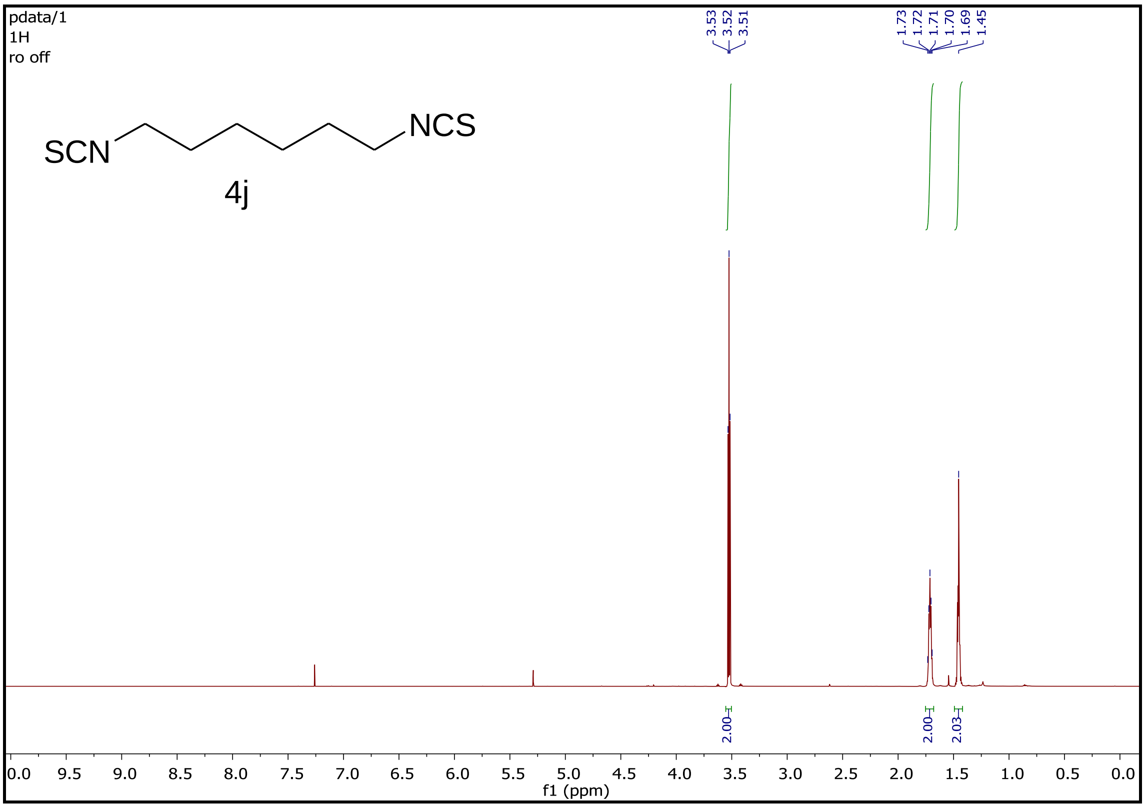

Fig S19. ^{1}H NMR of compound **4j**

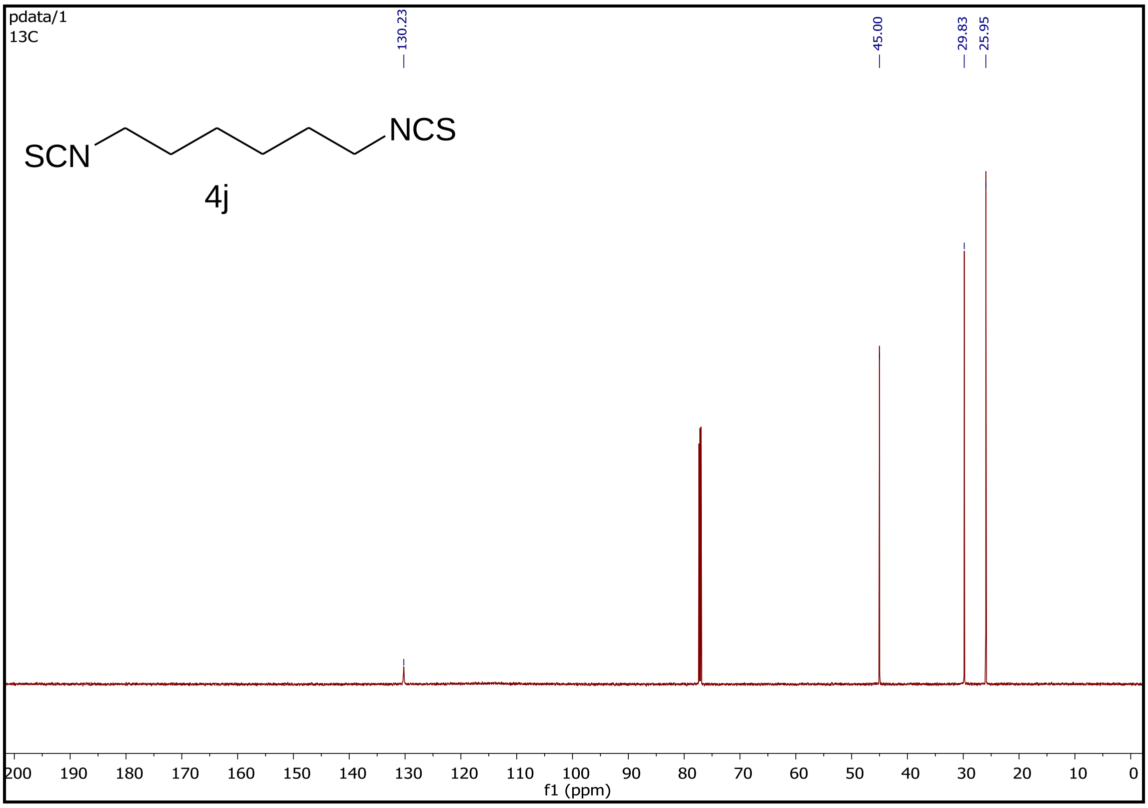

Fig S20. ^{13}C NMR of compound **4j**

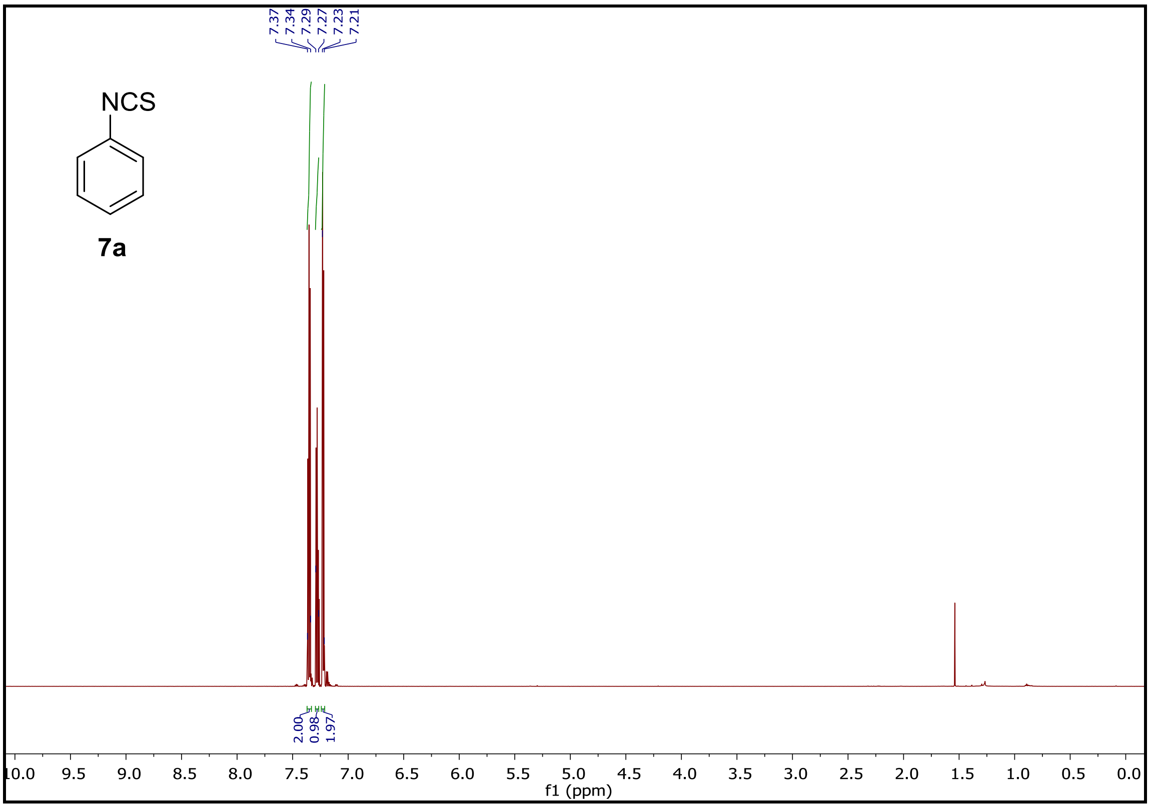

Fig S21. ^{1}H NMR of compound **7a**

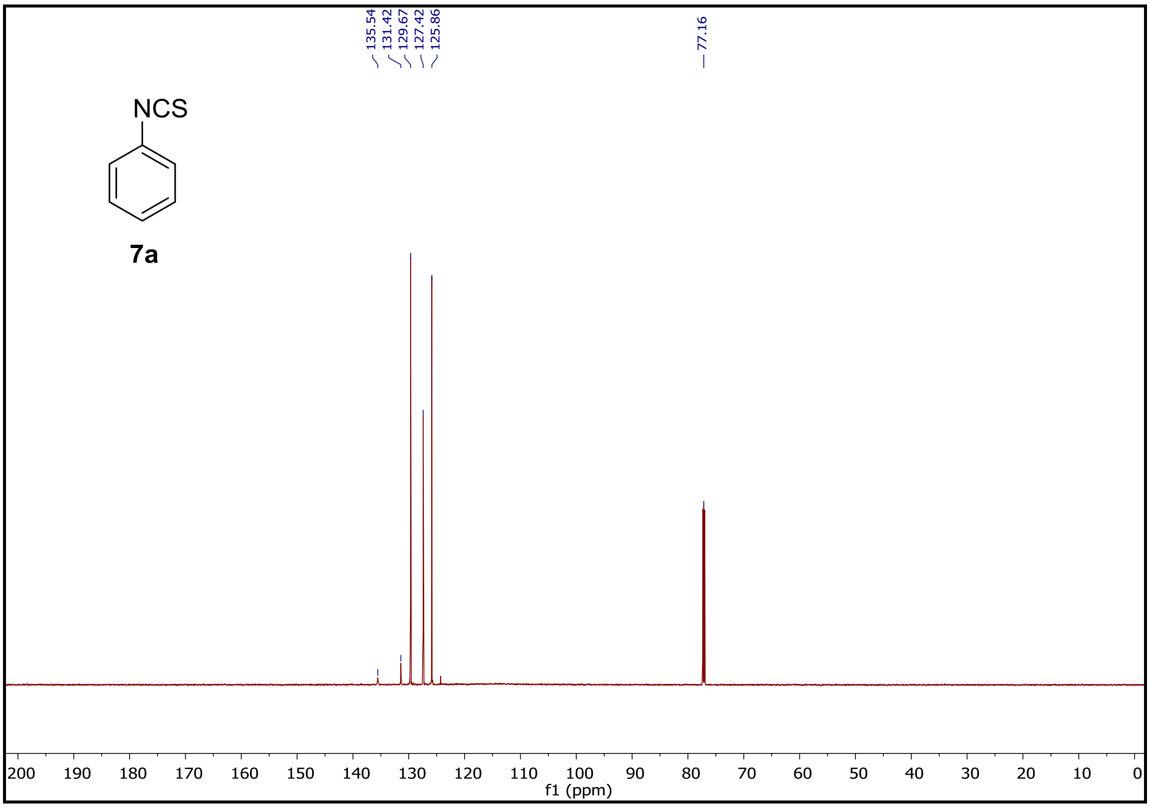

Fig S22. ^{13}C NMR of compound **7a**

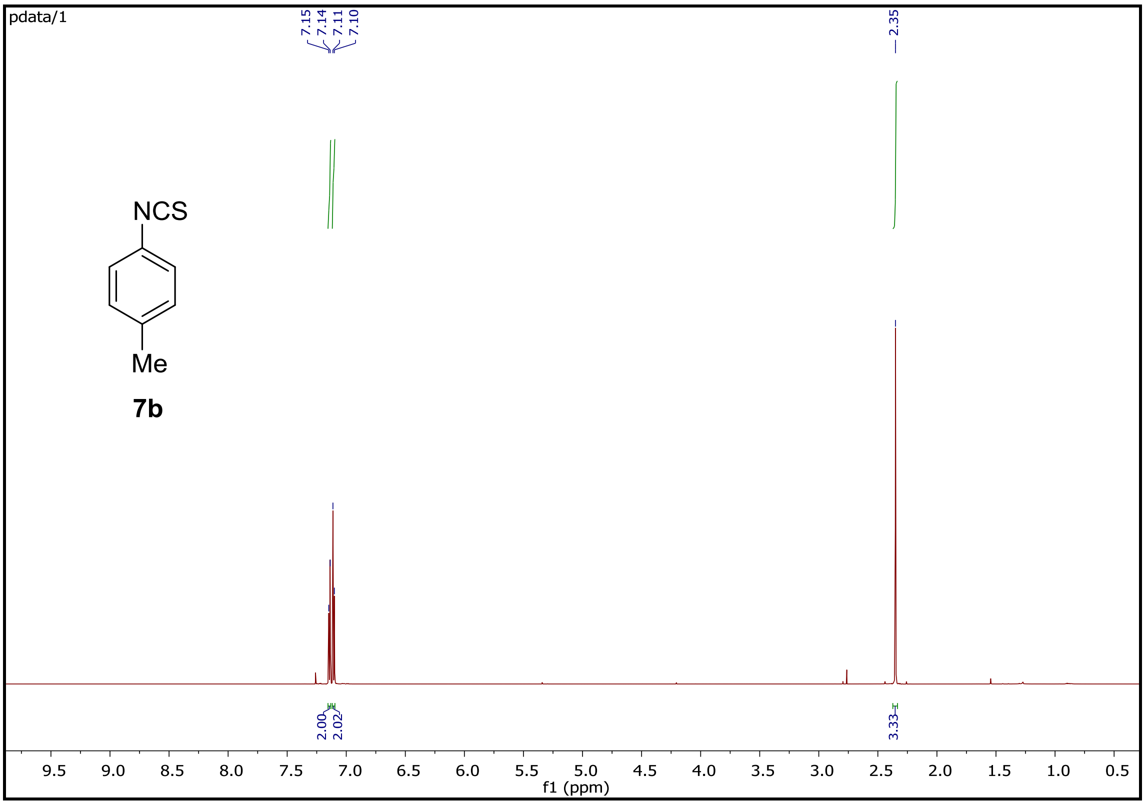

Fig S23. ^{1}H NMR of compound **7b**

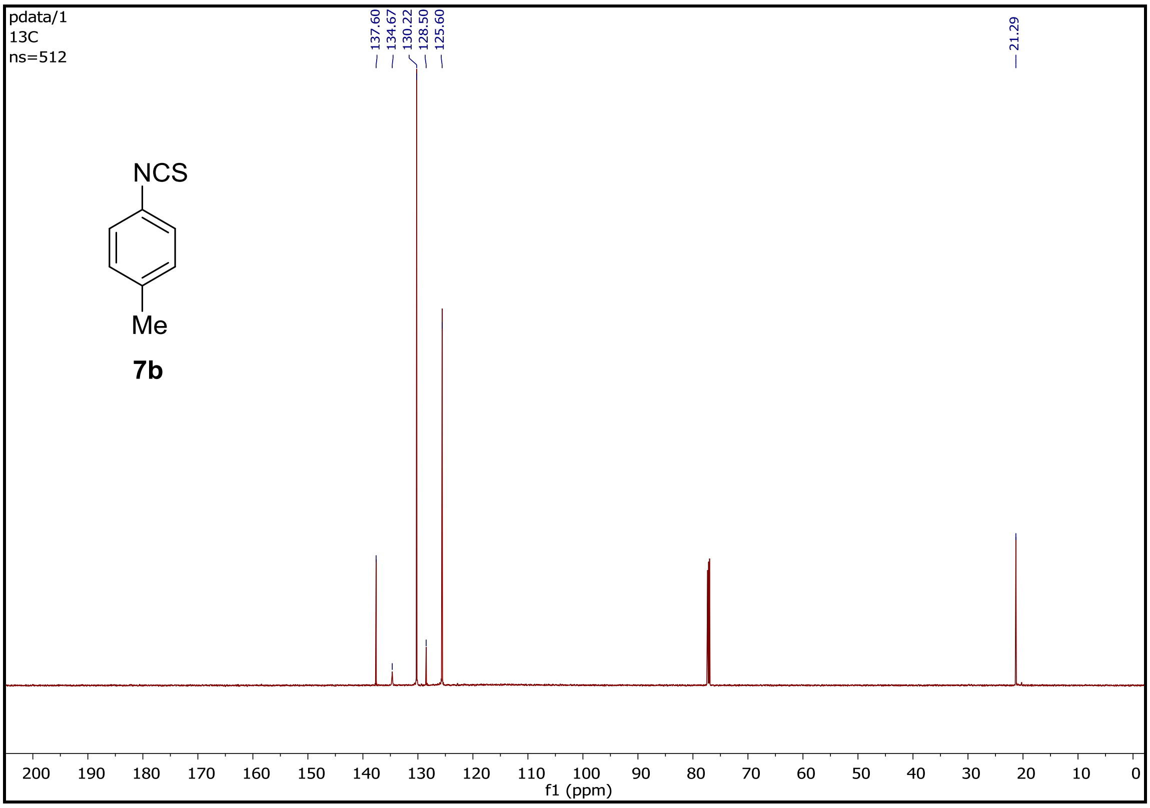

Fig S24. ^{13}C NMR of compound **7b**

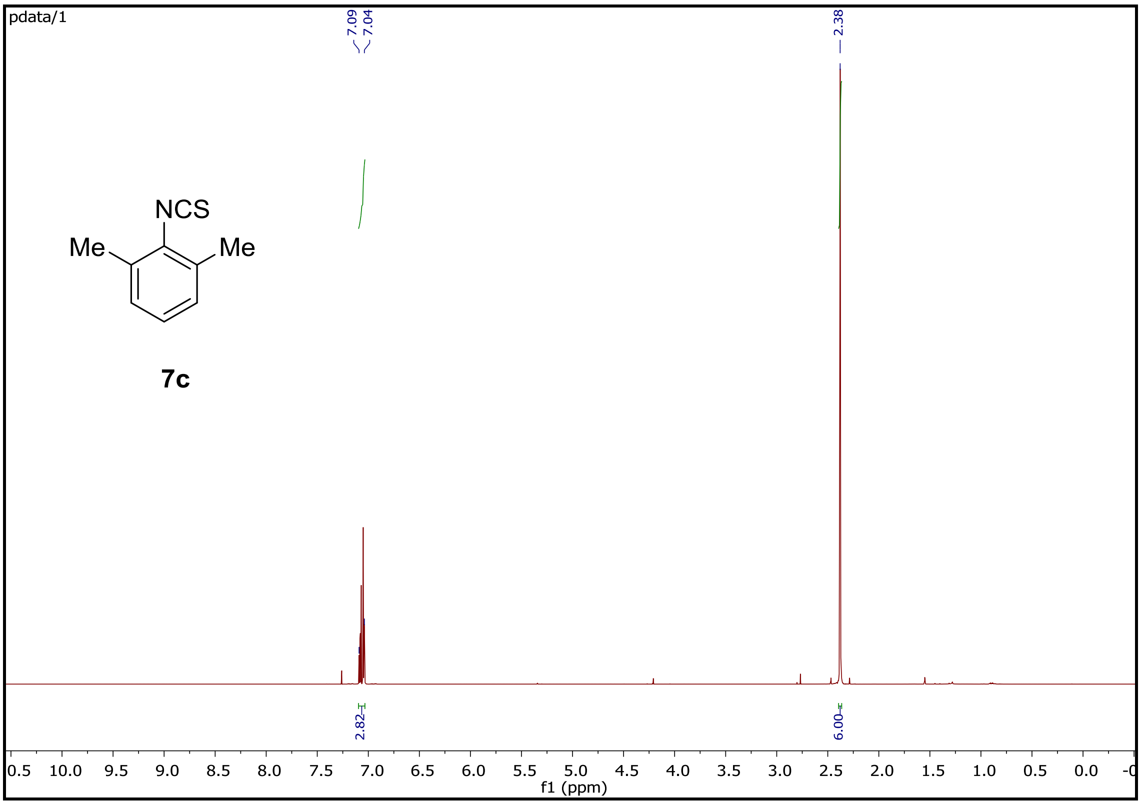

Fig S25. ^{1}H NMR of compound **7c**

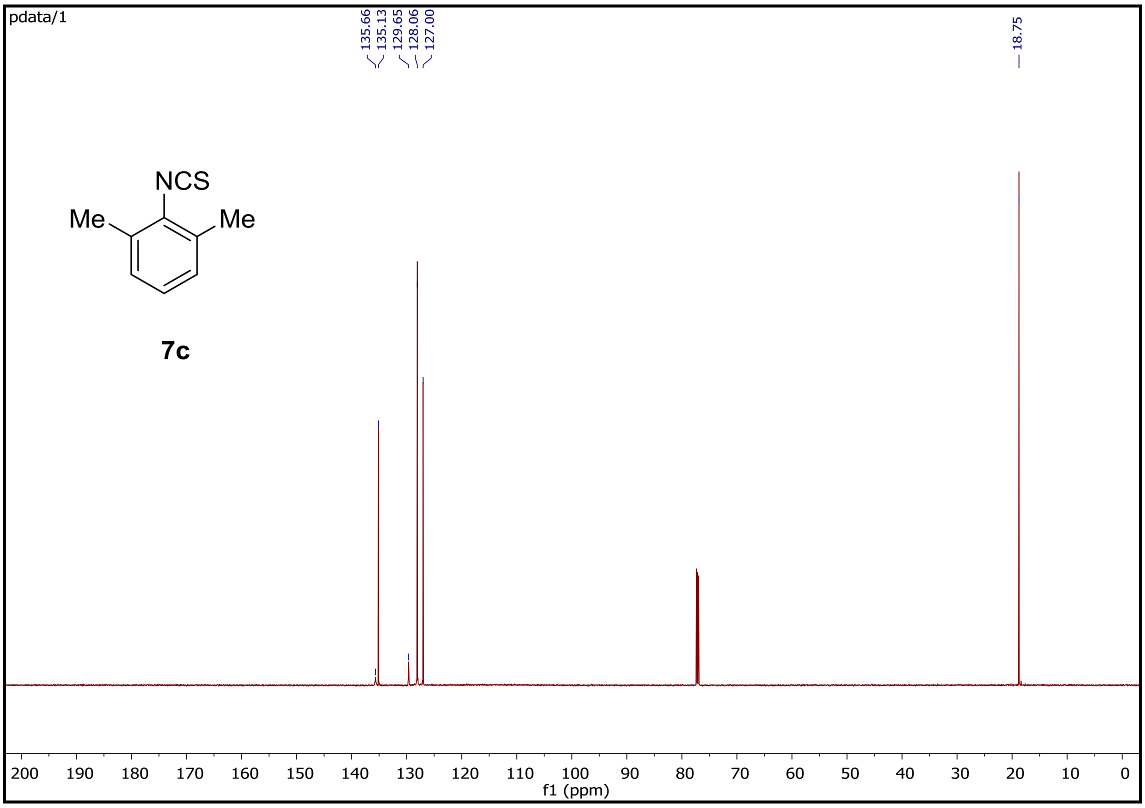

Fig S26. ^{13}C NMR of compound **7c**

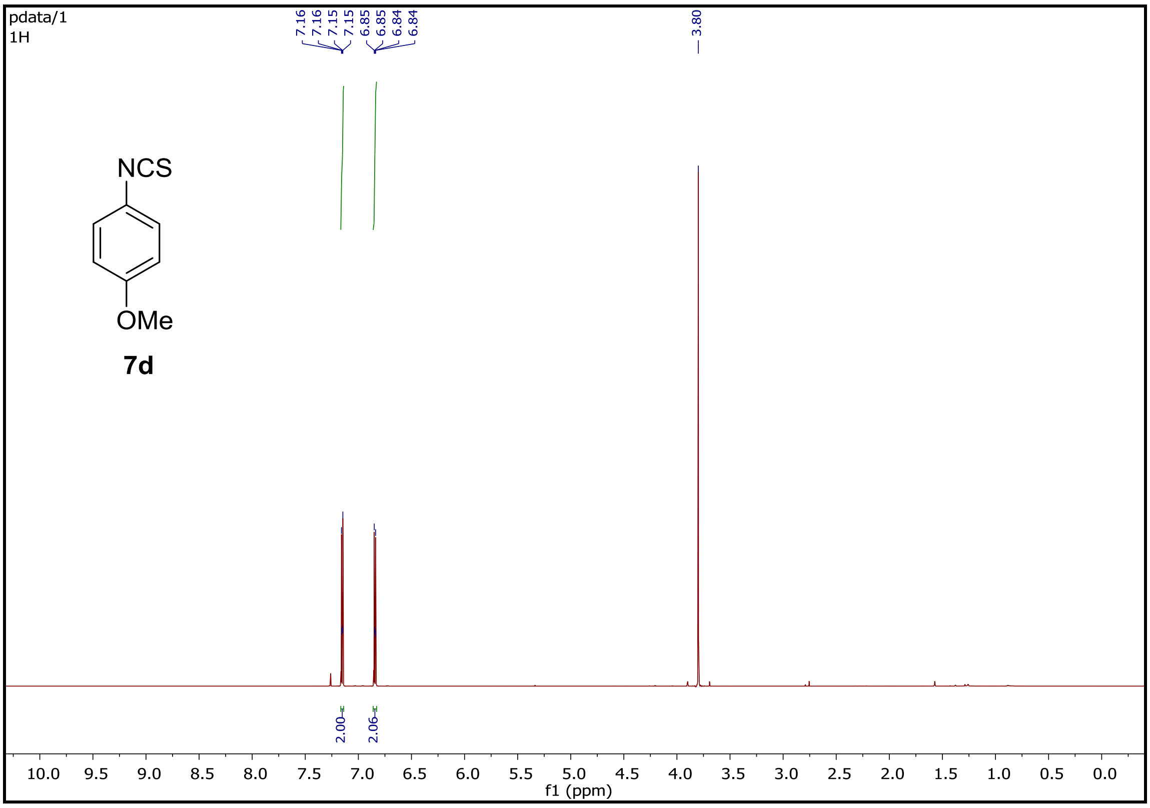

Fig S27. ^{1}H NMR of compound **7d**

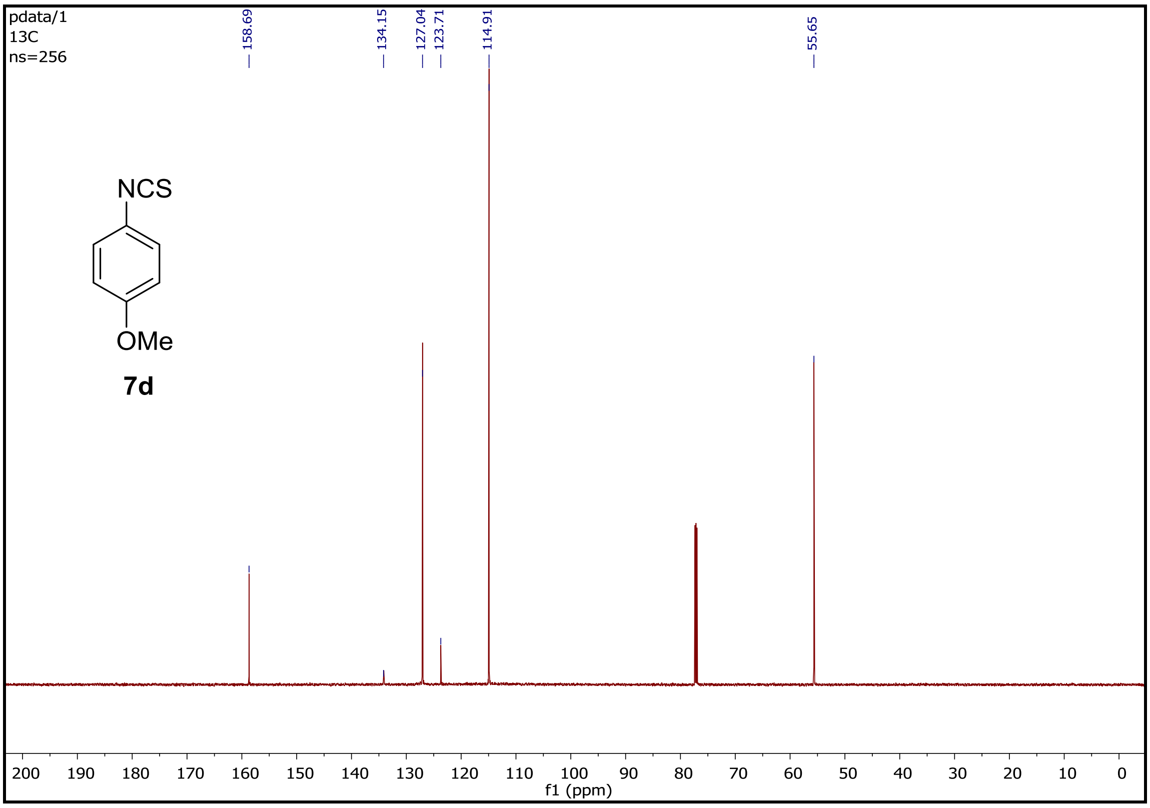

Fig S28. ^{13}C NMR of compound **7d**

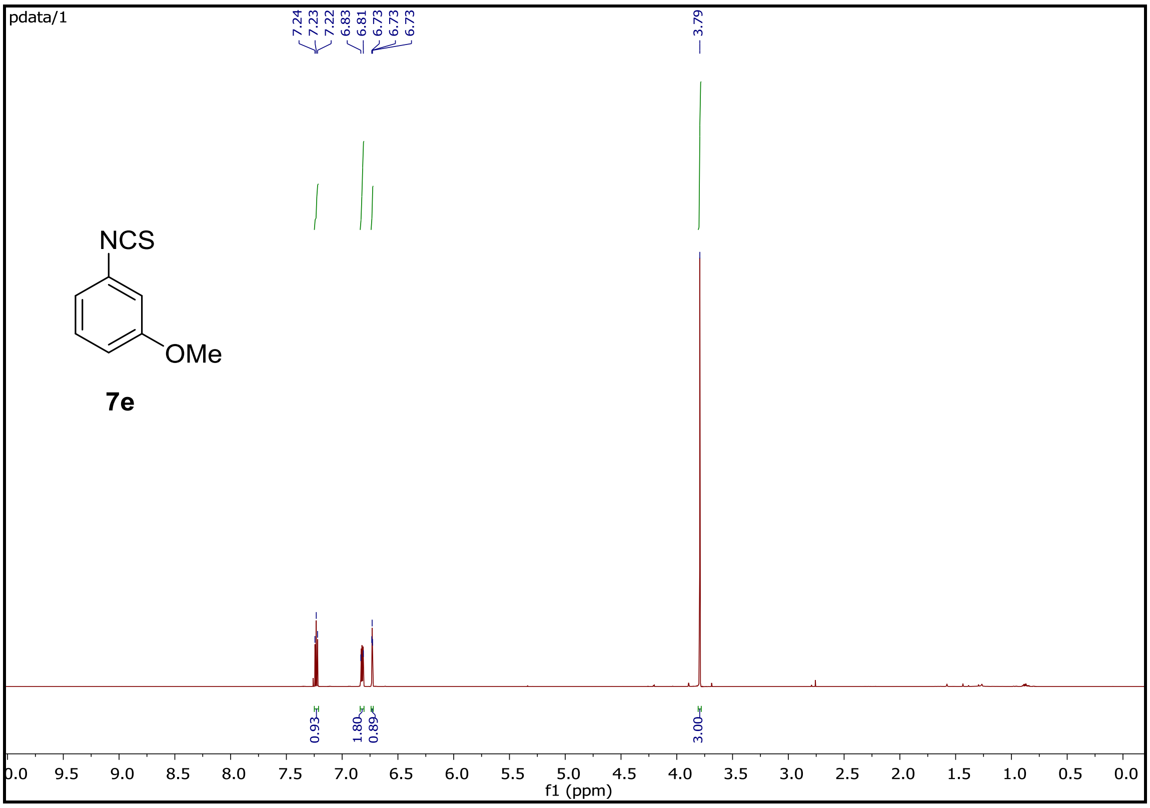

Fig S29. ^{1}H NMR of compound **7e**

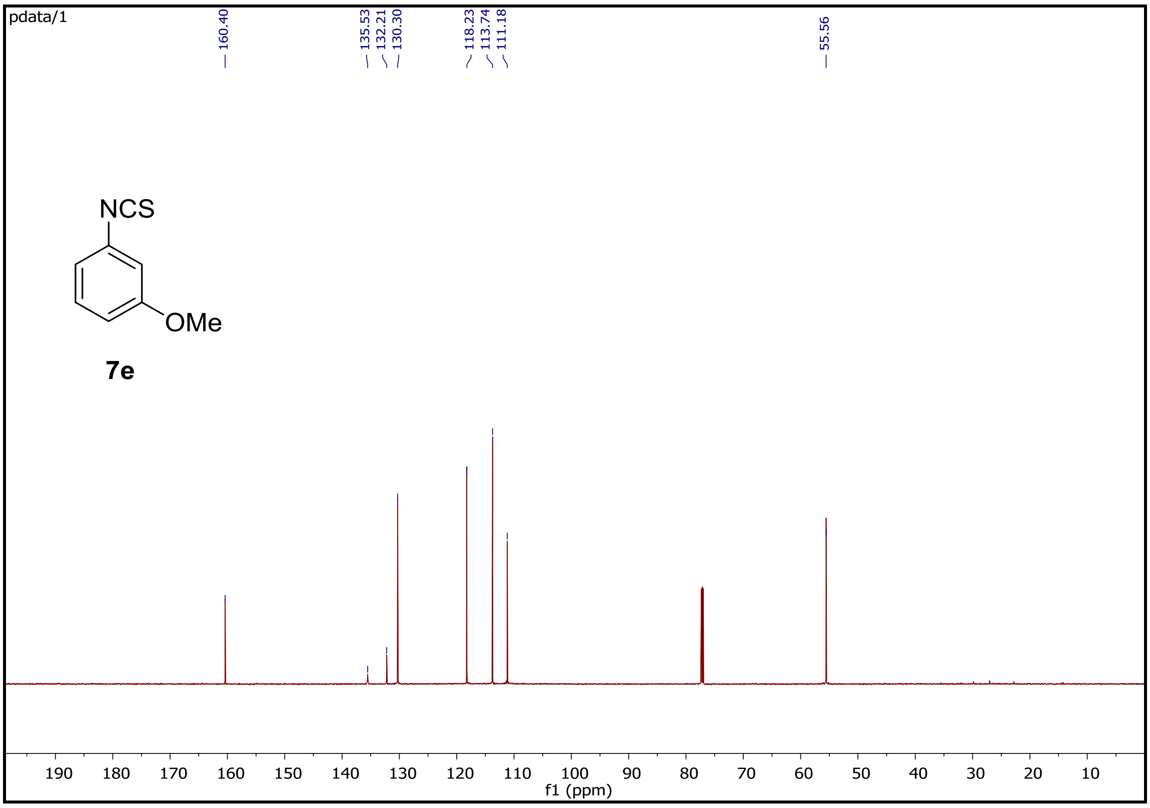

Fig S30. ^{13}C NMR of compound **7e**

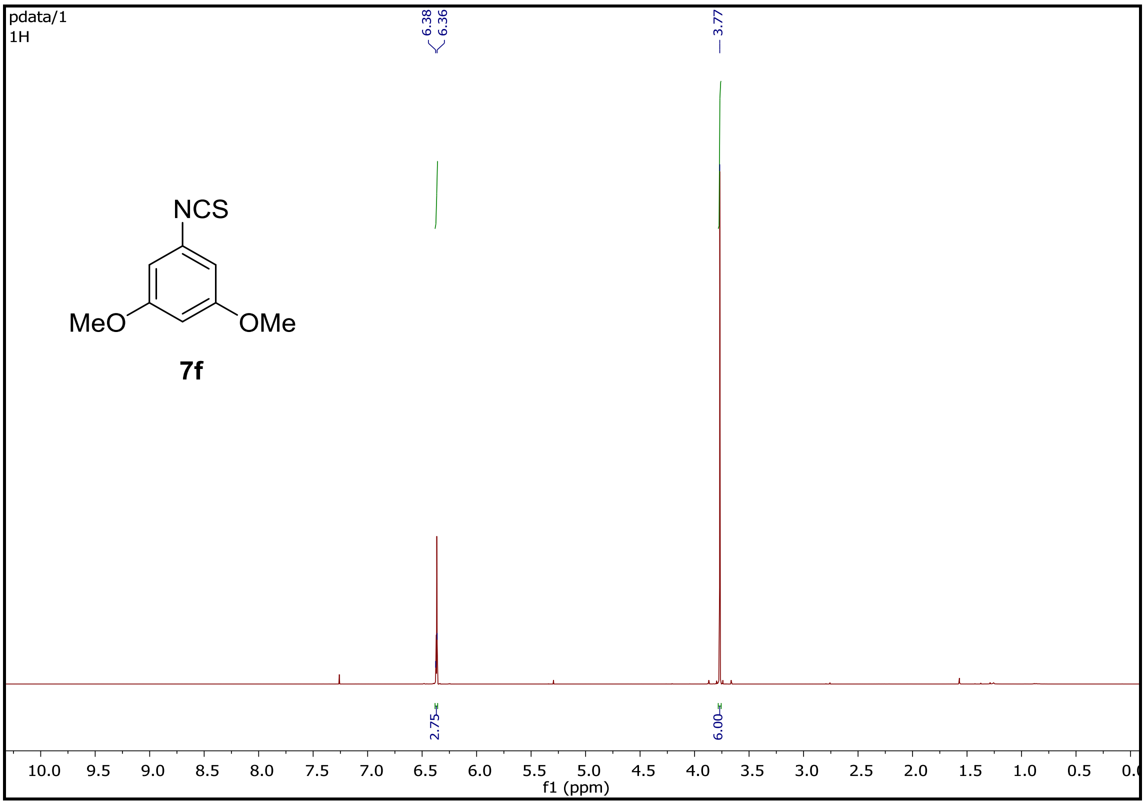

Fig S31. ^{1}H NMR of compound **7f**

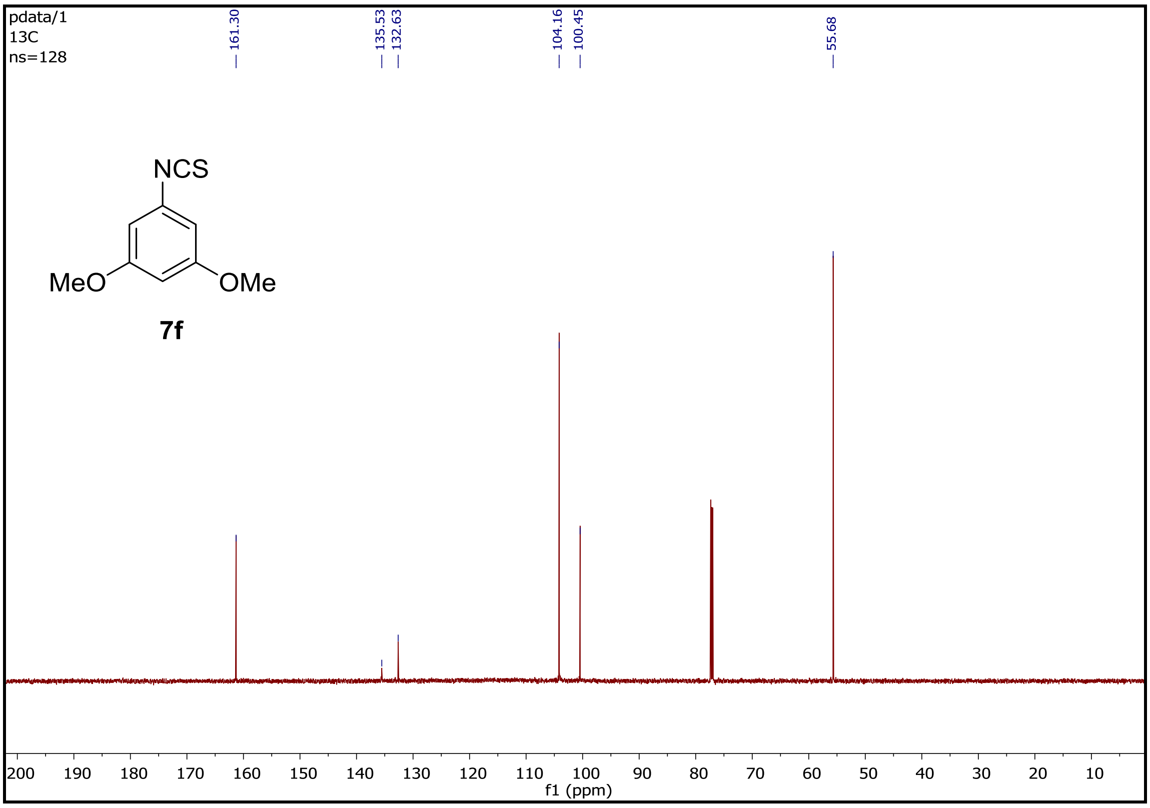

Fig S32. ^{13}C NMR of compound **7f**

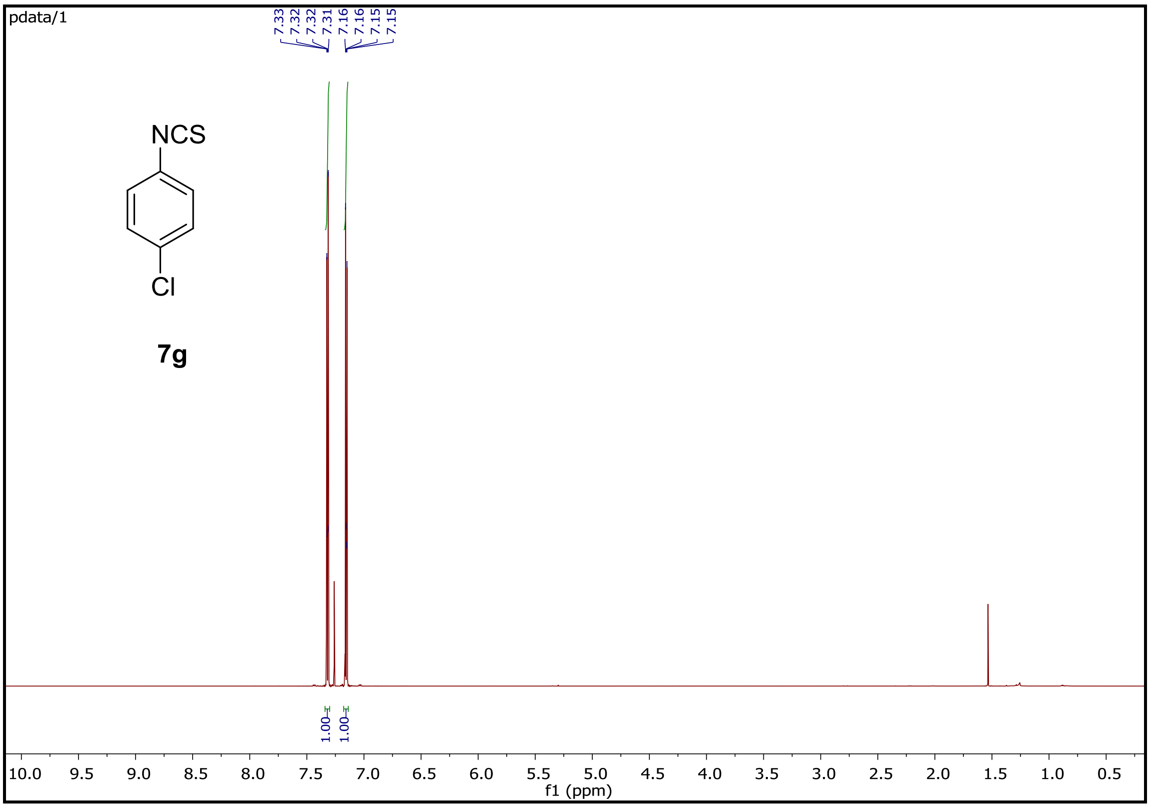

Fig S33. ^{1}H NMR of compound **7g**

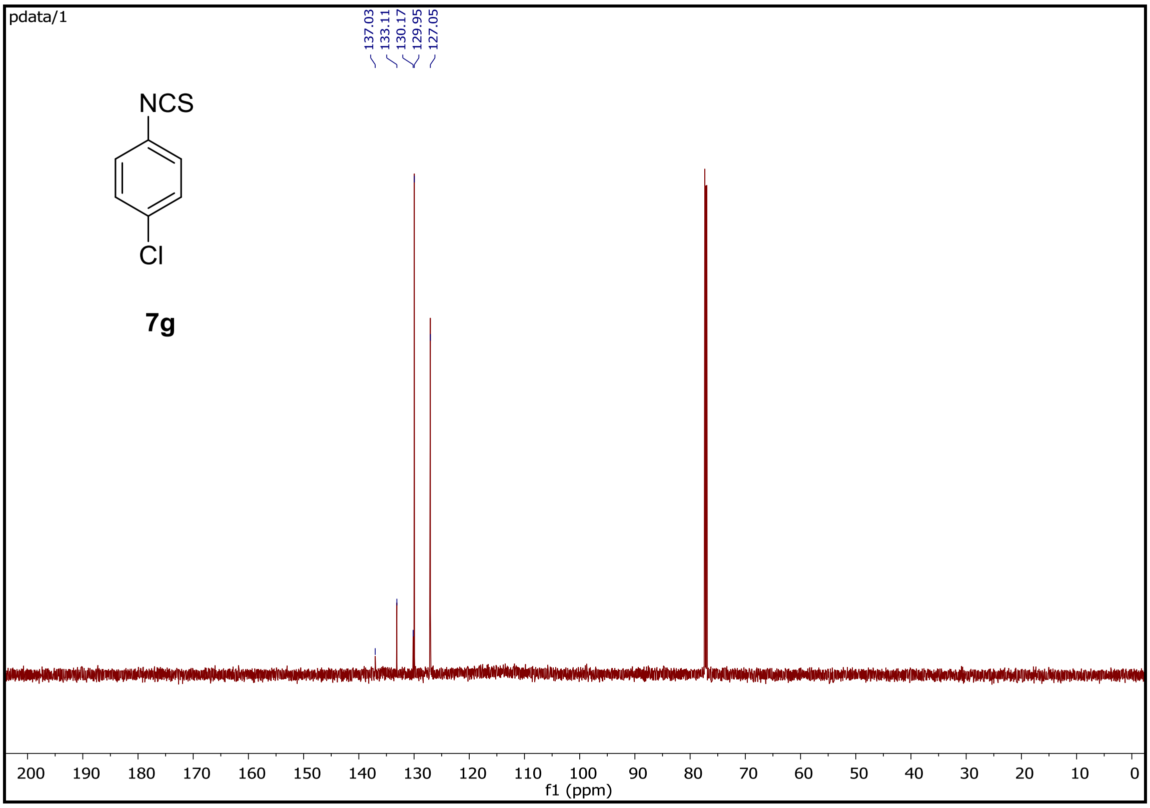

Fig S34. ^{13}C NMR of compound **7g**

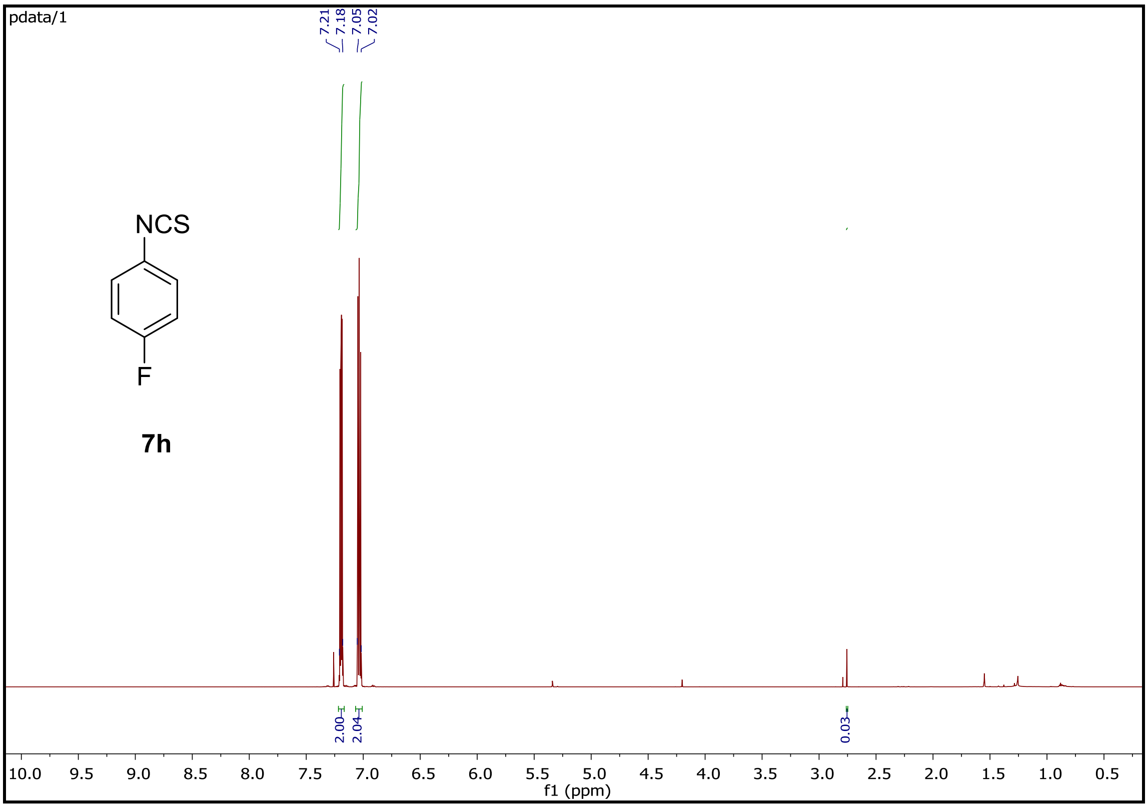

Fig S35. ^{1}H NMR of compound **7h**

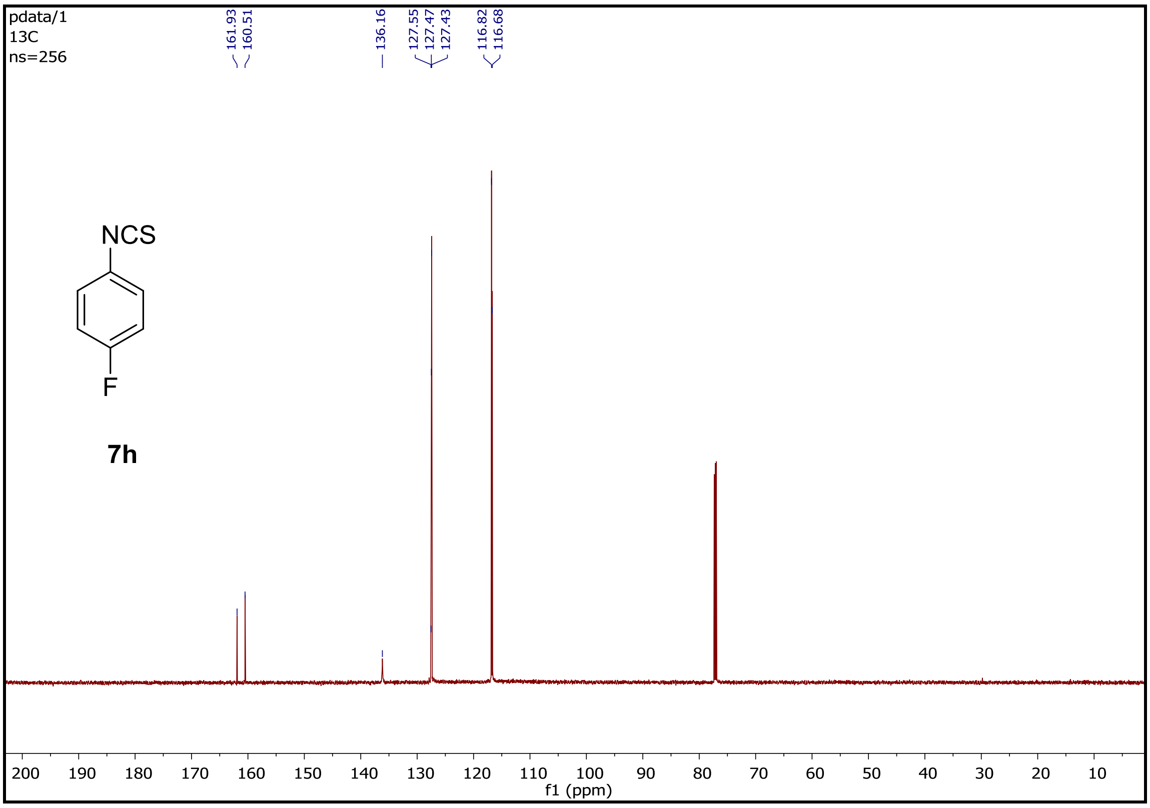

Fig S36. ^{13}C NMR of compound **7h**

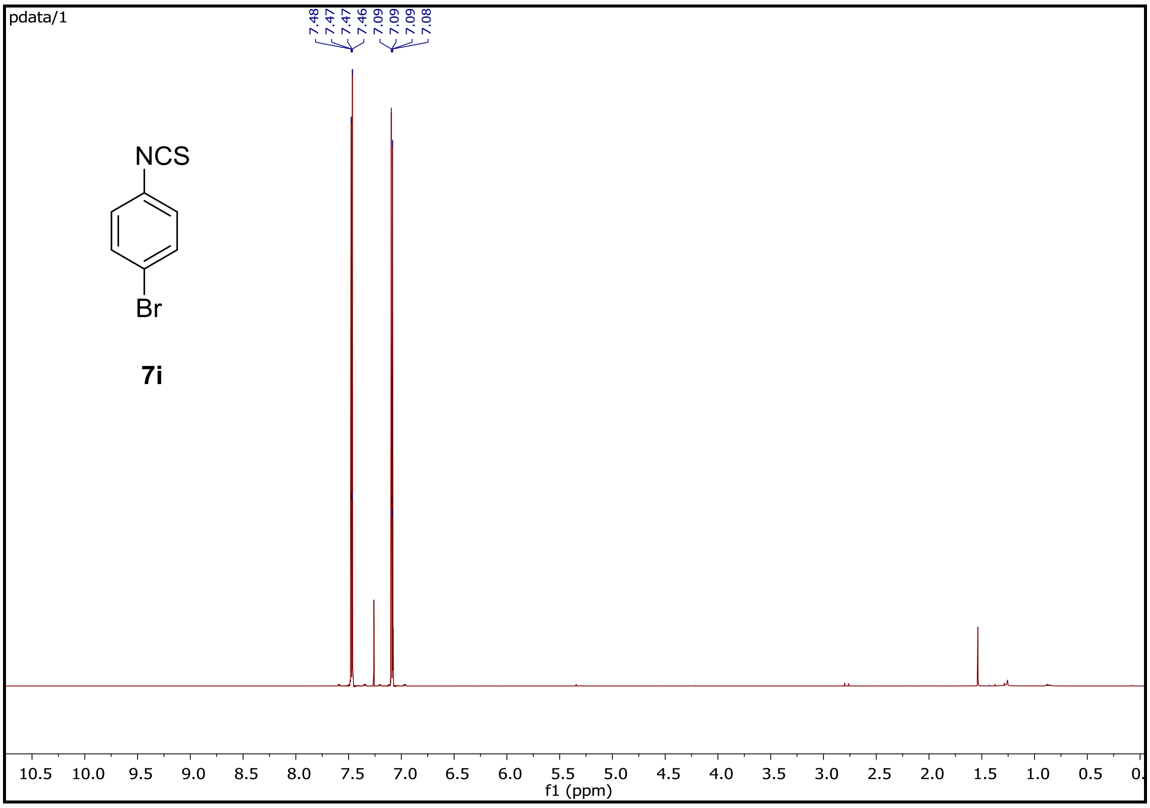

Fig S37. ^{1}H NMR of compound **7i**

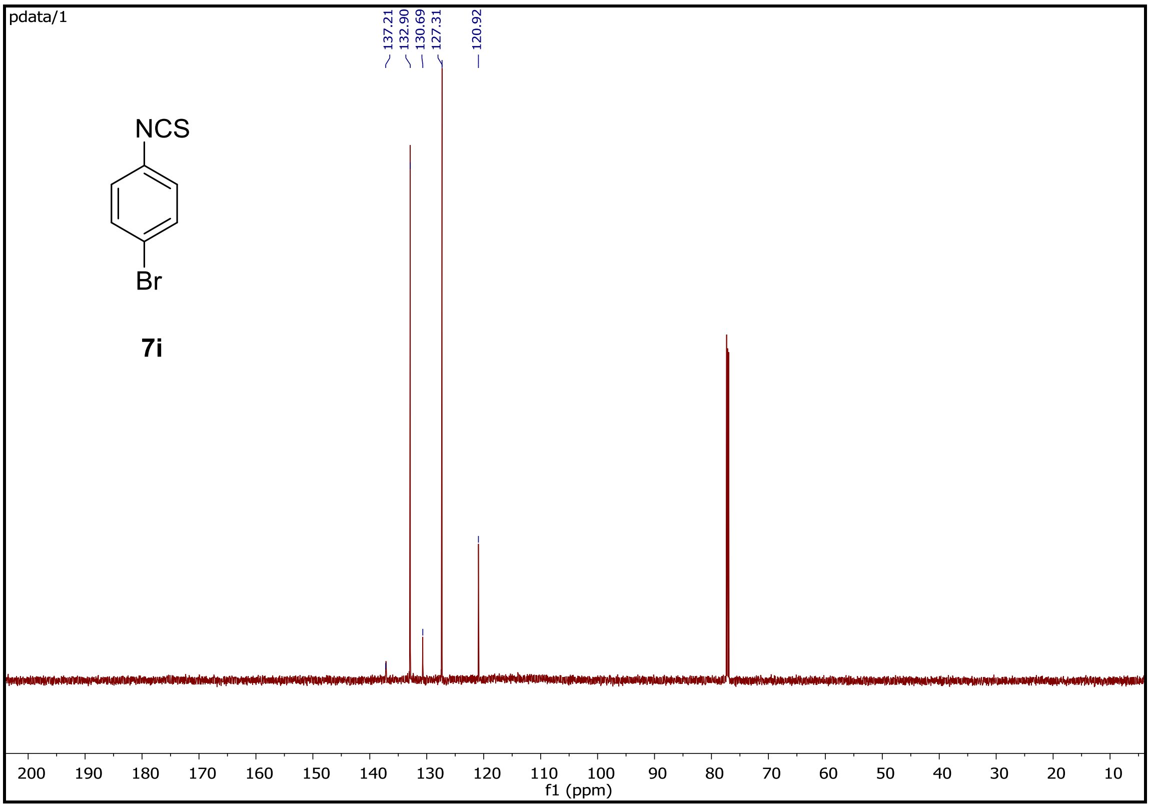

Fig S38. ^{13}C NMR of compound **7i**

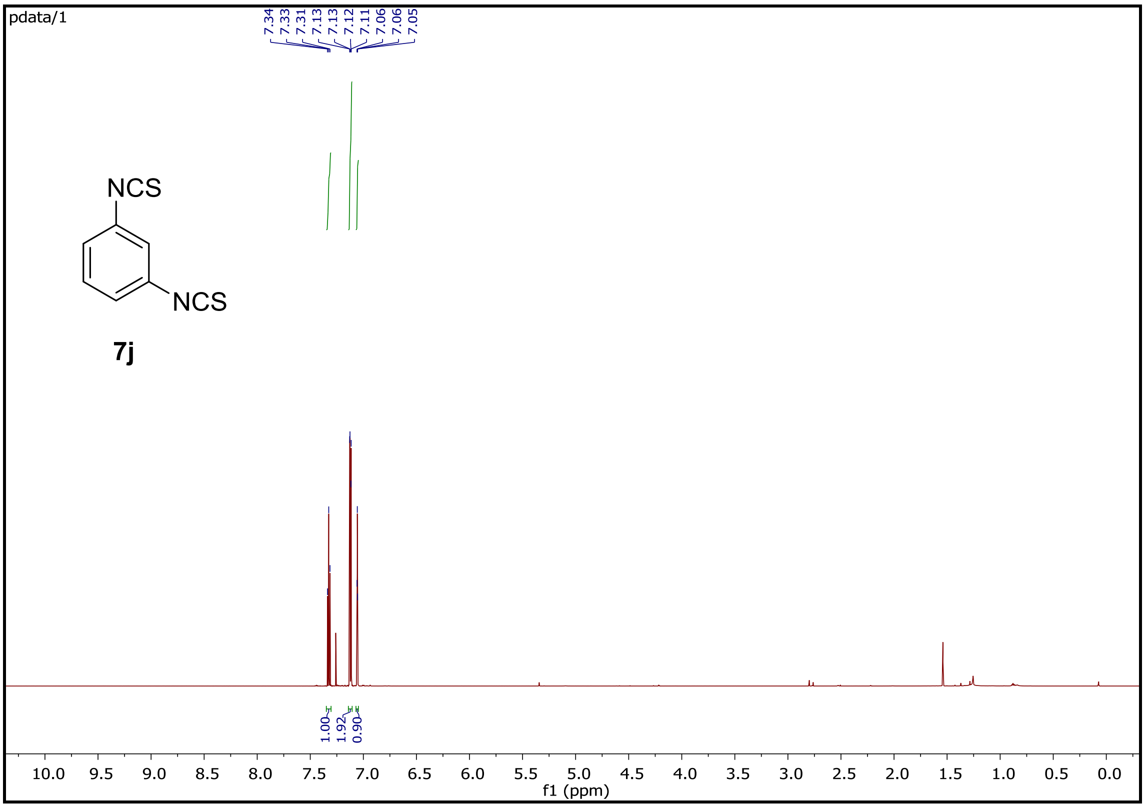

Fig S39. ^{1}H NMR of compound **7j**

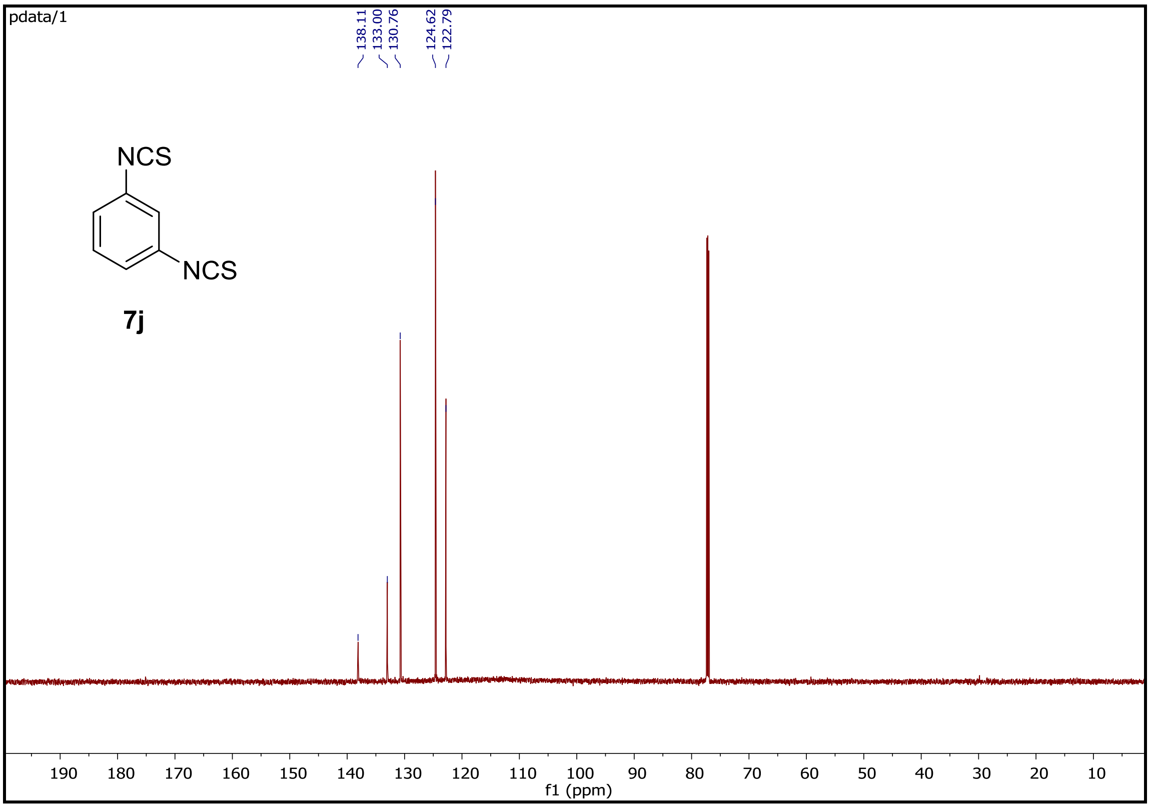

Fig S40. ^{13}C NMR of compound **7j**

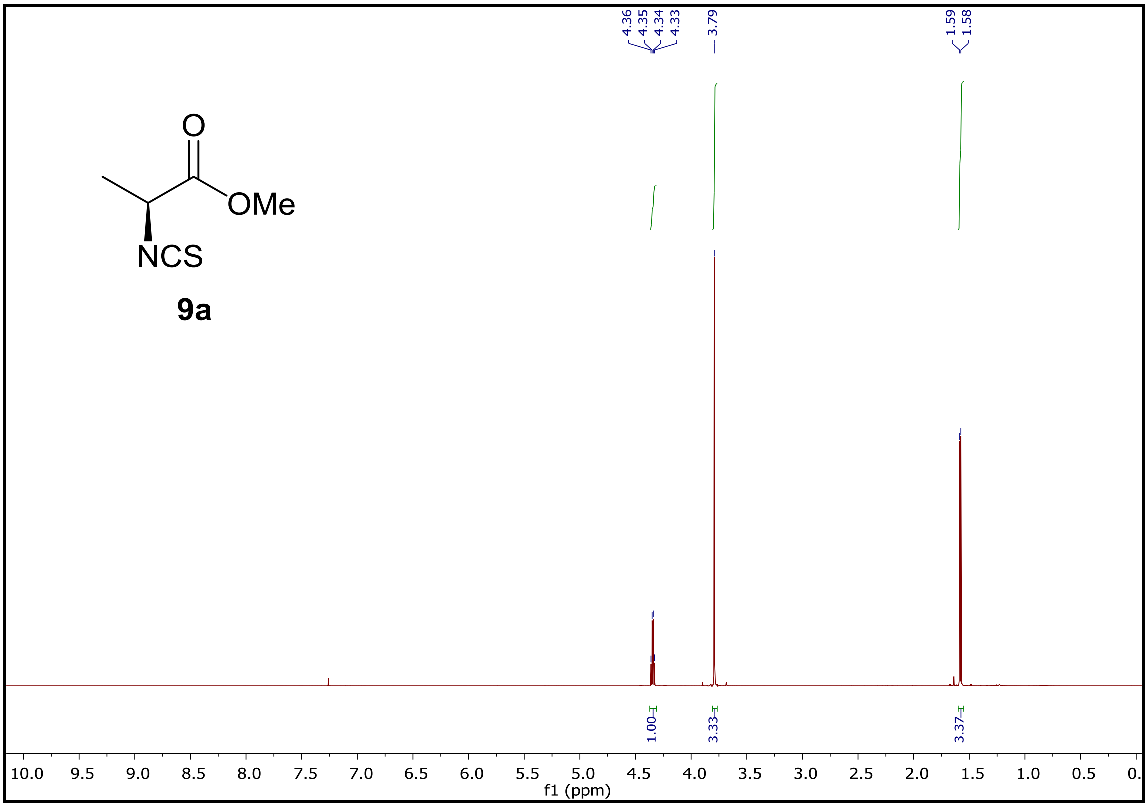

Fig S41. ^{1}H NMR of compound **9a**

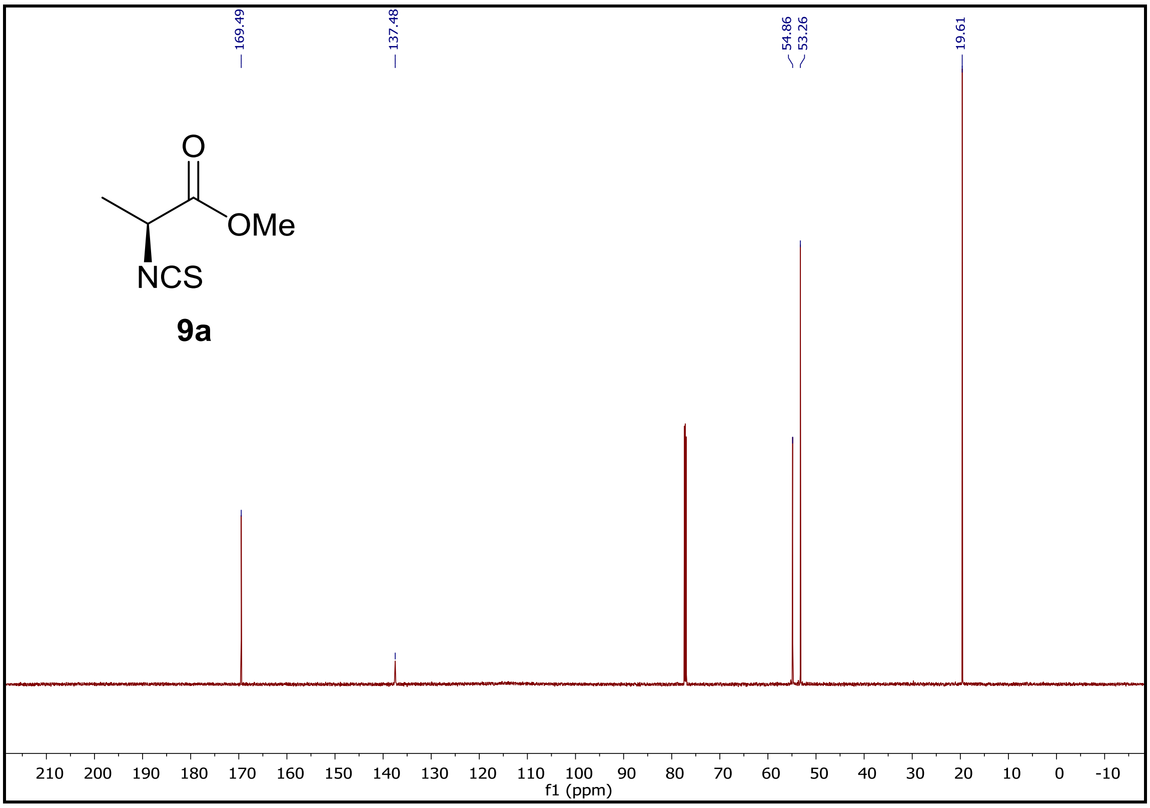

Fig S42. ^{13}C NMR of compound **9a**

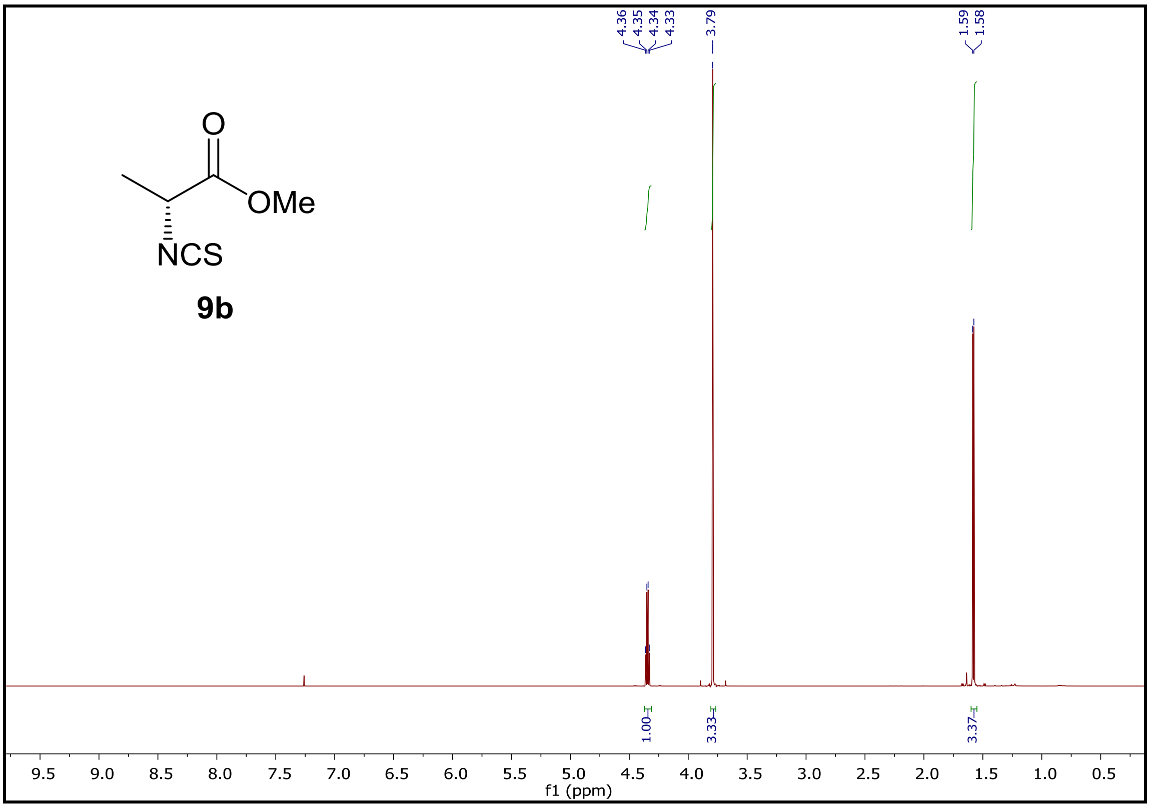

Fig S43. ^{1}H NMR of compound **9b**

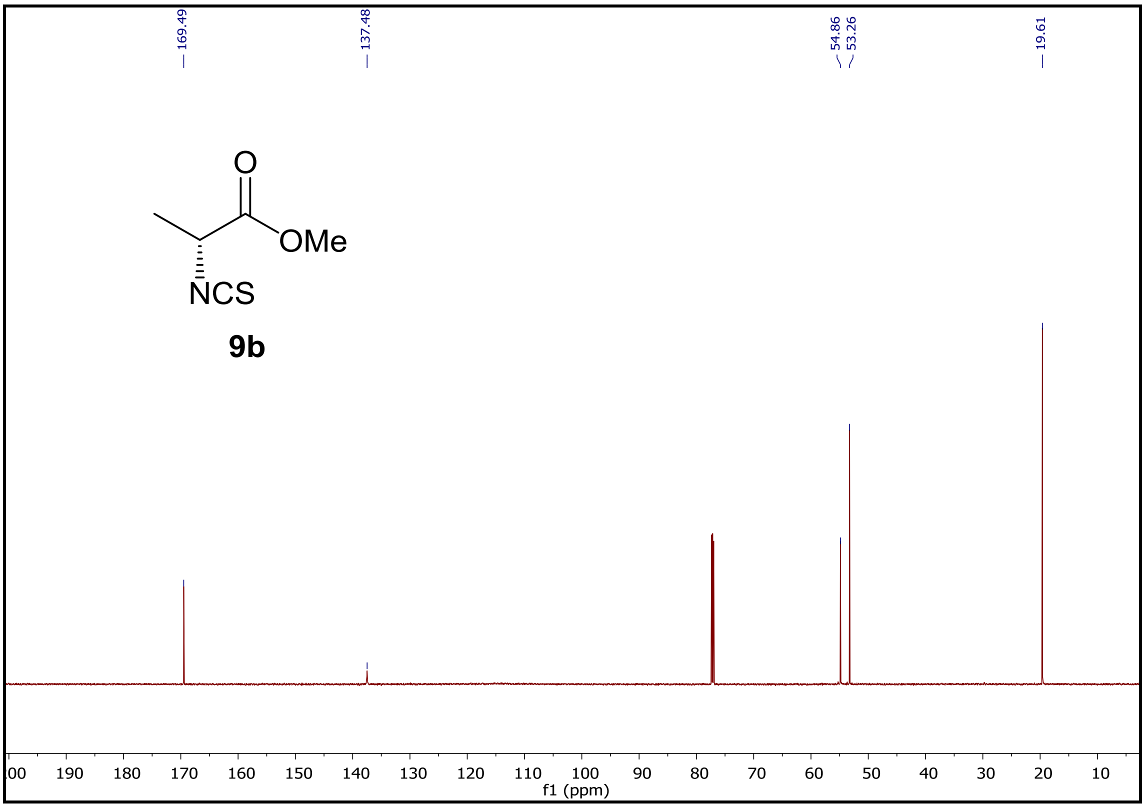

Fig S44. ^{13}C NMR of compound **9b**

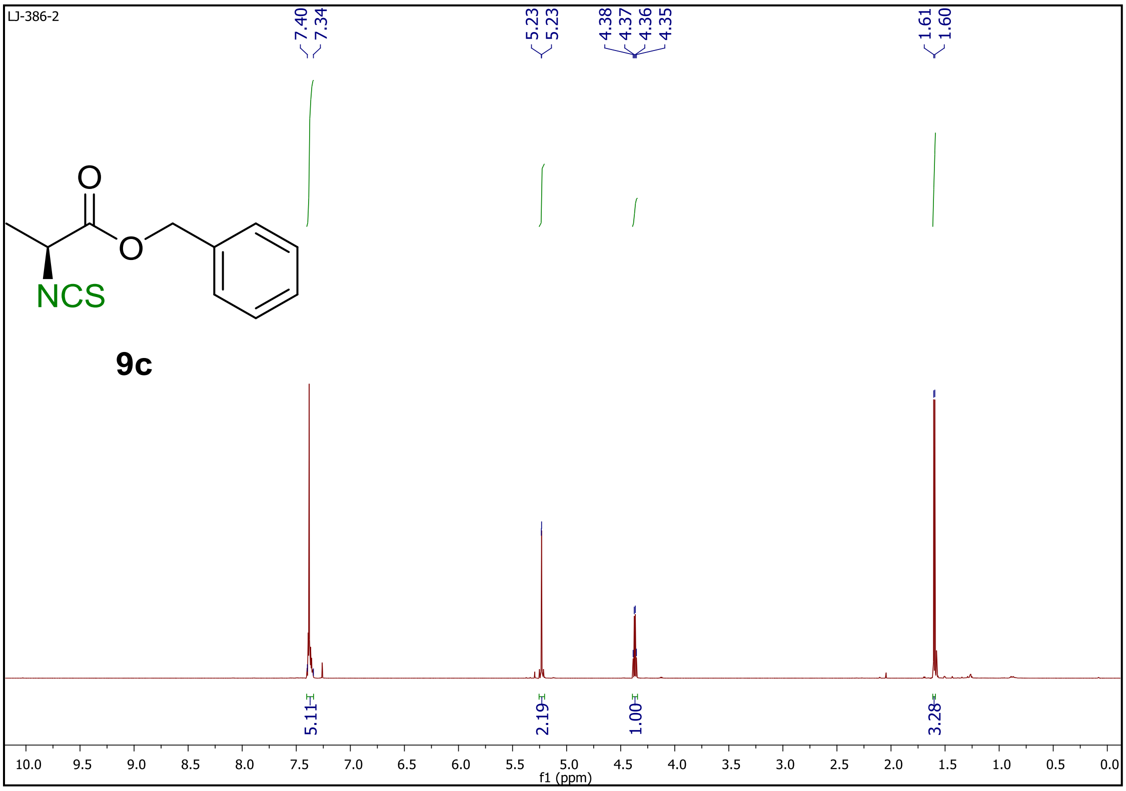

Fig S45. ^{1}H NMR of compound **9c**

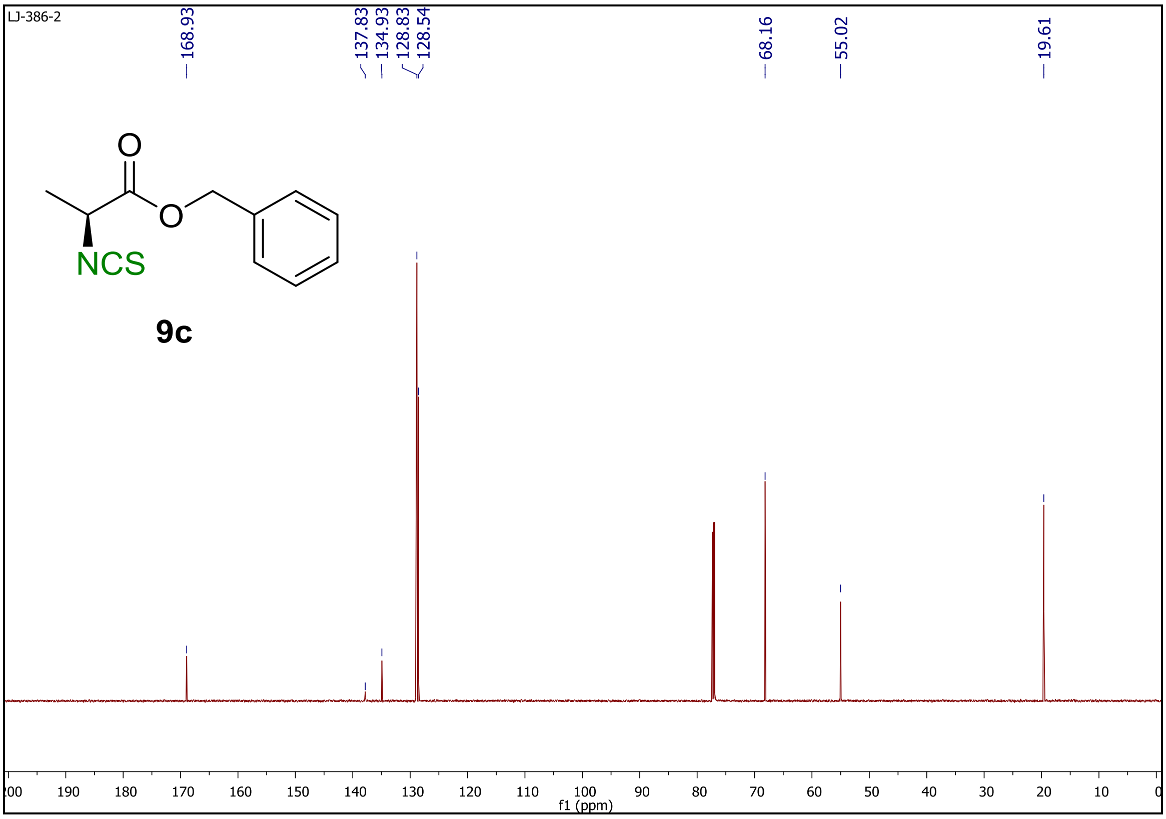

Fig S46. ^{13}C NMR of compound **9c**

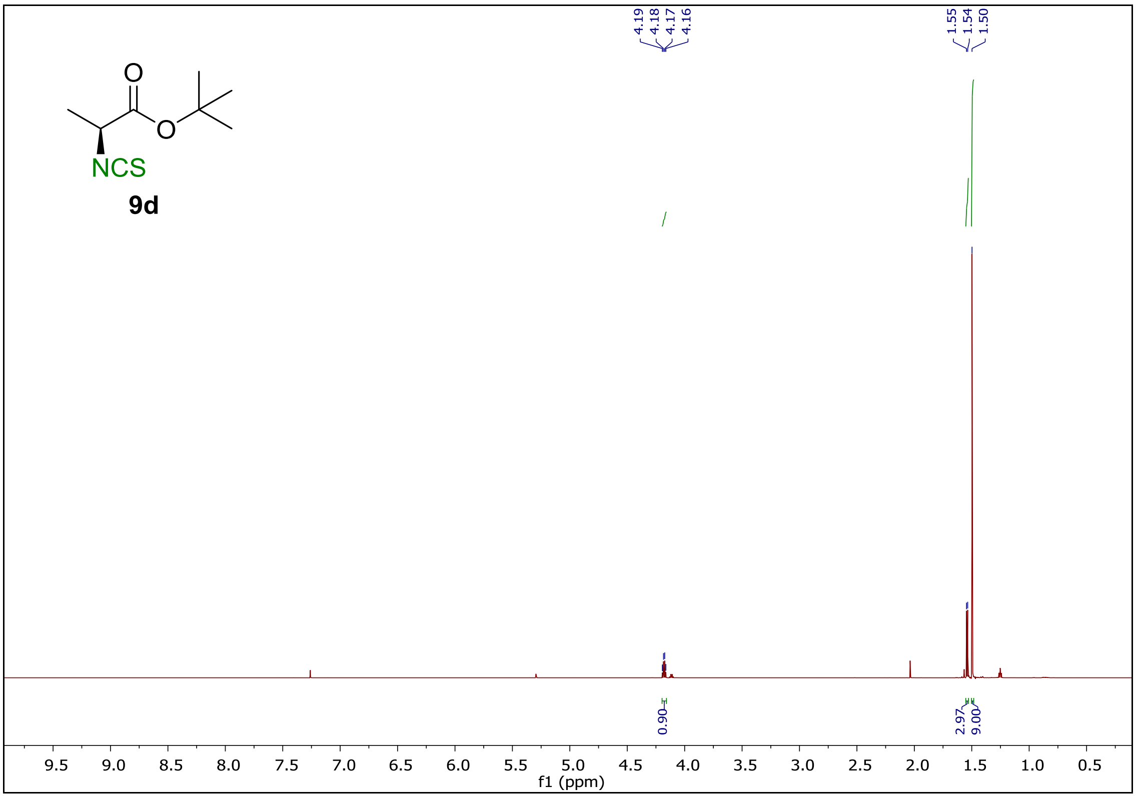

Fig S47. ^{1}H NMR of compound **9d**

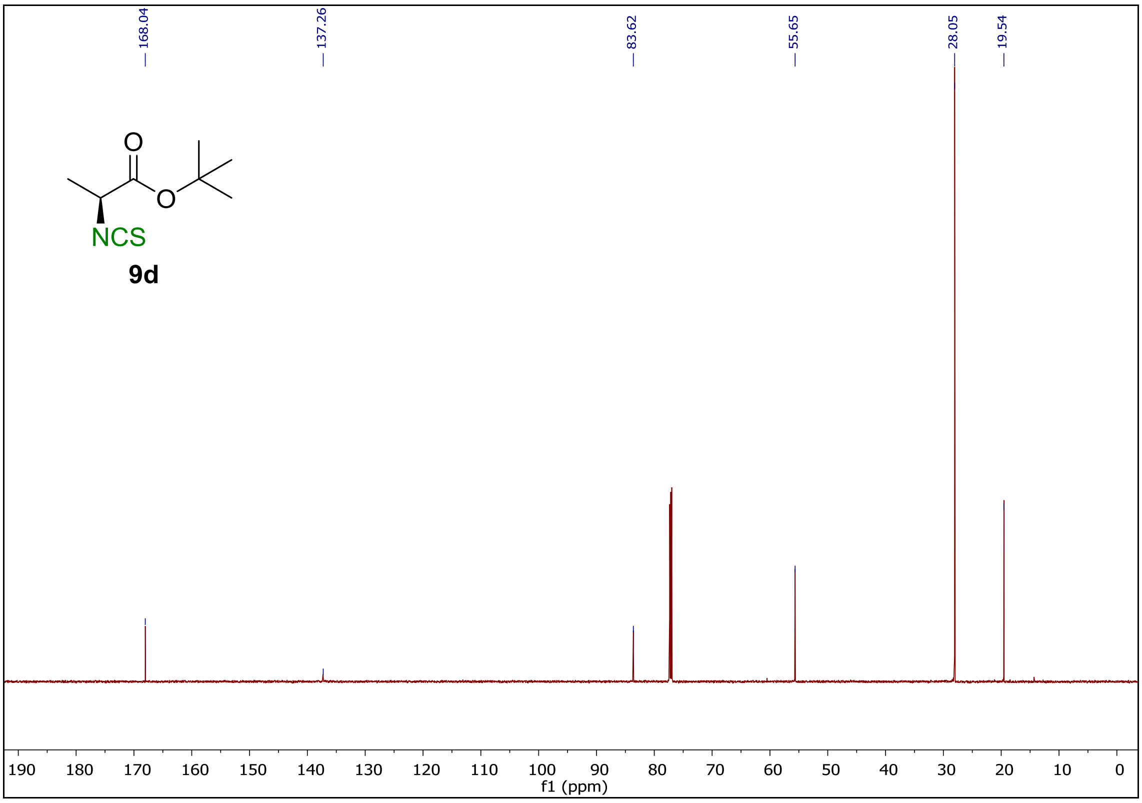

Fig S48. ^{13}C NMR of compound **9d**

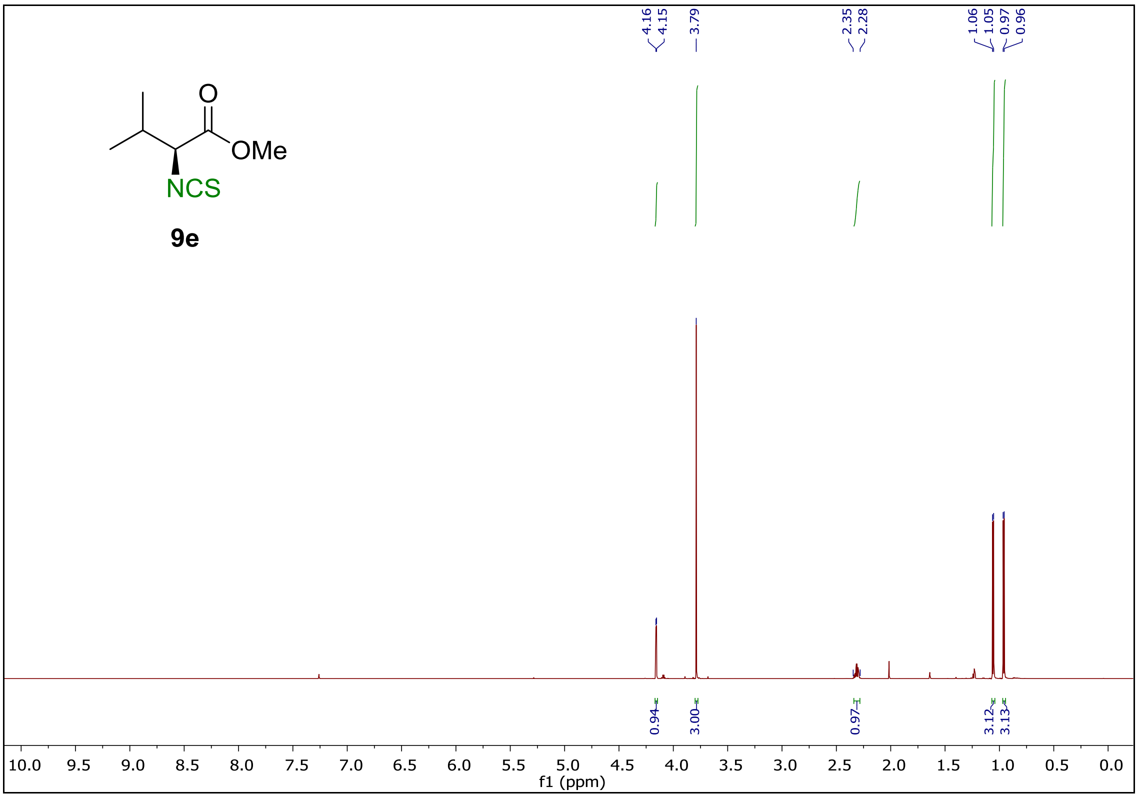

Fig S49. ^{1}H NMR of compound **9e**

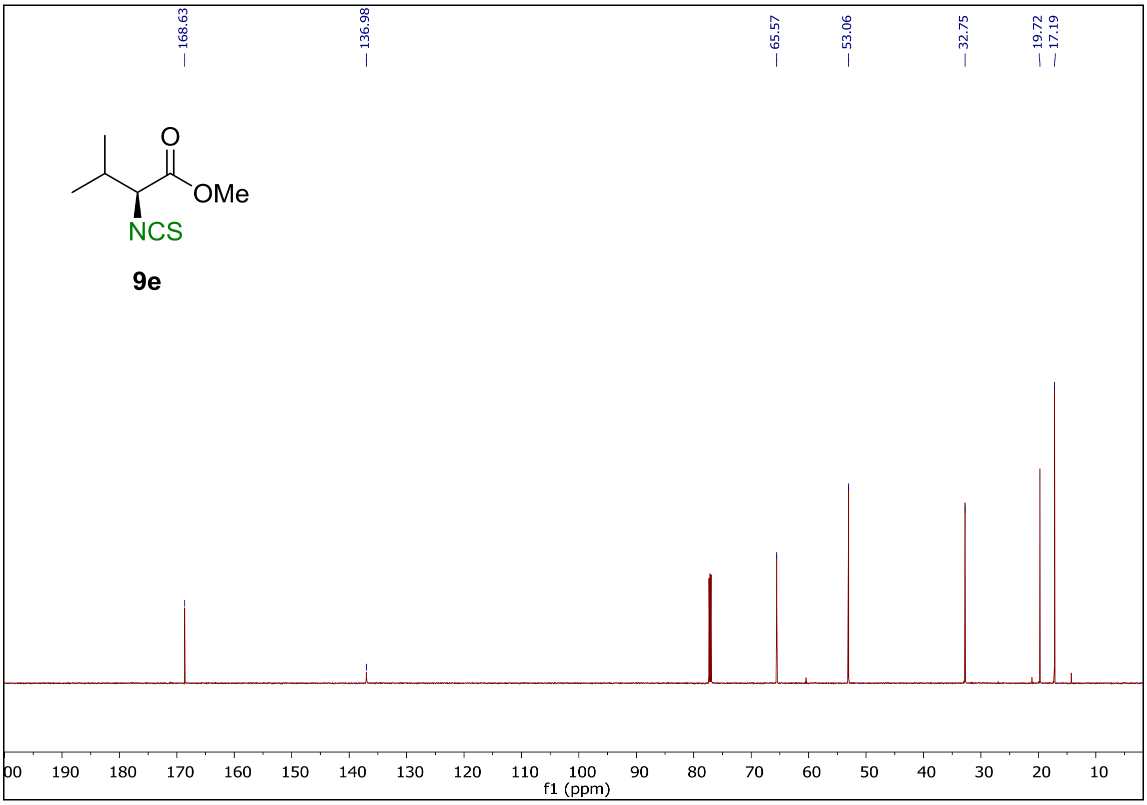

Fig S50. ^{13}C NMR of compound **9e**

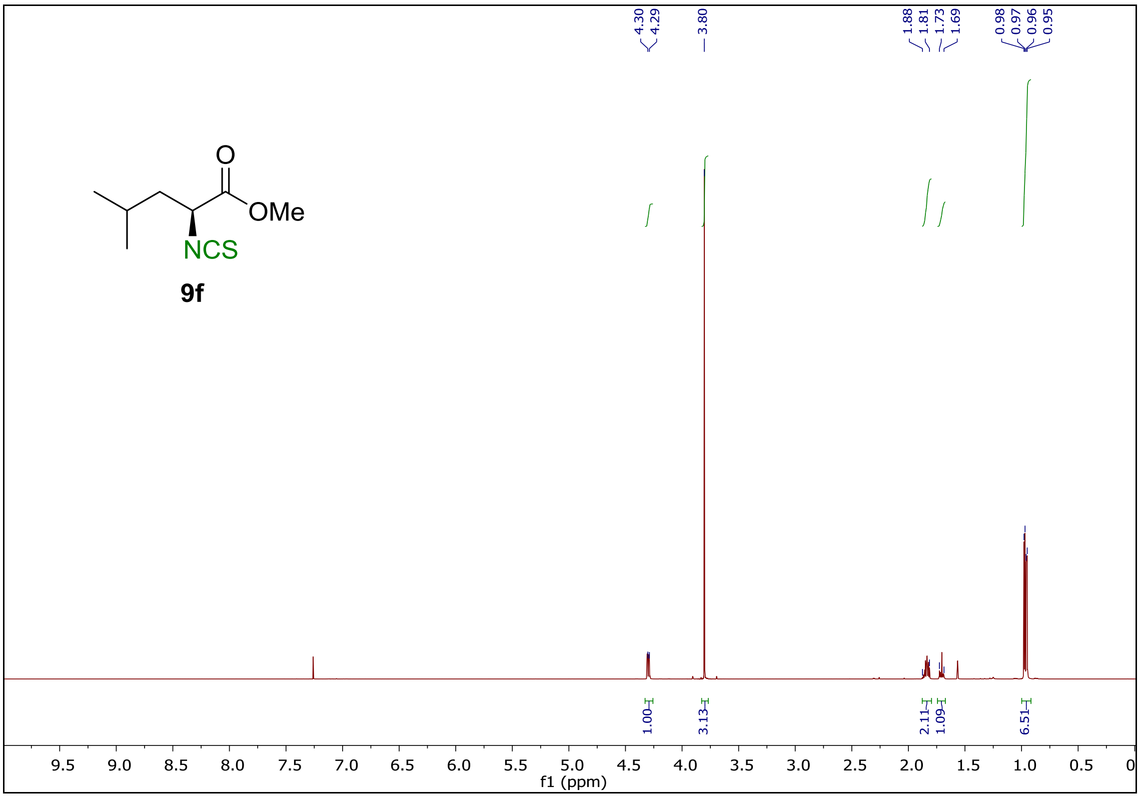

Fig S51. ^{1}H NMR of compound **9f**

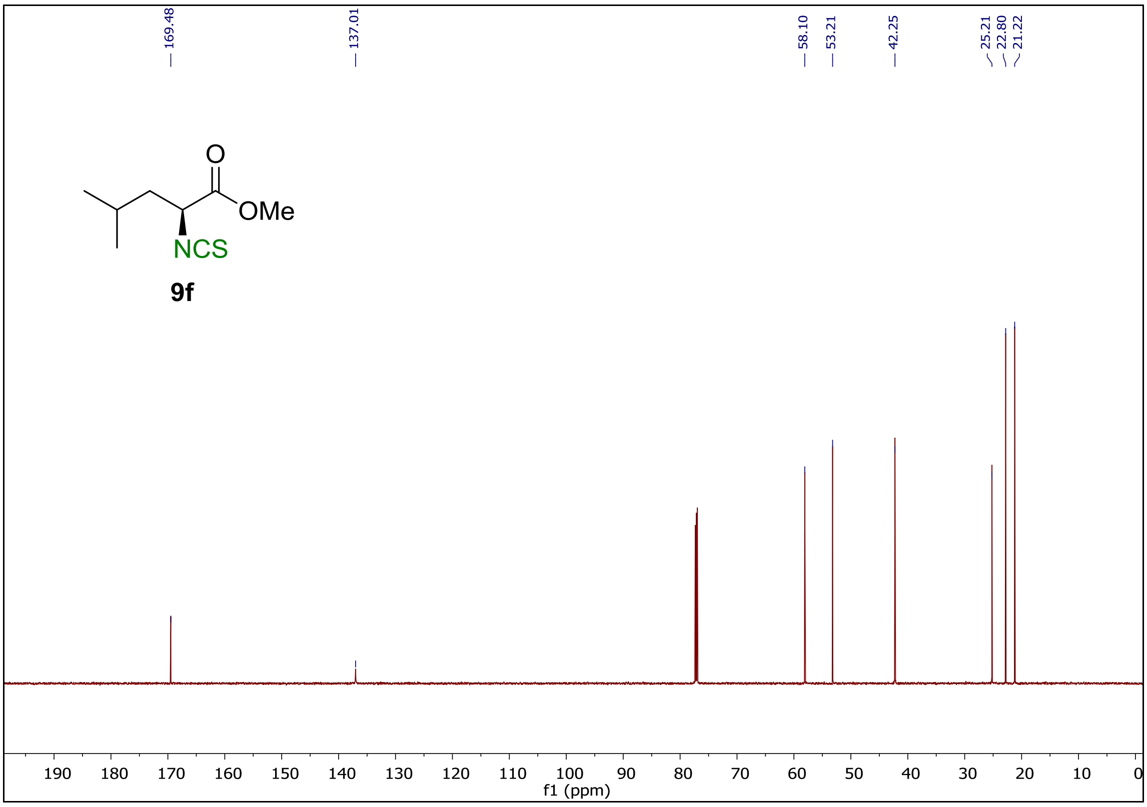

Fig S52. ^{13}C NMR of compound **9f**

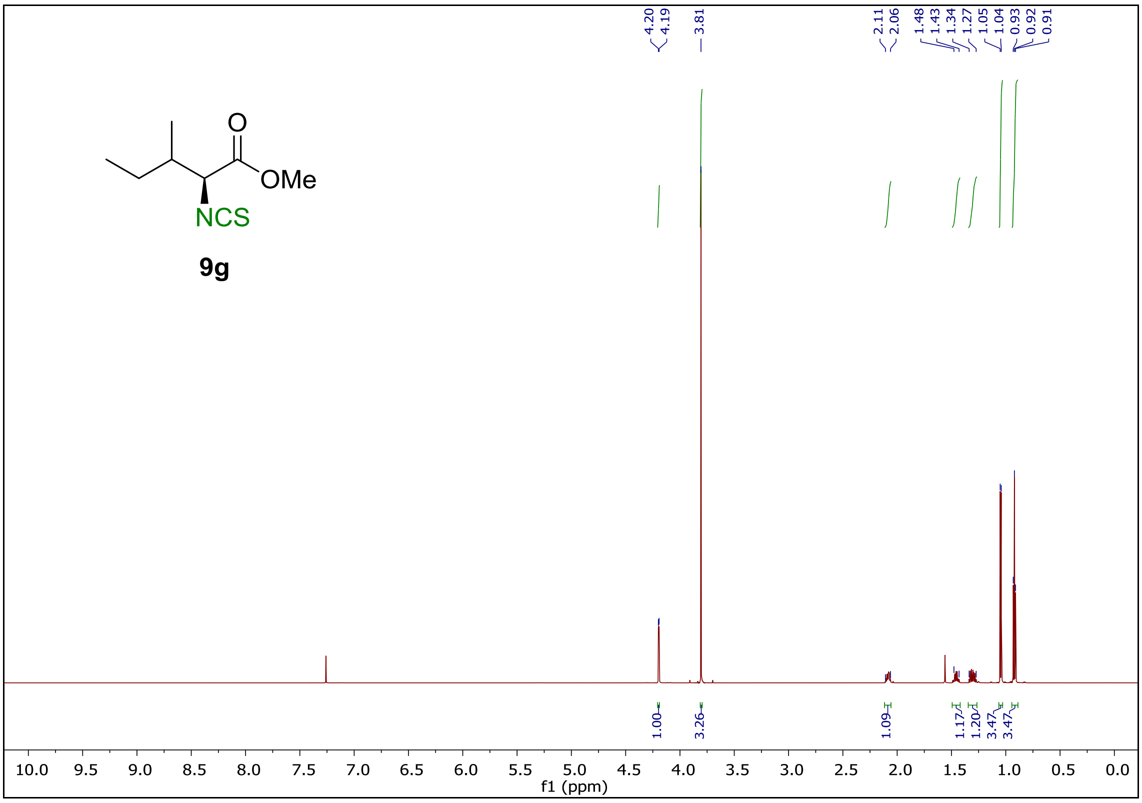

Fig S53. ^{1}H NMR of compound **9g**

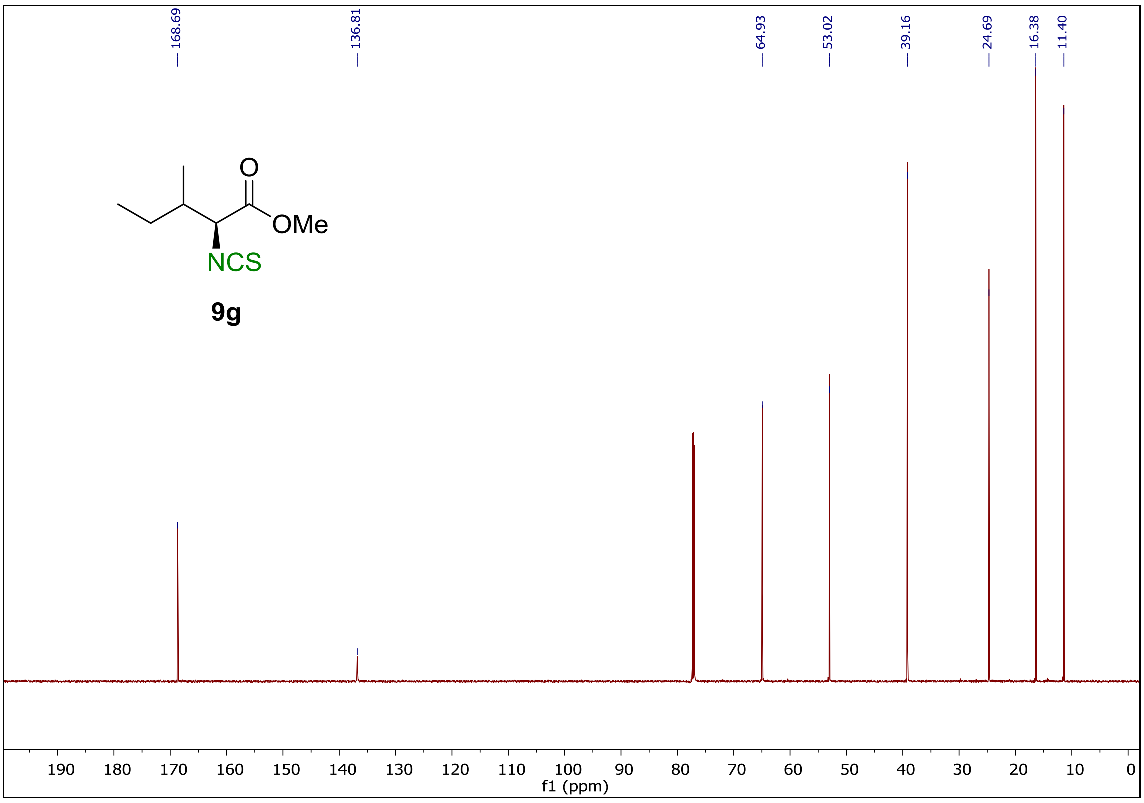

Fig S54. ^{13}C NMR of compound **9g**

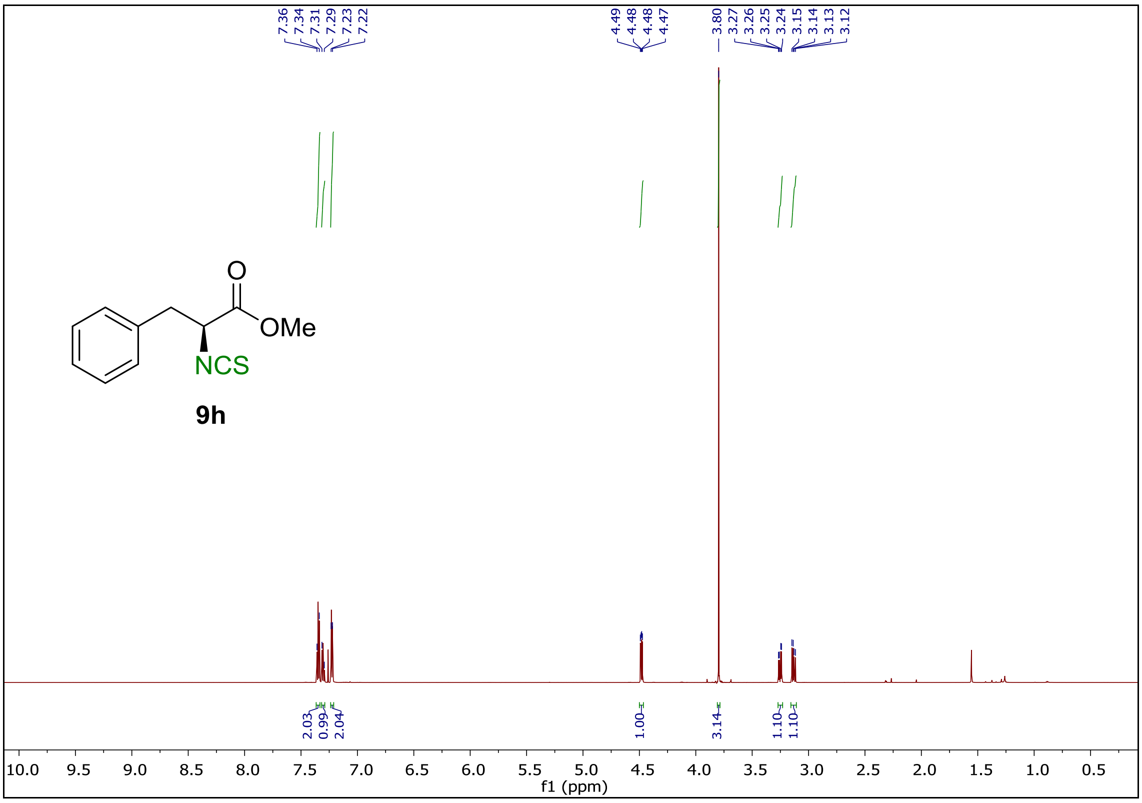

Fig S55. ^{1}H NMR of compound **9h**

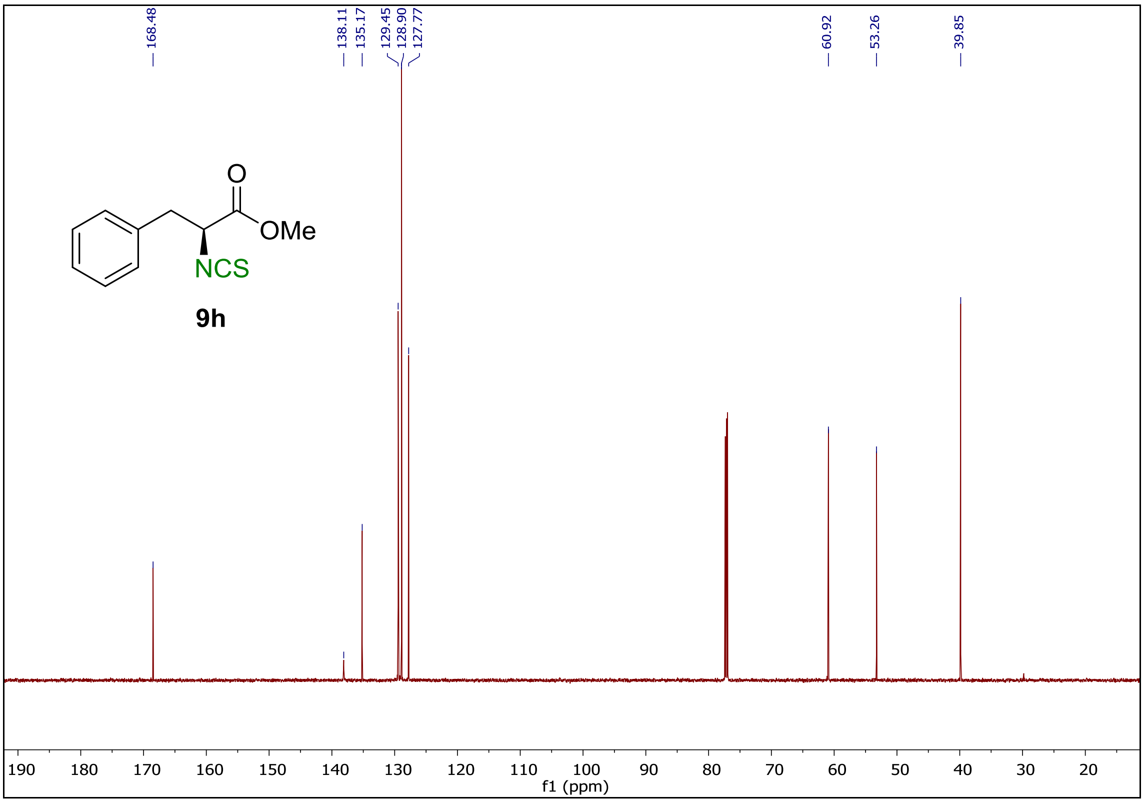

Fig S56. ^{13}C NMR of compound **9h**

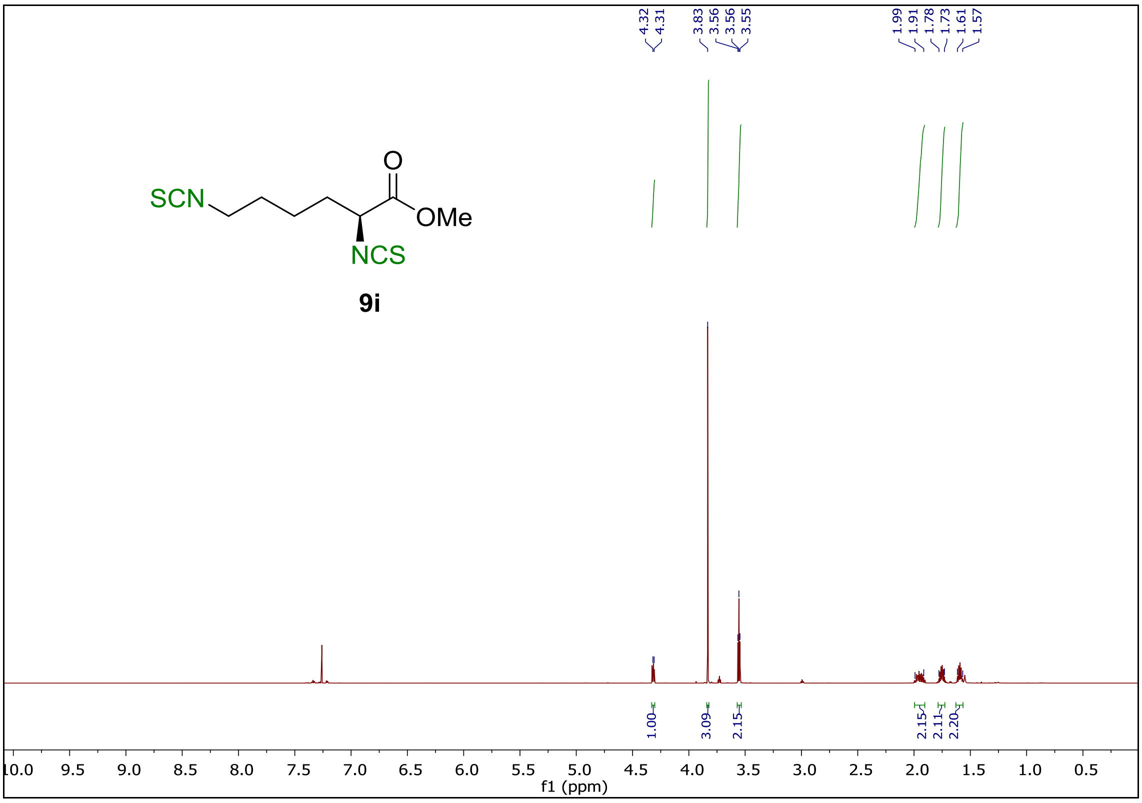

Fig S57. ^{1}H NMR of compound **9i**

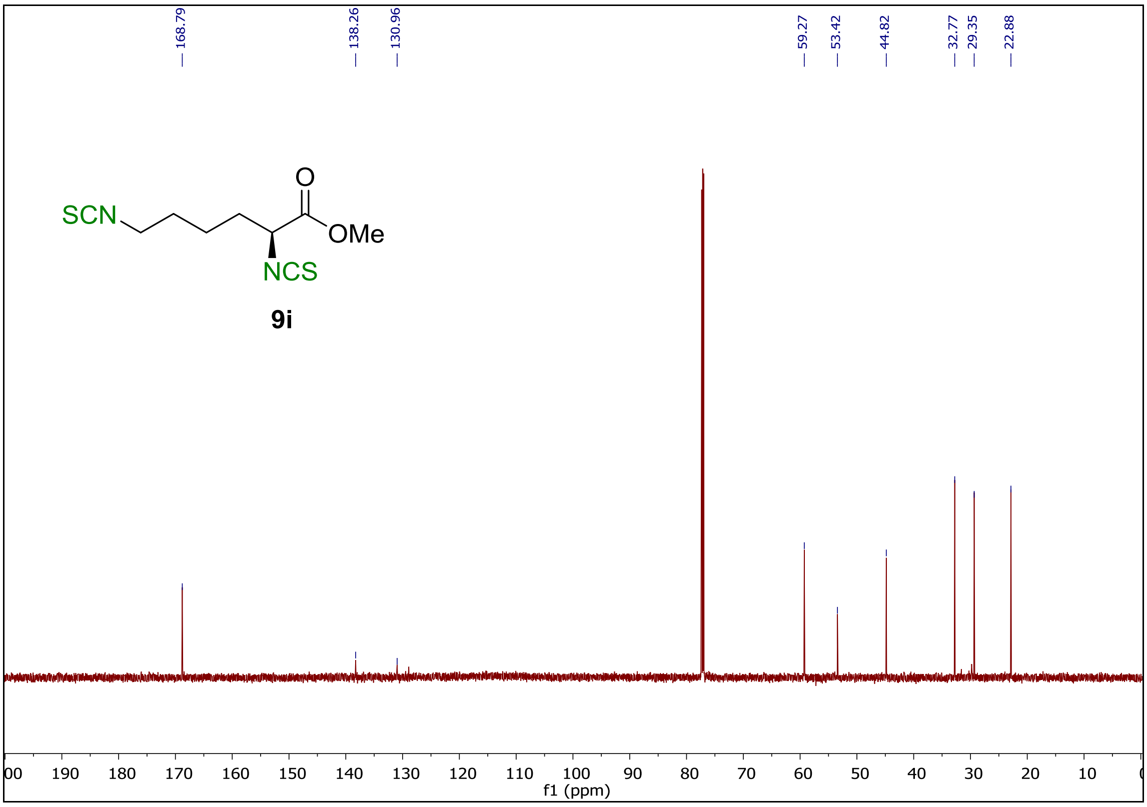

Fig S58. ^{13}C NMR of compound **9i**

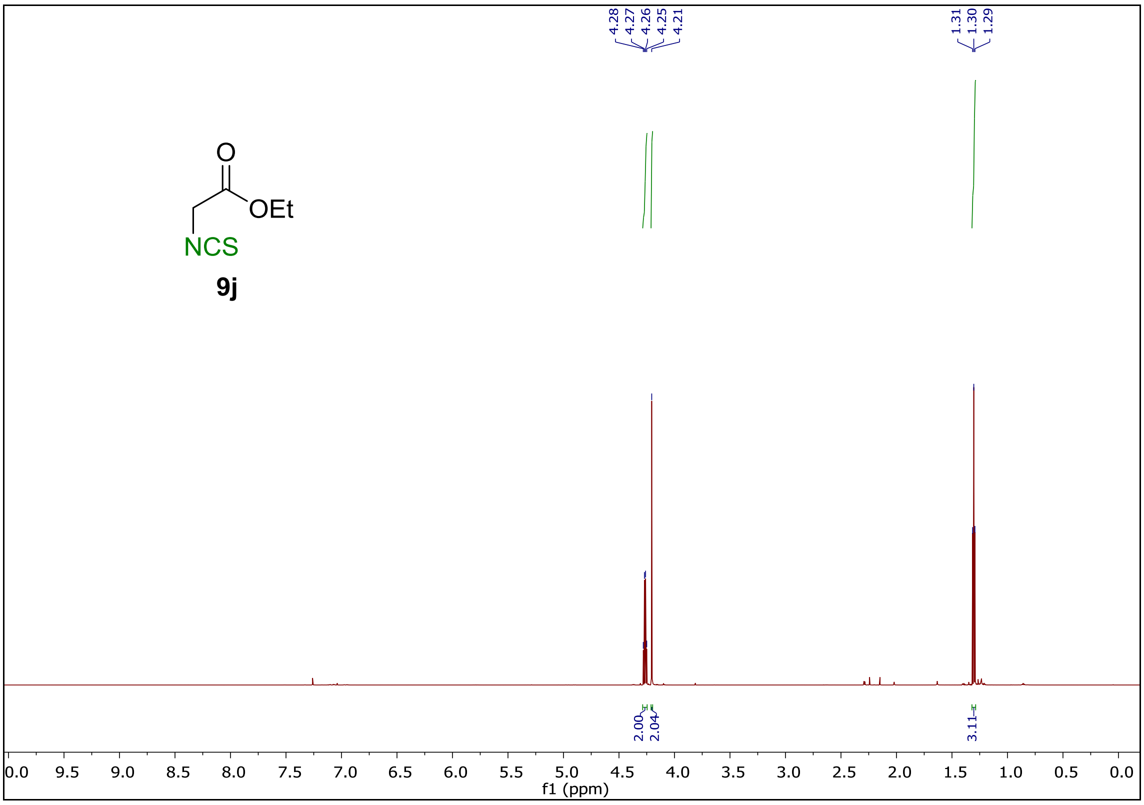

Fig S59. ^{1}H NMR of compound **9j**

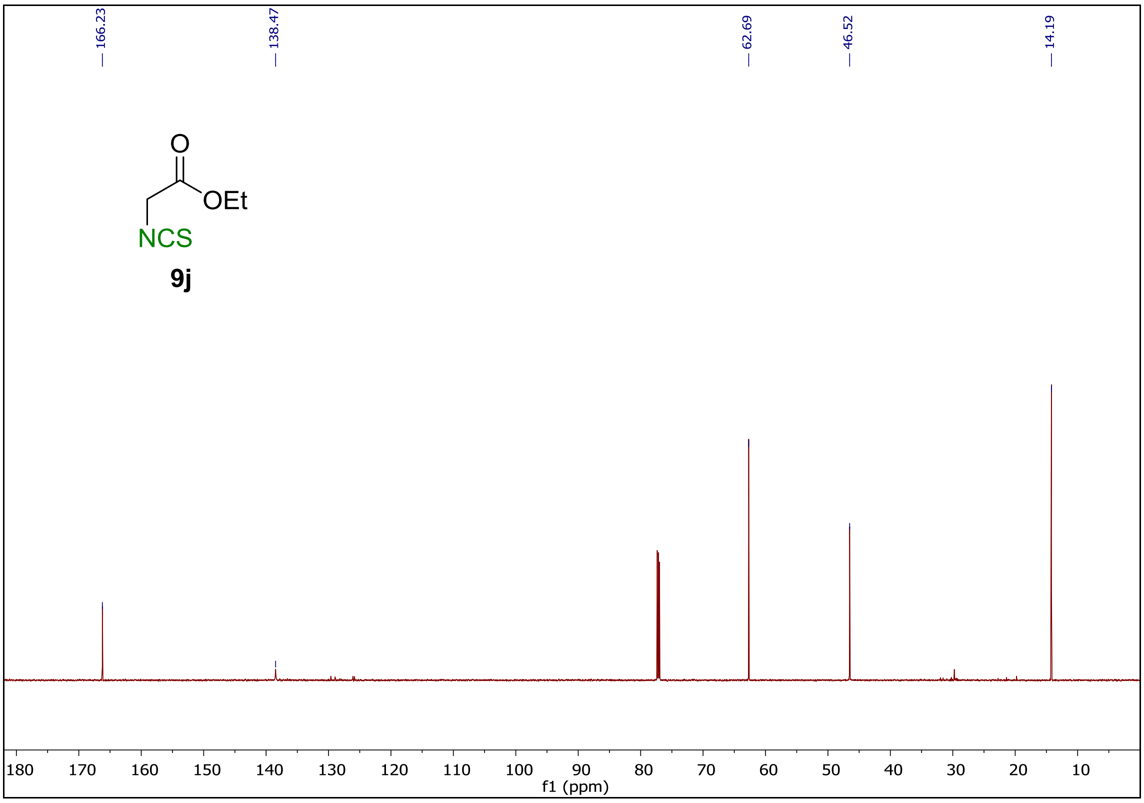

Fig S60. ^{13}C NMR of compound **9j**

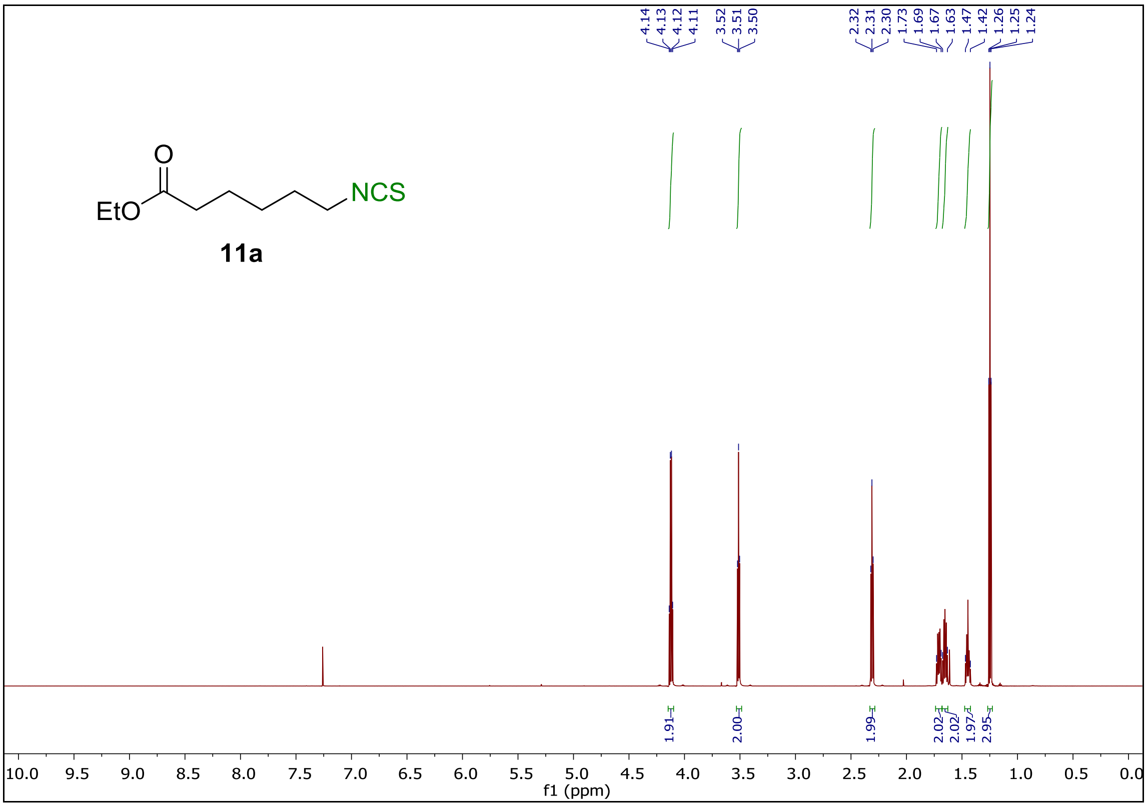

Fig S61. ^{1}H NMR of compound **11a**

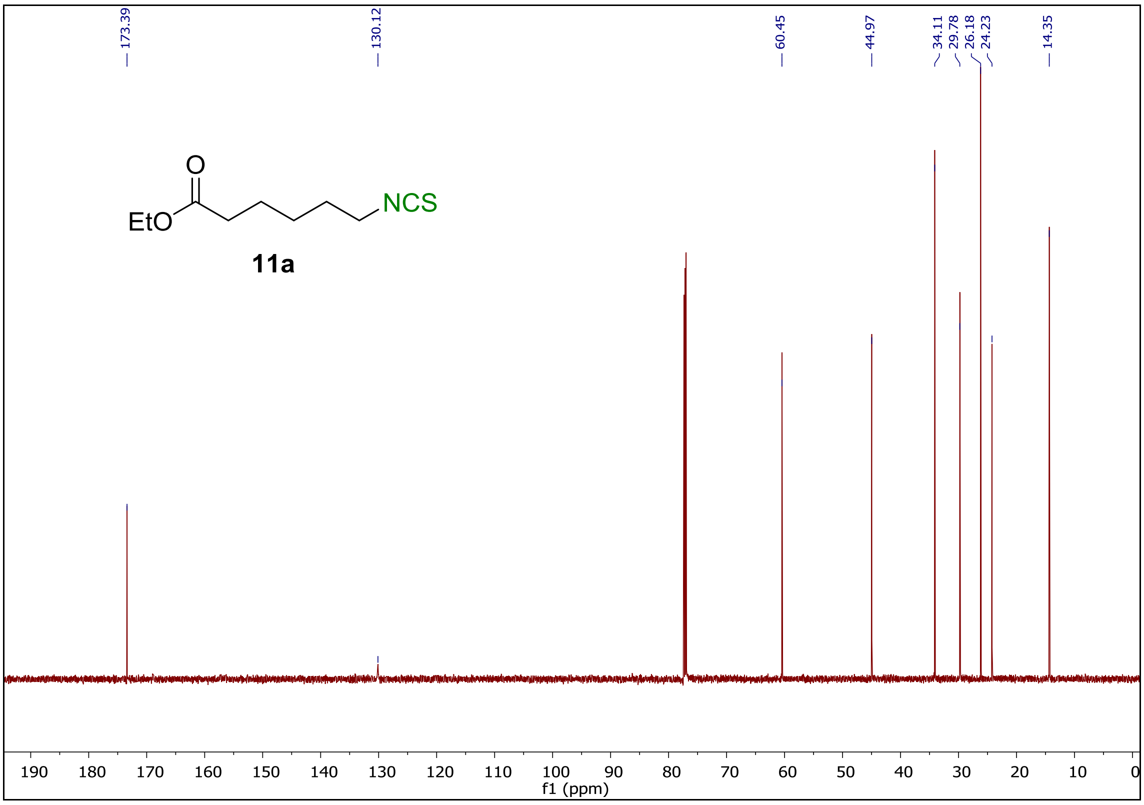

Fig S62. ^{13}C NMR of compound **11a**

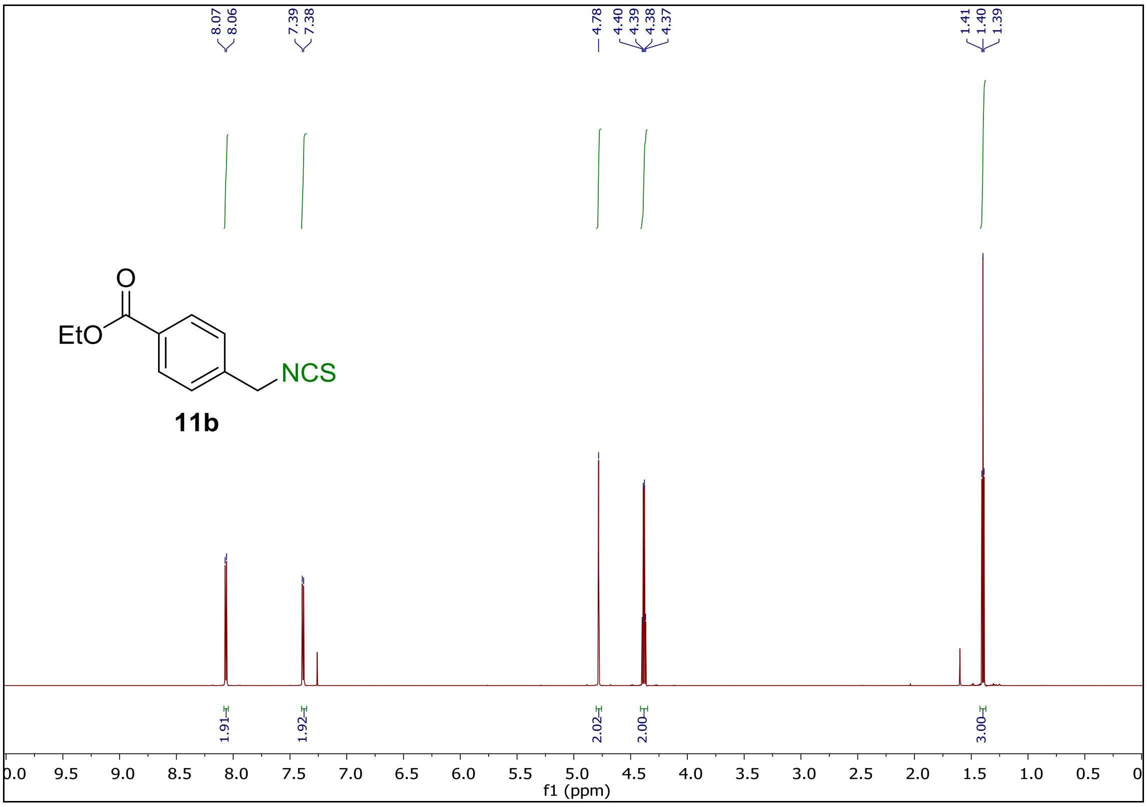

Fig S63. ^{1}H NMR of compound **11b**

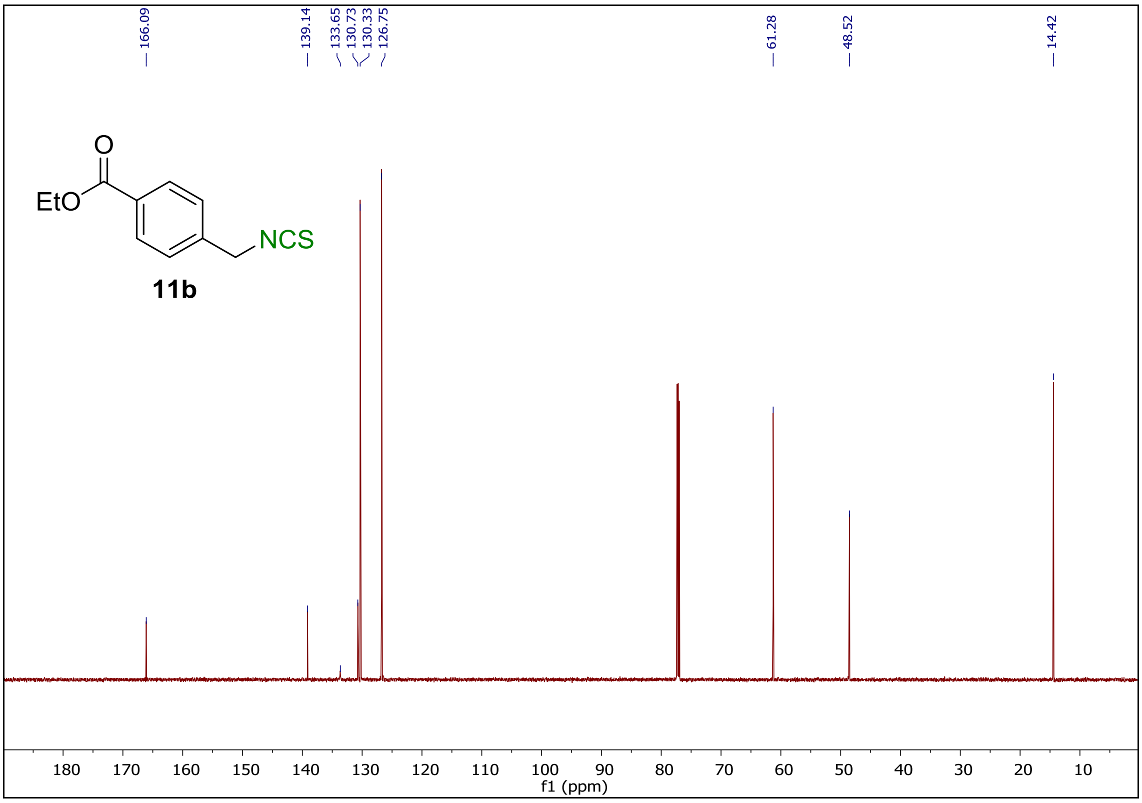

Fig S64. ^{13}C NMR of compound **11b**

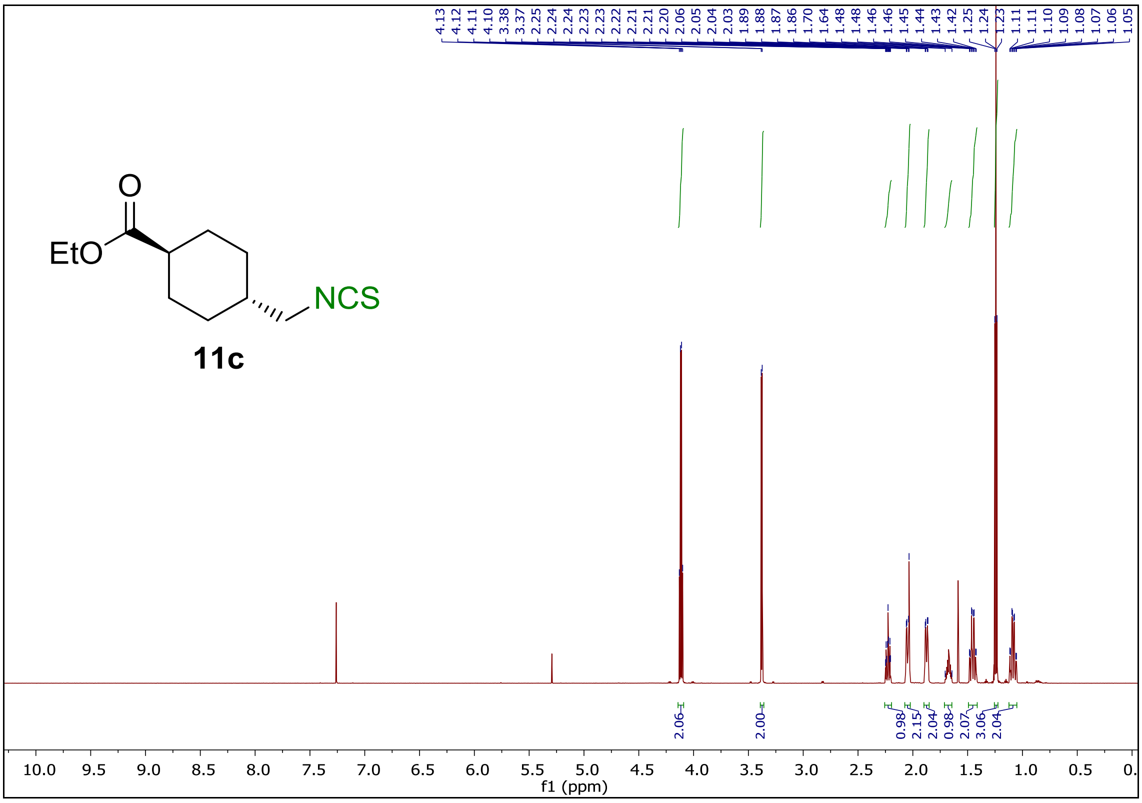

Fig S65. ^{1}H NMR of compound **11c**

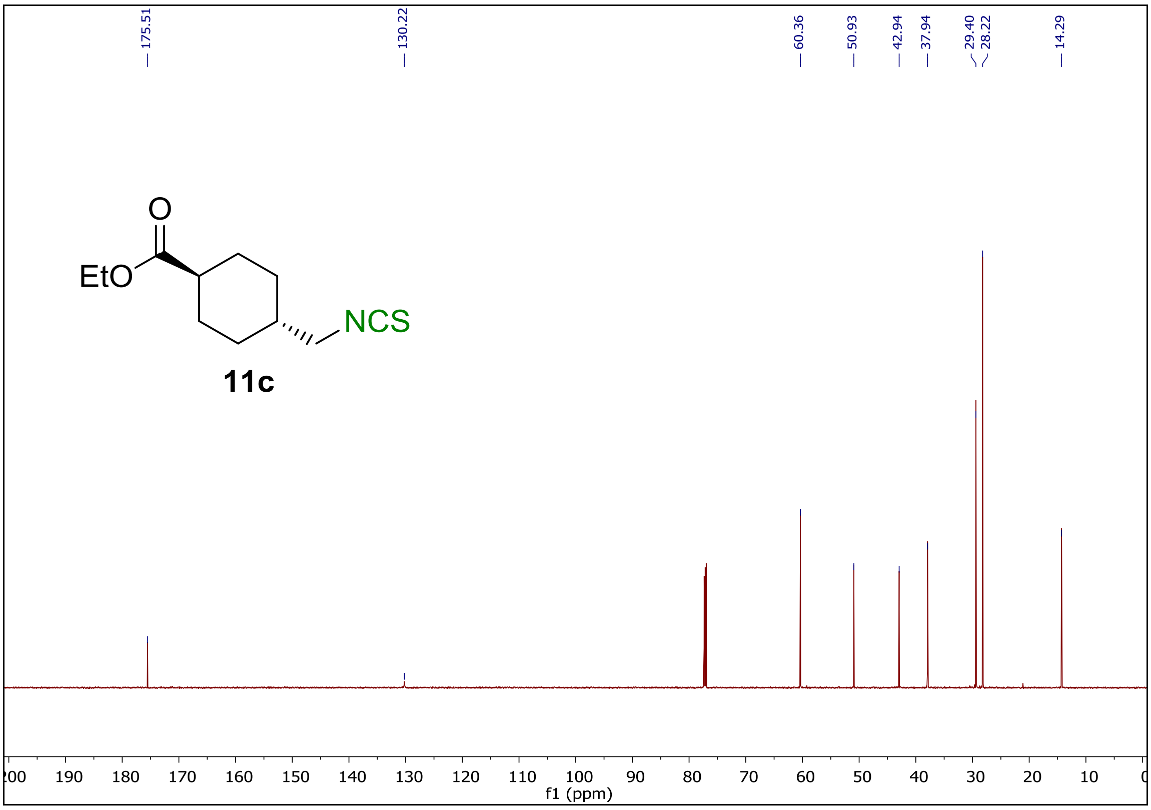

Fig S66. ^{13}C NMR of compound **11c**

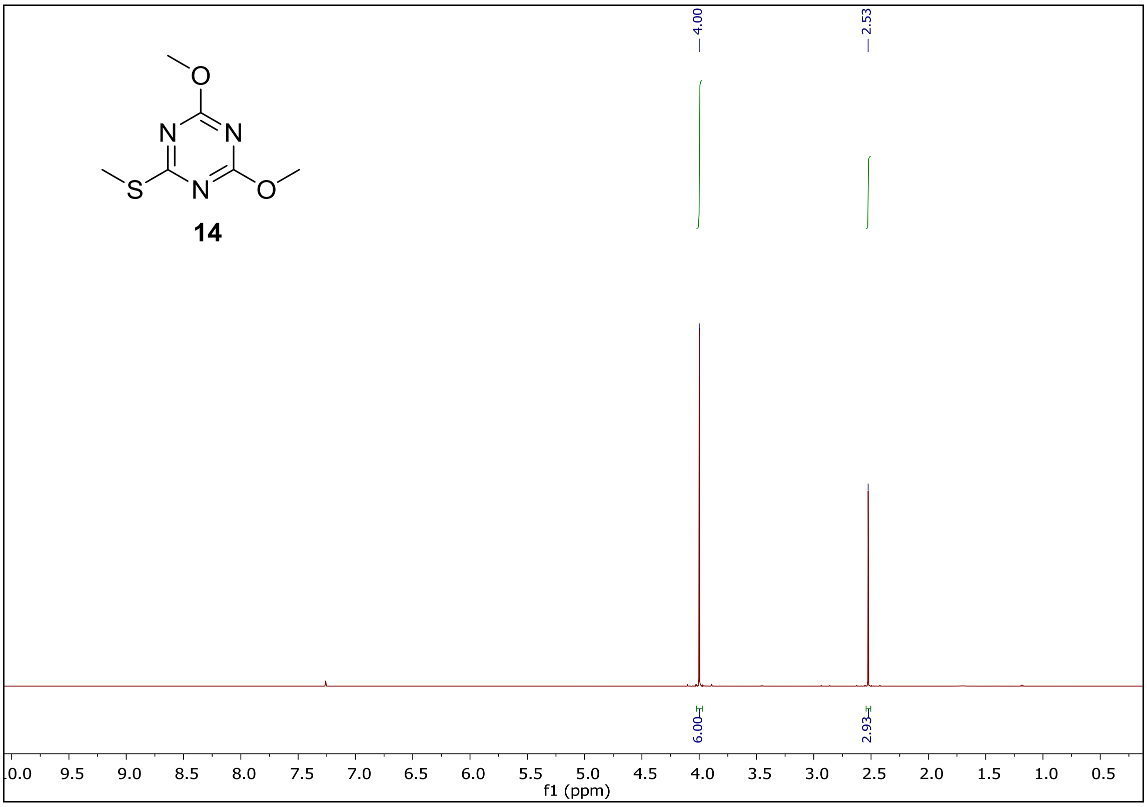

Fig S67. ^{1}H NMR of compound **14**

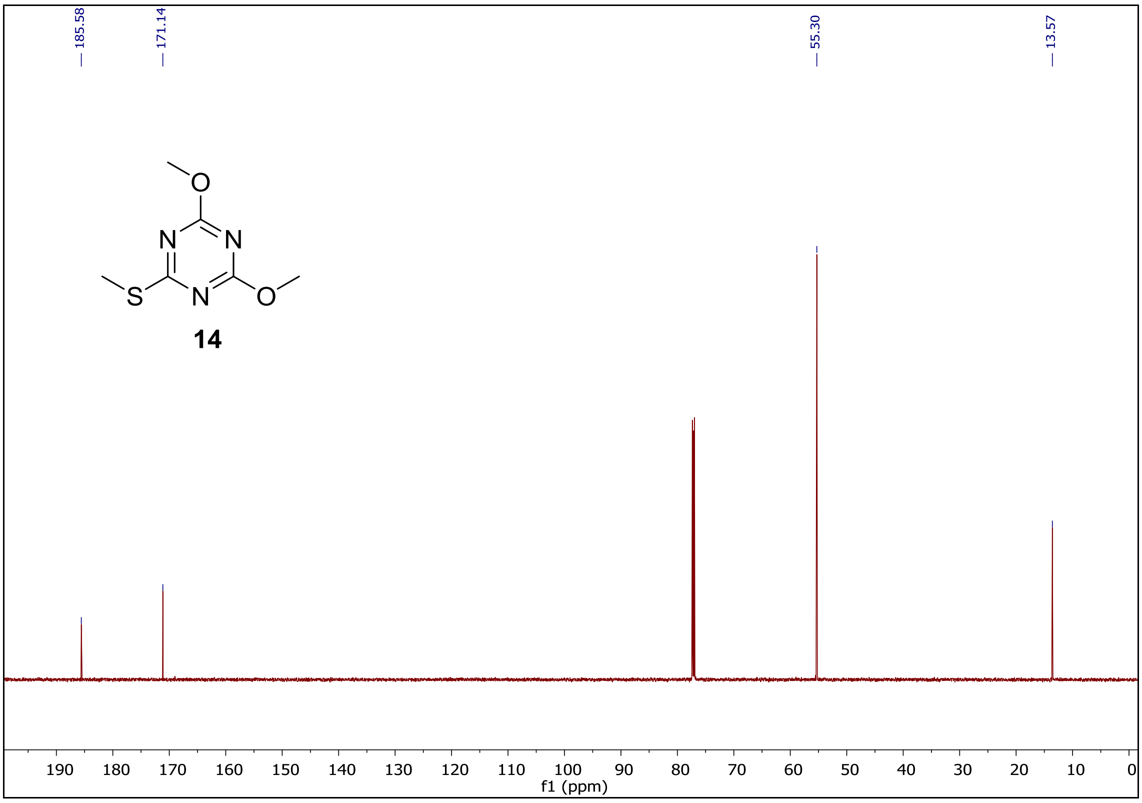

Fig S68. ^{13}C NMR of compound **14**

2. Copies of HPLC chromatograms

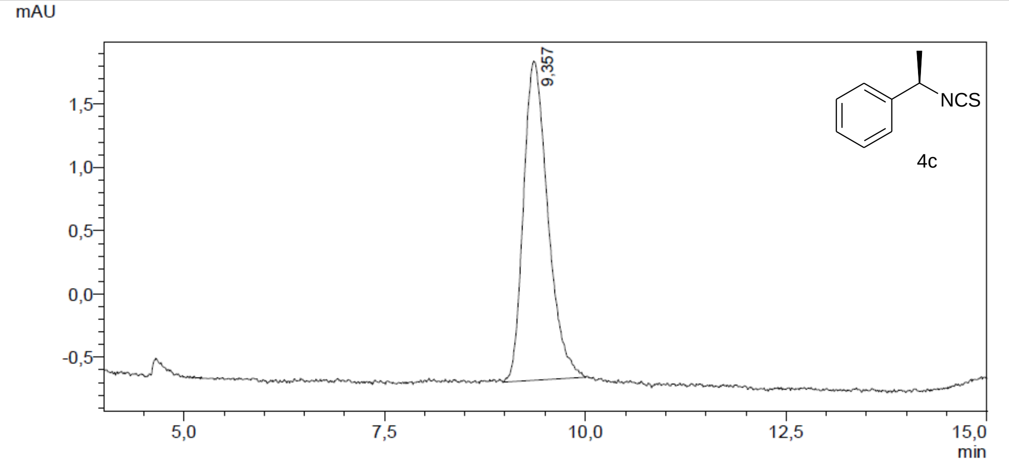

PDA Ch1 254nm

Peak#	Ret. Time	Area%
1	9,357	100,000
Total		100,000

Fig S69. HPLC chromatogram of compound **4c**

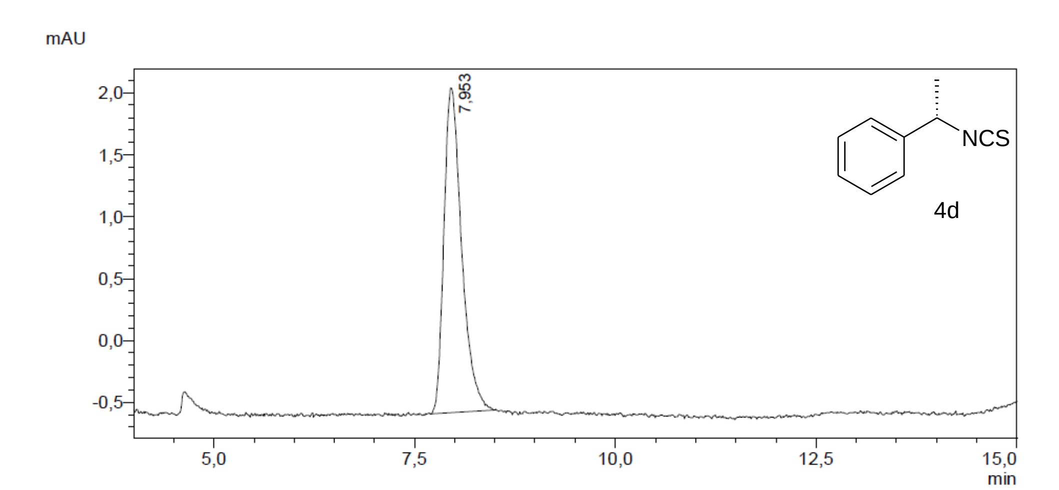

PDA Ch1 254nm

Peak#	Ret. Time	Area%
1	7,953	100,000
Total		100,000

Fig S70. HPLC chromatogram of compound **4d**

Sample Name : LJ-rac_ID_00
Data Filename : LJ-rac_ID_00.lcd
Method Filename : I_00_B_ID_30min.lcm
Date Acquired : 2019-06-04 14:21:28

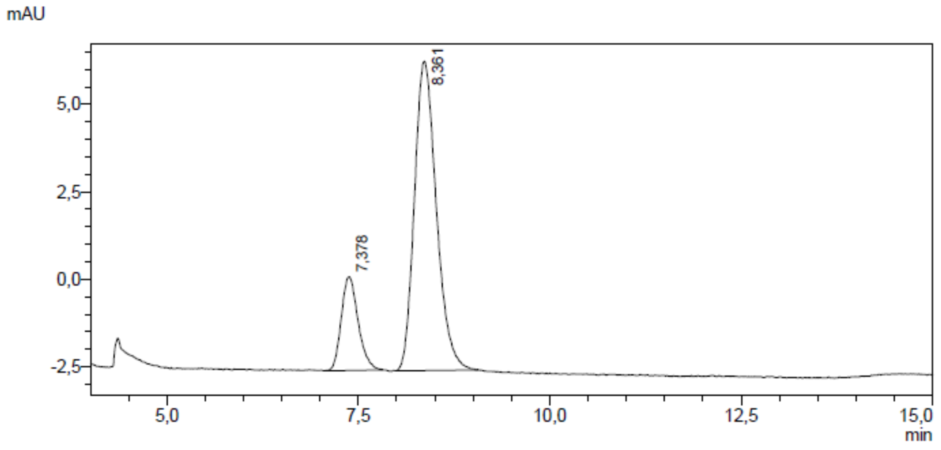

PDA Ch1 254nm

Peak#	Ret. Time	Area%
1	7,378	18,903
2	8,361	81,097
Total		100,000

Fig S71. Chiral HPLC chromatogram of mixture of compounds **4c** and **4d**

Sample Name : LJ-212-IC_98
Data Filename : LJ-212-IC_98.lcd
Method Filename : I_02_B_IC_20min.lcm
Date Acquired : 2019-07-29 14:11:02

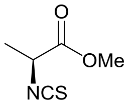

9a

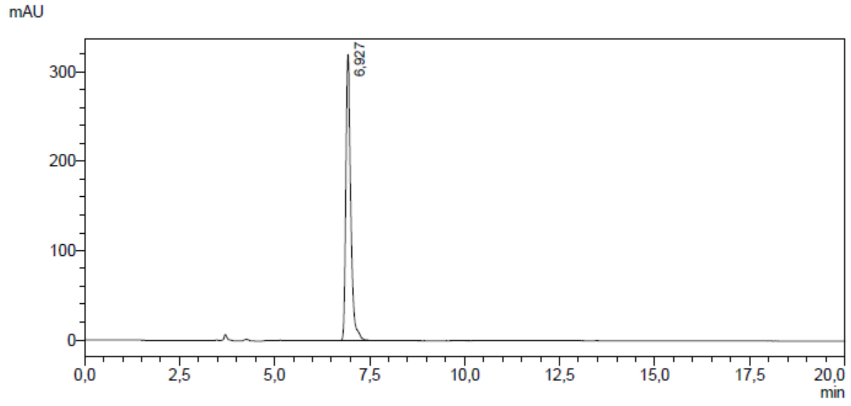

PDA Ch1 254nm

Peak#	Ret. Time	Area%
1	6,927	100,000
Total		100,000

Fig S72. HPLC chromatogram of compound **9a**

Sample Name : LJ-210-IC_98
Data Filename : LJ-210_IC98.lcd
Method Filename : I_02_B_IC_20min.lcm
Date Acquired : 2019-07-29 13:50:31

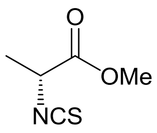

9b

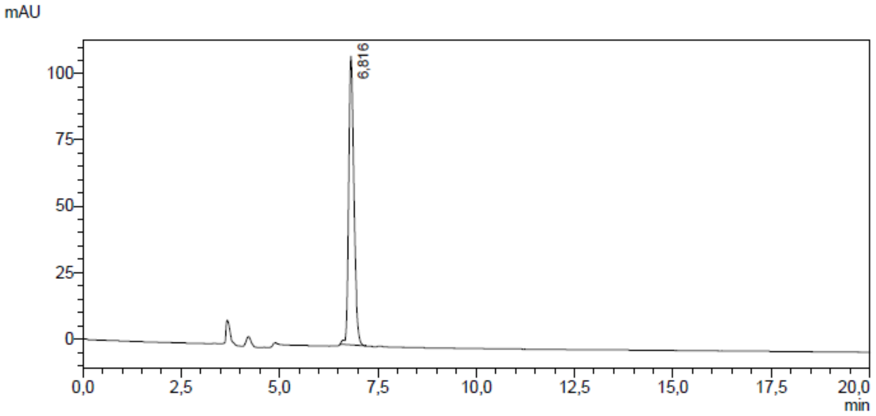

PDA Ch2 266nm

Peak#	Ret. Time	Area%
1	6,816	100,000
Total		100,000

Fig S73. HPLC chromatogram of compound **9b**

Sample Name : LJ-205-IC_98
Data Filename : LJ-205-IC_98.lcd
Method Filename : I_02_B_IC_20min.lcm
Date Acquired : 2019-07-25 14:06:33

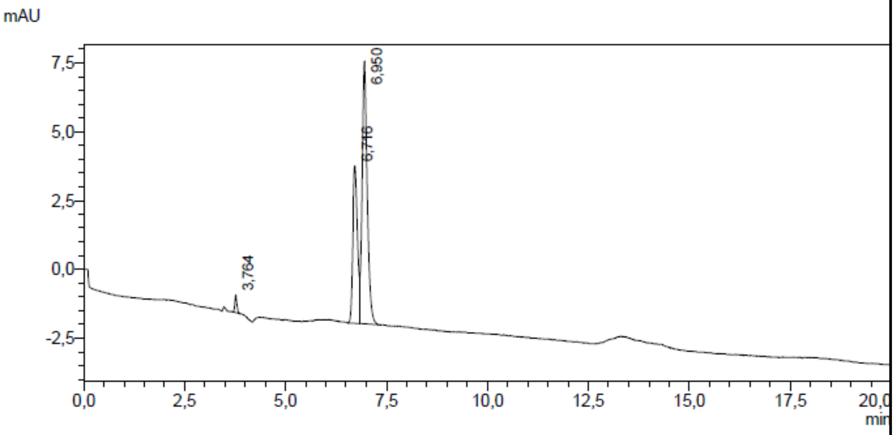

PDA Ch2 266nm

Peak#	Ret. Time	Area%
1	3,764	1,861
2	6,716	34,730
3	6,950	63,410
Total		100,000

Fig S74. HPLC chiral chromatogram of mixture of compounds **9a** and **9b**

3. Copies of CD spectra

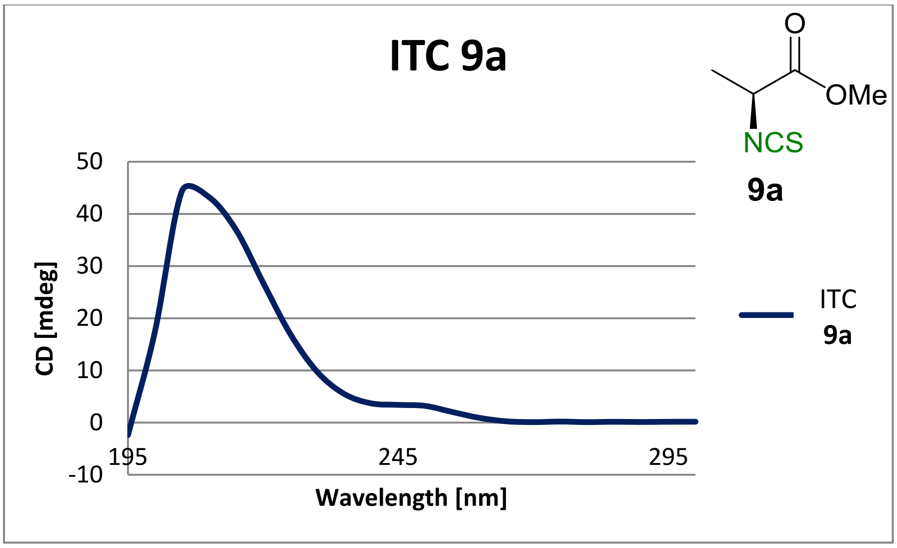

Fig S75. CD spectrum of ITC **9a**.

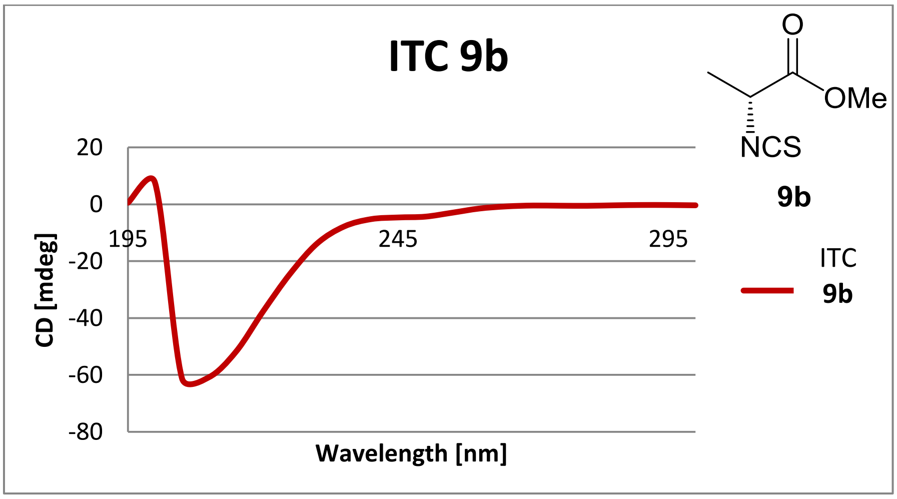

Fig S76. CD spectrum of ITC **9b.**

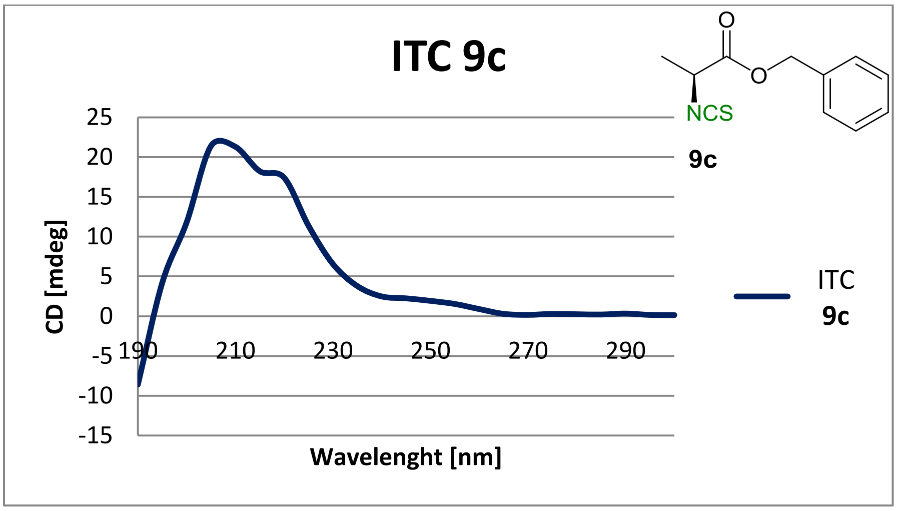

Fig S77. CD spectrum of ITC **9c**.

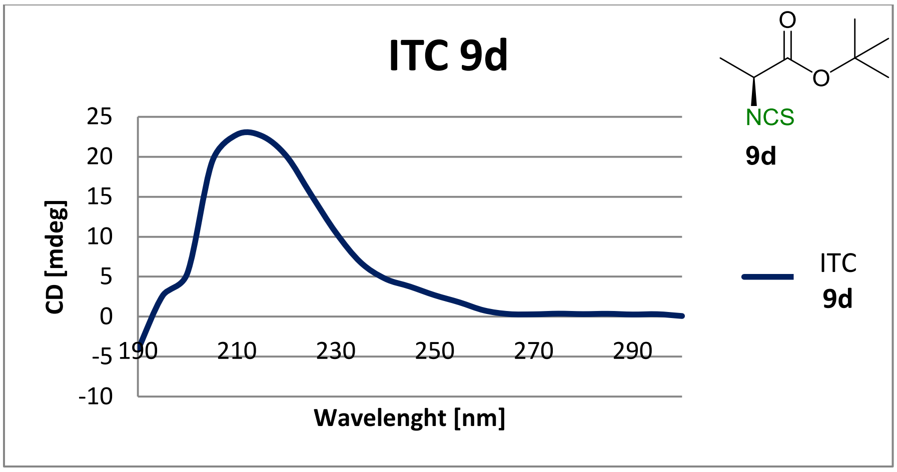

Fig S78. CD spectrum of ITC **9d**.

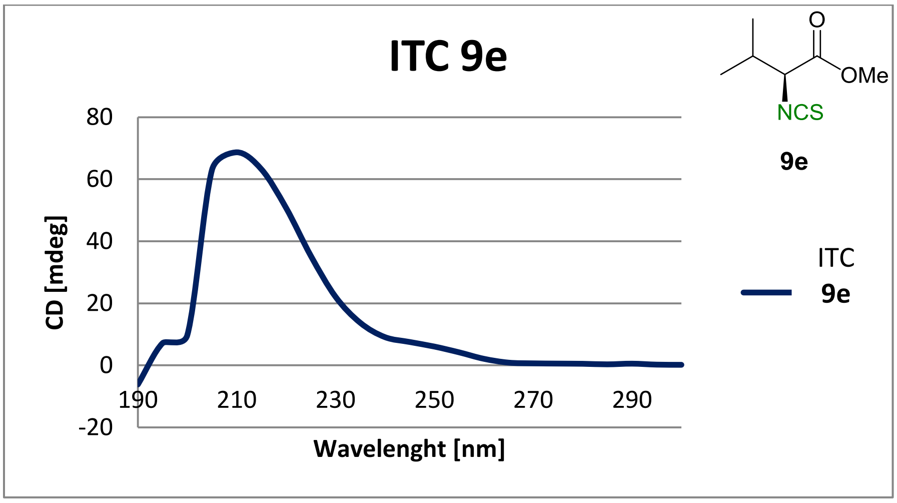

Fig S79. CD spectrum of ITC **9e**.

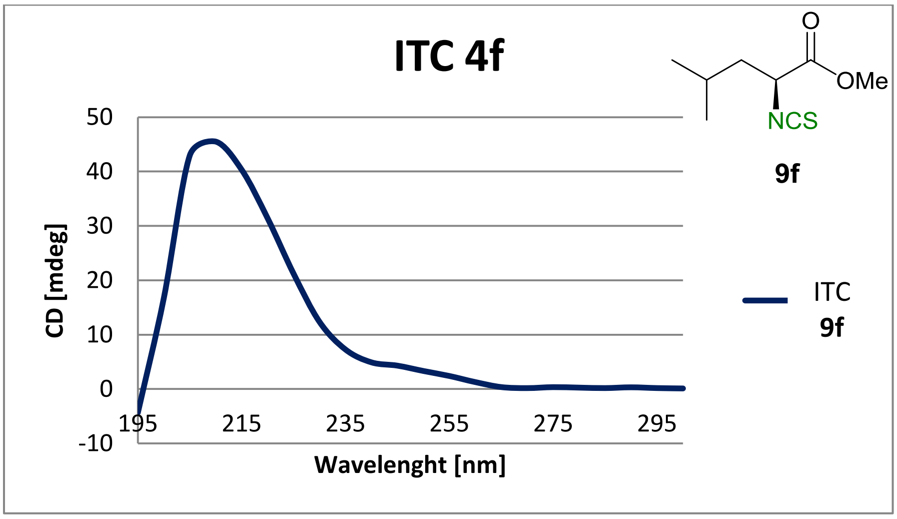

Fig S80. CD spectrum of ITC **9f**.

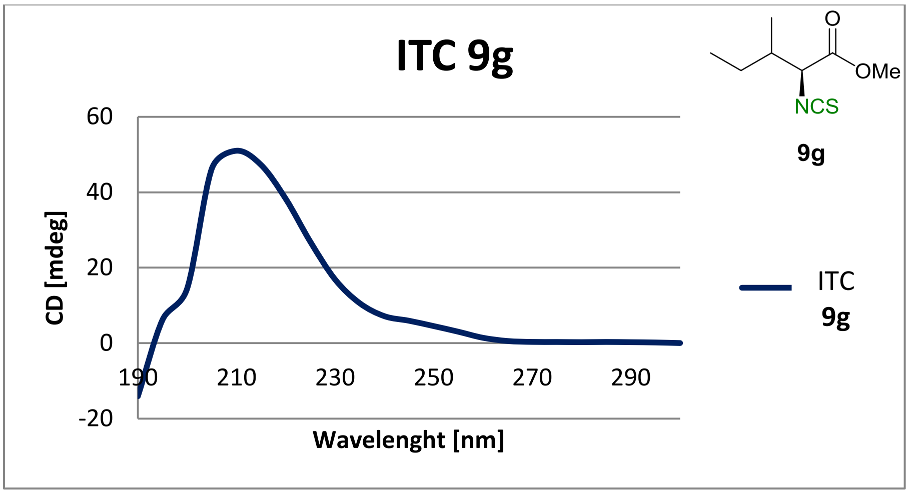

Fig S81. CD spectrum of ITC **9g**.

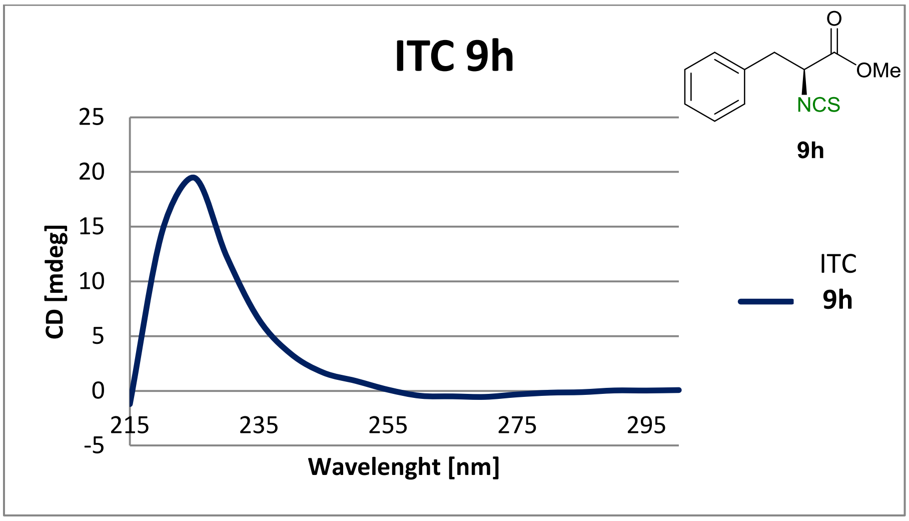

Fig S82. CD spectrum of ITC **9h**.

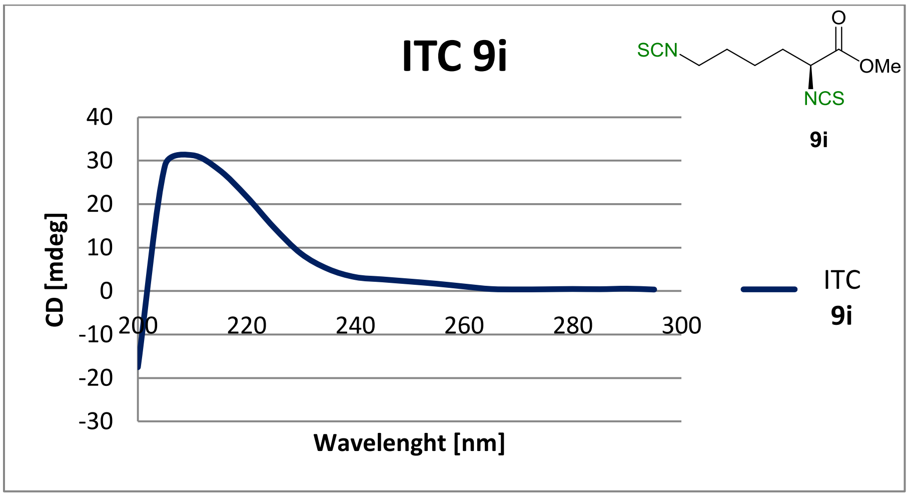

Fig S83. CD spectrum of ITC **9i**.

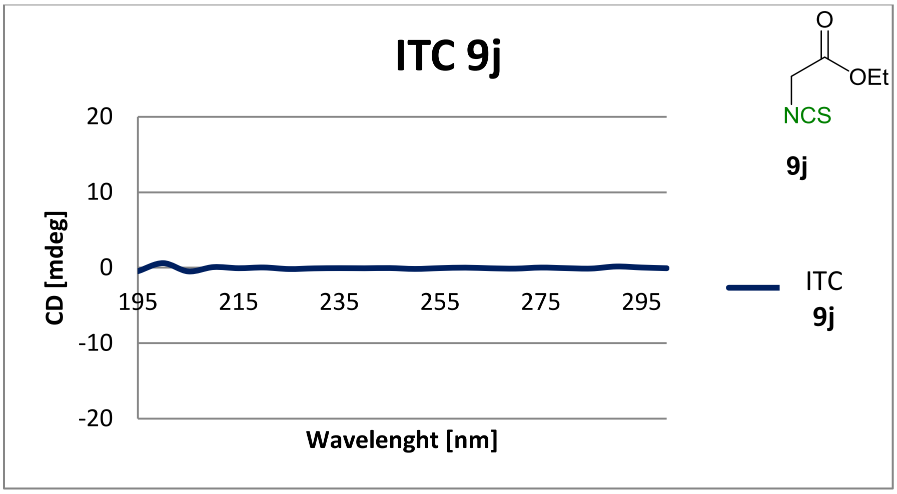

Fig S84. CD spectrum of ITC **9j**.

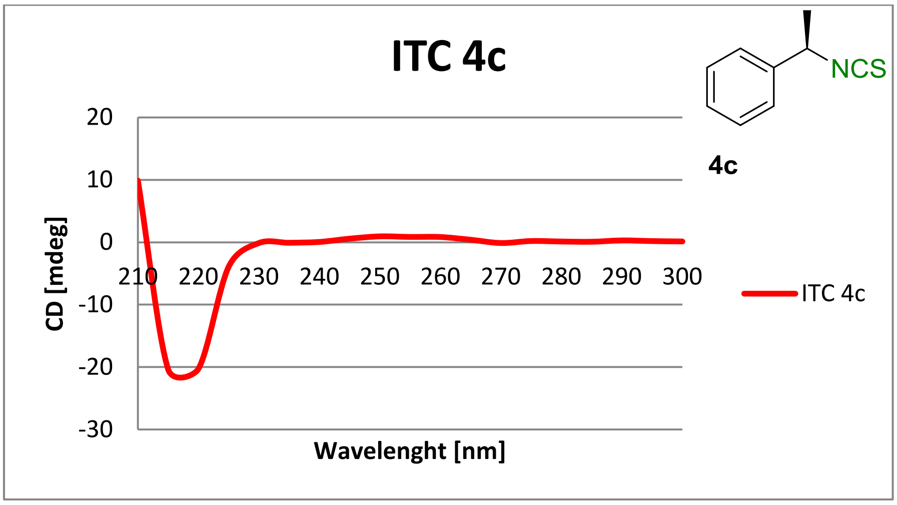

Fig S85. CD spectrum of ITC **4c**

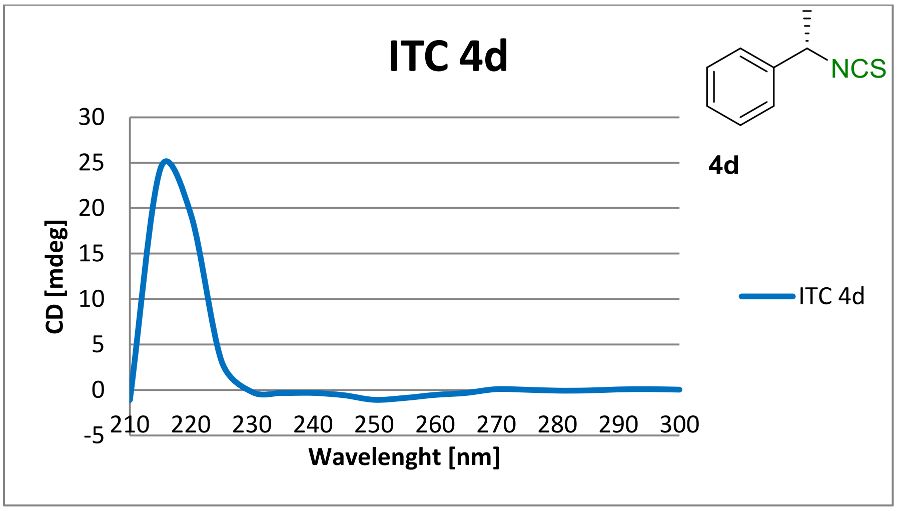

Fig S86. CD spectrum of ITC **4d**

4. Pictures of Petri dishes of antibacterial tests

Staphylococcus aureus (ATCC 6538)

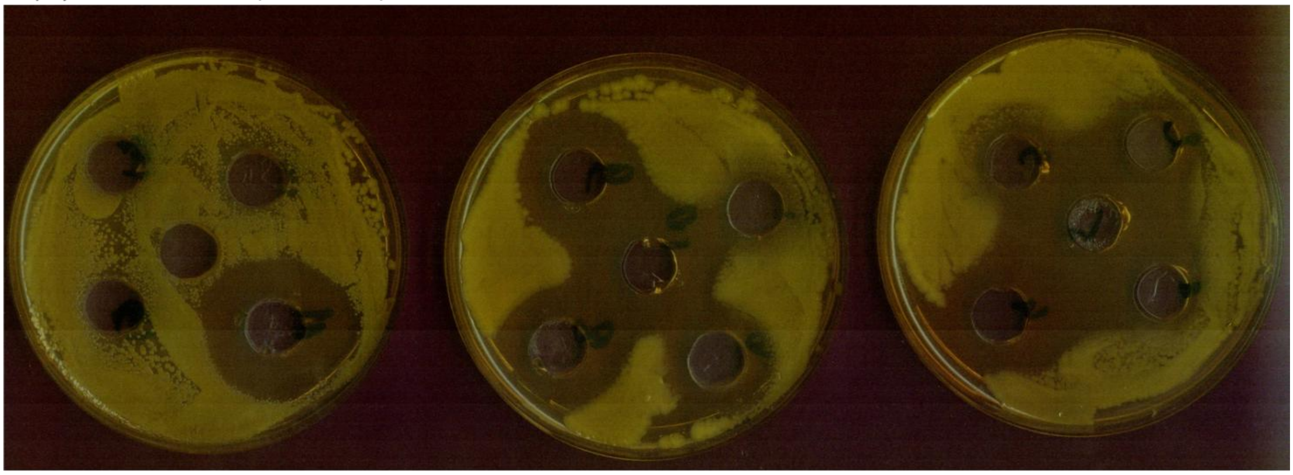

Escherichia coli (ATCC 8739)

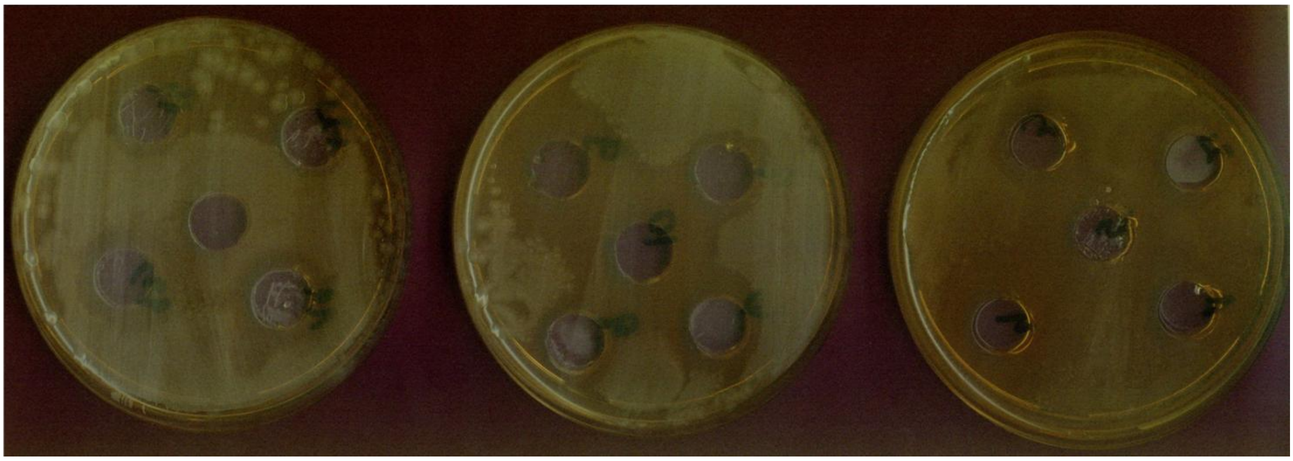

Fig S87. Pictures of petri dishes, tests for antibacterial activity against *Staphylococcus aureus* (ATCC 6538) and *Escherichia coli* (ATCC 8739).
